# Determinants of Visceral Leishmaniasis: A Case-Control Study in Gedaref State, Sudan

**DOI:** 10.1371/journal.pntd.0004187

**Published:** 2015-11-06

**Authors:** Fabienne Nackers, Yolanda Kathrin Mueller, Niven Salih, Mousab Siddig Elhag, Mobarak Elnour Elbadawi, Omer Hammam, Ann Mumina, Atia Abdalla Atia, Jean-François Etard, Koert Ritmeijer, François Chappuis

**Affiliations:** 1 Epicentre, Paris, France; 2 Médecins Sans Frontières, Operational Centre Geneva, Khartoum, Sudan; 3 Federal Ministry of Health, Neglected Tropical Diseases Division, Khartoum, Sudan; 4 State Ministry of Health, Gedaref, Sudan; 5 Institut de Recherche pour le Développement, Montpellier University, Montpellier, France; 6 Médecins Sans Frontières, Operational Centre Amsterdam, Amsterdam, the Netherlands; 7 Médecins Sans Frontières, Operational Centre Geneva, Geneva, Switzerland; 8 Geneva University Hospital, Department of Community Medicine, Geneva, Switzerland; New York University, UNITED STATES

## Abstract

**Background:**

Improving knowledge on local determinants of visceral leishmaniasis (VL) is crucial to guide the development of relevant control strategies. This study aimed to identify individual and household level determinants of primary VL in 24 highly endemic villages of Tabarak Allah hospital’s catchment area, Gedaref State, Sudan.

**Methods:**

From September 2012 to July 2013, in an unmatched case-control design, 198 patients with primary VL were compared to 801 controls free of VL symptoms and with a negative VL rapid test. Using random spatial sampling, controls were selected with a distribution of age, sex and village of residence proportionate to the distribution of the target population. Data were collected using a structured questionnaire.

**Results:**

Children and men were at higher risk of VL. Reporting VL patient(s) in the household in the previous year was the strongest VL risk factor. In a multivariate analysis, VL risk increased with household size, sleep location (outside the yard, not in the farm), evening outdoor activities in the rainy season (playing, watching TV, radio listening), use of ground nut oil as animal repellent and of smoke of Acacia seyal as indoor repellent, presence of dogs in the yard at night, Acacia nilotica in the yard’s immediate surroundings and of a forest at eye range. VL risk appeared to decrease with the use of drinking water sources other than the village water tank, a buffer distance from the adjacent house yard, and with the presence of animals other than dogs in the yard at night. In contrast with previous studies, housing factors, mosquito-net use, black cotton soil, ethnicity, socioeconomic index, presence of Balanites aegyptica and Azadirachta indica in the yard were not independent VL determinants.

**Discussion and conclusion:**

Although these results do not provide evidence of causality, they provide useful suggestions for guiding further intervention studies on VL preventive measures.

## Author Summary

Visceral leishmaniasis (VL), a fatal disease without treatment, is caused by a parasite (*Leishmania*) transmitted to humans through sandflies. Its epidemiology and the vectors involved in its transmission differ importantly between endemic zones. Preventive measures aim at reducing the parasite reservoirs and at limiting human exposure to sandflies. In Sudan, there is a lack of knowledge on VL risk and protective factors, limiting the possibility of development of control strategies. We conducted a study in a VL endemic area, Gedaref State (Sudan), to identify individual and household level determinants of clinical VL. We compared several characteristics of VL patients and inhabitants free of VL. We found that having a household member sick with VL in the previous year was the strongest VL risk factor. VL risk also seemed to be influenced by the household size, sleep location, evening outdoor activities, use of some repellents, and proximity to dogs at night. VL risk also appeared to decrease with a buffer distance between adjacent house yard and with the proximity to animals other than dogs at night. Our findings provide useful suggestions for guiding further intervention studies on VL preventive measures.

## Introduction

**Fig 1:**
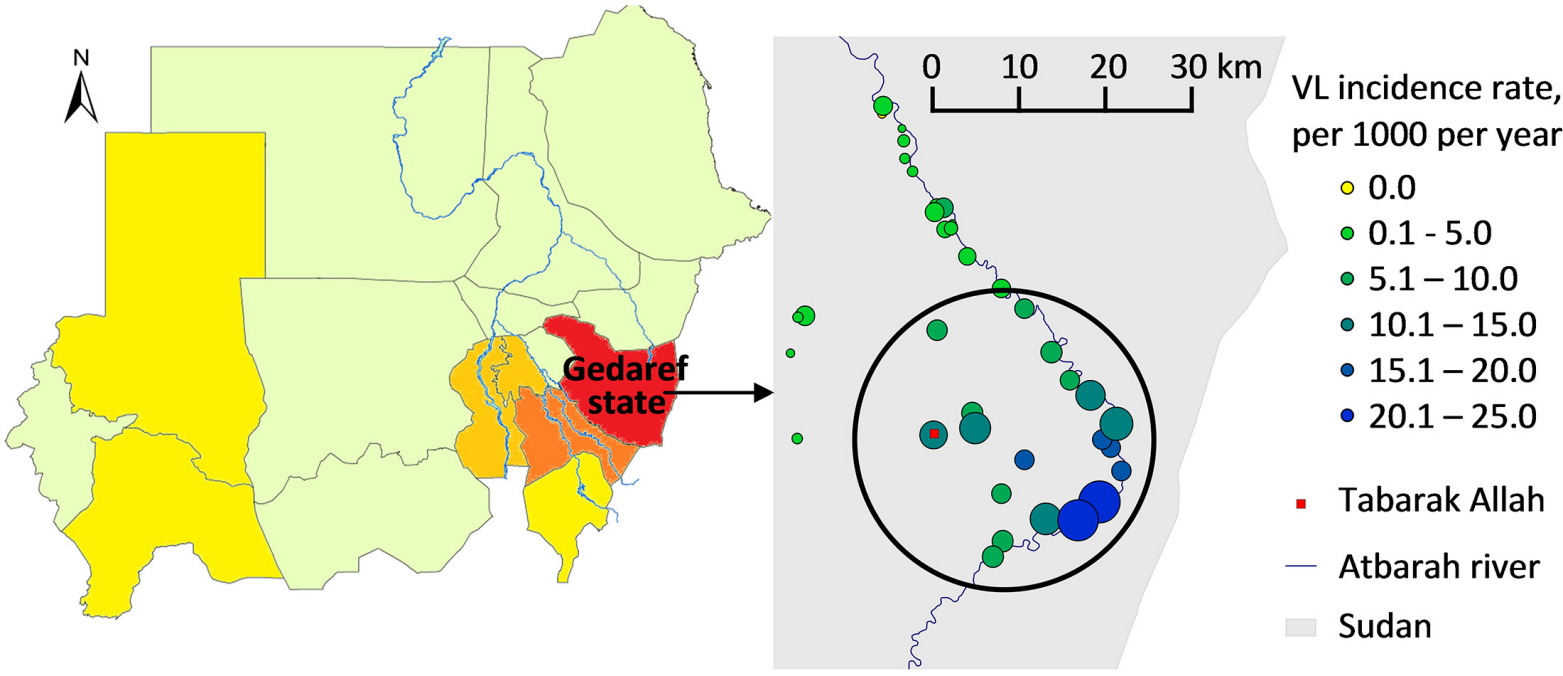
Study area, case-control study on determinants of visceral leishmaniasis, Gedaref State, Sudan, 2012–2013. Left map: Annual incidence of visceral leishmaniasis (VL) in Sudan (2011) from < 5 cases /100,000 inhabitants (yellow) to ≥ 15 cases /100,000 inhabitants (red) (Source: Annual Report, Federal Ministry of Health, Sudan). Right map: Annual incidence of VL in Sudan (2011) proportionate to the size and colour of the circles (from 0 to ≥ 20 cases /1,000 inhabitants). The black circle includes the 24 study villages.

Visceral leishmaniasis (VL) is a devastating illness with a high case fatality rate when left untreated. Gedaref state is the main endemic area of VL in Sudan where the detection figures vary largely between villages in relation with average rainfall and altitude [1]. Villages with higher incidence are clustered along the Atbarah and Rahad rivers [1], near the Ethiopian border. Since 2009, Médecins Sans Frontières (MSF) is offering diagnosis and treatment for VL patients in Tabarak Allah, near the Atbarah river (Fig 1).

In Eastern Sudan, VL is caused by the parasite *Leishmania donovani* and transmitted through the sandfly *Phlebotomus orientalis* [2,3]. *P*. *orientalis* sandfly populations tend to peak at the beginning of the rainy season [2,3] and are concentrated in areas with high densitiy of *Acacia seyal* (locally named “Taleh”) and *Balanites aegyptica* trees (“Lalob” or “Higleeg”) that grow on vertisols (black cotton soil) [4]. *P*. *orientalis* is believed to bite mainly outdoors, in the evening or in the early morning [4], though some populations of the vector may be more adapted to indoor biting [5]. Although some animals, especially dogs [6], have been shown infected by *L*. *donovani* in eastern Africa, their role in transmission is unclear and the disease is believed to be mainly anthroponotic [7,8]. In Sudan, post-kala-azar dermal leishmaniasis (PKDL) cases, corresponding to some 50% of treated cases, might act as a reservoir for parasites and play a role in human-to-human transmission [9,10]. Host factors increasing susceptibility to VL include malnutrition and HIV [11].

In the absence of a vaccine, VL preventive measures aim at reducing the parasite reservoirs (human and, where relevant, animal) and at limiting human exposure to sandflies notably through vector control [12]. Risk factors for VL transmission have been studied in Asia, where poverty and housing conditions appeared to consistently influence the risk of VL [13,14]. However, the important continental differences in terms of reservoir, ecology, parasites and vectors involved in VL transmission limit the generalizability of these results to eastern Africa. In Ethiopia [15–17], Kenya [18] and Uganda [19], factors such as proximity to dogs, sleeping outside, under an acacia tree, or in a thatch house, and a low socio-economic status were reported as possible risk factors for clinical VL. In Sudan, younger age, male gender, and ethnicity [20,21] appeared associated with an increased risk of VL while *Azadirachta indica* (locally named “Neem”) [21] and use of bednets [15,22] appeared offering some protection. To date in Sudan, VL transmission and its possible individual VL risk factors remain poorly understood. However, further knowledge of local VL determinants is essential to design appropriate control activities. Therefore, we conducted a case-control study in endemic villages of Tabarak Allah area in order to identify individual and household level determinants of primary VL.

## Materials and Methods

### Study setting and target population

The study was conducted in the catchment area of the MSF Tabarak Allah Hospital, in Al-Gureisha locality, the main provider of VL treatment in the area. The study target area included the 24 villages (out of 45 villages) most affected by VL according to a survey on burden of VL undertaken in 2011 [23], and closest to Tabarak Allah (Fig 1). The population of the study area was estimated at 46,564 individuals in June 2011, with a VL incidence rate estimated at 9.8/1000 person-years from June 2010 to June 2011 [23]. The vegetation consists in dry savannah woodland (with *Acacia seyal* and *Balanites aegyptica* trees) combined with black cotton soil. The rainy season usually lasts from June to October.

### Study design and sample size

This was an unmatched case-control study. For practical reasons, we expected to enrol cases and controls over a single dry season. Based on the VL incidence rate estimated from June 2010 to June 2011 [23] and accounting for patients that would be missed by the hospital-based passive recruitment, we expected to recruit a bit less than 300 cases. A sample size of 270 cases and 810 controls allowed to detect as significant an odds ratio (OR) of 1.6 with an expected 20% of exposure among controls and a power of 80% (α: 0.05, two-sided).

### Sampling procedures

Cases were recruited in Tabarak Allah Hospital. All successive patients presenting clinical signs of VL (*ie* history of fever of two weeks or more; and either splenomegaly, lymphadenopathies or wasting) and who tested positive to a rk39-based rapid test (BioRad IT-Leish) [24] and/or a direct agglutination test (DAT; titre ≥1:6400) [25,26] and/or had a positive lymph node aspirate [27] were eligible for recruitment if they were at least 6 month-old and if they had been resident of a village of the target area since at least one year. Patients previously treated for VL were excluded. All eligible cases (or their caregivers for individuals younger than 15 years) were invited to sign a written informed consent for participating in a structured interview during a home visit in the month following discharge. HIV-co-infection was not systematically assessed but was expected to be low [28].

Controls were selected from the population with a distribution of age, sex and villages of residence proportionate to the distribution observed in a survey in 2011 [23]. Control houses were selected randomly using a geographic sampling procedure. The perimeter of the inhabited area of each village was recorded in the field using global positioning system (GPS) devices and saved as shapefiles. In each village perimeter, a pre-specified number of GPS random points, according to the 2011 population distribution, were generated. Control selection started in the nearest house from each point. If several houses were at the same distance, one house was selected at random (using a random number table). In each selected house, one individual of a pre-specified sex and age category (to fit the 2011 distribution) living in this house since at least one year, free of clinical symptoms of VL (including PKDL) and with no history of previous treatment for VL was randomly selected. After written informed consent, each potential control underwent a BioRad IT-Leish test. Individuals with a positive result were excluded. If the rapid test was negative, a structured interview was conducted. If there was no eligible control in the first house, the control selection was continued in the nearest one. In case of absence, a second visit was planned within the week. The recruitment started with the dry season on the 15^th^ of September 2012 and ended with the beginning of the rainy season on the 15^th^ of June 2013. We aimed to ensure a similar timing of recruitment for cases and controls. Interviews of controls took place between the 22^nd^ September 2012 and the 9^th^ of July 2013 and all villages were regularly visited over the all study period.

### Data collection

Cases and controls were invited to answer a structured questionnaire on possible determinants of VL (S1 File). It was designed for an exploratory approach and it included data on demographic characteristics, socio-economic factors, personal medical history, travel history, housing conditions, daily activities (with a focus on evening activities), description of house yard and immediate surroundings including animal presence, and occurrence of VL in the house or the adjacent house. When relevant, different answers were allowed for the rainy and the dry seasons. The cases and controls were asked to describe their usual behaviour during the current dry season (for cases, before they developed VL symptoms) and the last rainy season. Questionnaires were translated in Arabic and were piloted before the start of the interviews. The interviewers were trained to administer the questionnaires in a standardised way.

### Data analysis

Data were double-entered and analysed using Stata 12 software (Stata Corporation, College Station, Texas, USA). Using a principal component analysis, a socioeconomic index including data of ownership among controls (radio, TV, bicycle, motorcycle, car/truck/tractor, mobile phone, donkey cart, and generator) was built. Each variable in the index was given a coefficient corresponding to eigenvectors of the first and second components, weighted for their respective contribution. The index was divided into quintiles.

Variables were grouped by predefined thematic sections (demographics, travel history, activities and sleeping habits, characteristics of the household, the house, house yard and surroundings) and by season. The thematic sections are presented in the supplementary material (S1 Table), where individual level and household level determinants are distinguished. In each section, the variables associated with VL (p<0.20) after adjustment for age, sex and village taken as a random effect (due to the high number of villages) were combined in mixed multivariate logistic regression models using a stepwise forward approach. Variables associated with VL (p<0.20) from the different sections were then combined to build one multivariate model with potential VL risk factors at the individual level and another multivariate model with household-level risk factors. Variables not associated with VL (p≥20%) were excluded. Finally all variables were combined into a multivariate logistic model in which variables significant at the 5% level were kept. An interaction between age and sex (likelihood ratio test for interaction p = 0.027) was kept in the model. The variables related to the presence of VL patients in the household or the adjacent house(s) were kept separate because they were likely on the causal pathway between VL and the other determinants.

### Ethics statement

Authorization to conduct the study was obtained from the Gedaref Ministry of Health, Humanitarian Affairs Commission, and the village authorities. Ethical clearance was obtained from the MSF ethical review board and National Research Ethics Review Committee of the ‘Health Research Council” (National Ministry of Health). All adult subjects provided informed consent, and a parent or guardian of any child participant provided informed consent on their behalf. Each individual signing the consent form to participate as a control was offered a mosquito net. Individuals with VL clinical symptoms were referred to Tabarak Allah for further diagnostic. Because of the possibility of a not yet clinical infection, asymptomatic individuals with a positive rk39 test were followed-up by phone during 6 months and encouraged to consult Tabarak Allah hospital in case they developed persistent fever in the months following the study.

## Results

Of the 321 primary VL patients admitted in Tabarak Allah during the study period, 208 were coming from the study area, corresponding to a passive detection rate of 6.0/1000 person-years (ranging from 0 to 16.4 person-years across villages). Nine did not live in the same place during the past year and one patient died, resulting in 198 cases interviewed. Of the 849 eligible individuals selected as controls, 3 refused participation, 21 were absents despite two revisits, nine presented VL clinical symptoms and 15 were rK39 positive (of which 4 developed VL during the follow-up, 5 remained asymptomatic, and 6 were lost to follow-up). In total, 801 controls were included in the analysis.

Considering socio-demographic characteristics (Table 1), cases were younger than controls and more often male. A third of cases and controls belonged to the Massalit, other major ethnic groups being Zabarma, Fallata, Hausa (26.2% of controls) and Tama (20.1% of cases). Cases originated mainly from Berber Al-Fugera (29.8%) and Tabarak Allah (21.7%), compared to 21.4% of the controls. There was no evidence for a difference of socio-economic index category between cases and controls. Univariate analysis comparing all cases and controls characteristics is presented in the supplementary material (S1 Table).

**Table 1:** Socio-demographic characteristics, case-control study on determinants of visceral leishmaniasis, Gedaref State, Sudan, 2012–2013.

	Control (N = 801)		Case (N = 198)		
	n	%	n	%	p-value
**Age (years)**					
0 to 9	283	35.3	104	52.5	<0.001
10 to 19	196	24.5	52	26.3	
20 to 39	187	23.4	24	12.1	
40 or more	135	16.9	18	9.1	
**Male**	414	51.7	124	62.6	0.006
**Ethnicity** (60 missing)					
Massalit	263	34.8	57	31.0	<0.001
Zabarma/Fallata/Hausa	198	26.2	24	13.0	
Tama	52	6.9	37	20.1	
Arnga/Gemir	63	8.3	19	10.3	
Sudanese, Arabs	81	10.7	14	7.6	
Sudanese, Non Arabs	52	6.9	22	12.0	
Others	46	6.1	11	6.0	
**Village**					
Berber Al-Fugera	87	10.9	59	29.8	<0.001
Tabarak Allah	84	10.5	43	21.7	
Khuor Zaraf	78	9.7	7	3.5	
Jebel Ghana	63	7.9	24	12.1	
Al-asira	62	7.7	10	5.1	
Um Gzaz	54	6.7	1	0.5	
Birkat Norein	52	6.5	11	5.6	
Mashra Al Forsan	51	6.4	8	4.0	
Wad Arood	41	5.1	0	0.0	
Other	229	28.6	35	17.6	
**Socioeconomic index**					
Lowest	204	25.5	46	23.2	0.475
2nd lowest	255	31.8	54	27.3	
Middle	34	4.24	8	4.0	
2nd highest	185	23.1	52	26.3	
Highest	123	15.4	38	19.2	

After adjustment on age, sex, their interaction and village as random effect, there was no evidence (p≥0.20) for an association between VL and black cotton soil (surrounding the yard of 85.4% of the cases and 74.7% of the controls), proximity of water bodies, housing materials, use of mosquito nets, indoor spraying, travel history, day activities, and long term medical treatment. All variables that remained associated with VL (p<0.20) after adjustment are presented in the Table 2 with OR and 95% confidence intervals (95%CI). Among participants of schooling age or older, cases were more frequently attending school. Between sunset and bedtime, the majority of the participants stayed exclusively outdoor in the dry season (S1 Table) while, in the rainy season, 30.4% of controls and 26.8% of cases stayed indoor only (Table 2). In the rainy season, outdoor evening activities (playing, watching TV/radio listening) were associated with an increased risk of VL compared to staying indoor. Most participants reported to sleep within the house yard, although 2.9% of controls and 7.6% of cases reported to sleep outside the house yard but within the village (for example, some children slept at school). During the rainy season, 15.5% of controls and 8.6% of cases reported to sometimes sleep in the field/farm outside the village. In the dry season, about half of the participants were sleeping outside a room while, in the rainy season, more than 90% of all participants were sleeping inside a room. Cases belonged to bigger households than controls and tended to share the sleeping room with more people than controls. Presence of cracks in the room’s wall (made of either strong mud, plastered or unplastered cane) tended to increase the risk of VL. Water sources were concordant in the dry and the rainy season (Cohen’s kappa 0.75) and differed between cases and controls, the main source of drinking water being surface water for controls while cases tended to drink more often water from the village water tank. Trees tended to be more frequent in and around cases yards but, after adjustment, only the presence of *Azadirachta indica* and *Acacia nilotica* appeared more likely associated (p<0.05) with an increased risk of VL. Increasing distance between the house yard and the adjacent yard tended to reduce the risk of VL while the presence of a forest at eye range from the house appeared to increase the risk of VL. At night, in both seasons, animals (donkeys, goats, sheep, cattle and dogs) were reported in the majority of participants’ yards. The presence of dogs in the yard at night was associated with an increased risk of VL while the presence of other animals in the yard appeared rather associated with a reduced VL risk.

**Table 2:** Individual level determinants associated (p<0.20) with VL after adjustment: crude and adjusted analysis. Gedaref State, Sudan, 2012–2013.

	Control (N = 801)		Case (N = 198)		Univariate				Adjusted*			
	n	%	n	%	Crude OR	95% CI		LR test	Adjusted OR	95% CI		LR test
**Gender, in <10 years**												
Male	146	51.8	69	66.4	1			0.005	1			<0.001
Female	136	48.2	35	33.7	0.46	0.29	0.74		0.41	0.25	0.68	
**Gender, per 10 years**												
Male	267	51.5	55	58.5	1				1			
Female	251	48.5	39	41.5	0.56	0.4	0.8		0.53	0.36	0.76	
**Age**, per 10 years, among males												
* Median (IQR)*	*14*	*(7–35)*	*8*	*(6–14)*	0.6	0.44	0.83		0.55	0.39	0.77	
**Age**, per 10 years, among females												
* Median (IQR)*	*14*	*(7–30)*	*10*	*(6–24)*	0.73	0.64	0.85		0.7	0.6	0.81	
**Education** *(281 too young for school excluded)*												
Illiterate	126	21.4	18	13.9	1			<0.001	1			<0.001
Primary school	122	20.7	15	11.6	0.86	0.41	1.78		0.7	0.3	1.64	
Secondary school	35	5.9	6	4.6	1.2	0.44	3.25		0.8	0.26	2.49	
Literate via Koranic school	144	24.4	12	9.3	0.58	0.27	1.26		0.66	0.27	1.59	
Currently attending school	162	27.5	78	60.5	3.37	1.92	5.92		2.57	1.17	5.68	
**Occupation**												
No occupation**	586	73.1	172	86.7	1			<0.001	1			0.166
Farming/herding	149	18.6	17	8.7	0.39	0.23	0.66		0.53	0.26	1.1	
Trade /skilled labour	66	8.2	9	4.5	0.46	0.23	0.95		0.54	0.22	1.31	
**History of travel in Gedaref State** (outside Gureisha/Gedaref town)	38	4.7	5	2.5	0.52	0.2	1.34	0.134	0.45	0.17	1.22	0.093
**Bedtime hour** *** *(20 missing)*												
17h to 20h	237	43	112	57.1	3.09	1.37	6.93	<0.001	1.99	0.82	4.82	0.145
21h to 22h	381	48.7	77	39.3	1.88	0.83	4.25		1.46	0.61	3.48	
23h to 2h	65	8.3	7	3.6	1				1			
**Place of stay from sunset to sleep** *** *(50 in bed before/ at sunset excluded)*												
Indoor only	213	27.9	44	23.5	1			0.057	1			0.038
Outdoor	314	41.2	67	35.8	1.07	0.71	1.62		1.17	0.74	1.84	
Both indoor and outdoor	235	30.8	76	40.6	1.55	1.03	2.35		1.74	1.1	2.73	
**Main evening outdoor activities** *** *(1 missing)*												
Stay indoor only	243	30.4	53	26.8	1			<0.001	1			<0.001
Playing	154	19.3	65	32.8	1.94	1.28	2.94		1.71	1.08	2.7	
TV / radio	52	6.5	27	13.6	2.39	1.38	4.15		2.67	1.42	5.03	
Discussing-relaxing	265	33.1	47	23.7	0.82	0.53	1.25		1.02	0.62	1.66	
Cooking/house activities	47	5.9	4	2	0.39	0.14	1.13		0.52	0.17	1.59	
Other activities	39	4.9	2	1	0.24	0.05	1.01		0.31	0.07	1.4	
**Sleep location** ***												
Outside the house yard	23	2.9	15	7.6	2.77	1.42	5.42	0.004	3.33	1.52	7.31	0.003
**Never sleeping in the field/farm** ***	676	84.5	181	91.4	1.95	1.14	3.32	0.009	2.12	1.16	3.91	0.012
**Increase per 1 household member**												
* Mean household size (SD)*	*7*.*7*	*-3*.*3*	*8*.*4*	*-4*	1.06	1.01	1.1	0.012	1.06	1.01	1.12	0.024
**Gender of the head of household**												
Female	38	4.7	5	2.5	0.53	0.2	1.36	0.153	0.46	0.17	1.28	0.113
**Nb sleeping in room** ***, per 1 person												
* Median (IQR)*	*4*	*(3–6)*	*5*	*(3–6)*	1.06	1.01	1.12	0.002	1.04	1	1.09	0.023
**Walls cracked** *(1 missing)*												
No	393	49.1	82	41.4	1			0.013	1			0.079
Yes, but not much	226	28.3	51	25.8	1.05	0.71	1.54		0.9	0.59	1.38	
Yes, many cracks	181	22.6	65	32.8	1.72	1.19	2.49		1.49	0.98	2.25	
**Termites in the room** *(1 Missing)*									1			
No	432	54	118	59.6	1			0.065				0.076
Yes, but not so many	203	25.4	35	17.7	0.63	0.42	0.95		0.62	0.4	0.96	
Yes, many	165	20.6	45	22.7	1	0.68	1.47		1	0.65	1.54	
**Source of drinking water** (in the dry season) *(1 missing)*									1			
Village water tank	162	20.2	73	36.9	1			<0.001				0.064
River/surface water	342	42.7	41	20.7	0.27	0.17	0.41		0.44	0.22	0.86	
Pump, well, water sellers	297	37.1	84	42.4	0.63	0.43	0.91		0.75	0.5	1.14	
**Termite hills in house yard**												
Yes (versus no)	94	11.7	11	5.6	0.44	0.23	0.84	0.007	0.59	0.29	1.17	0.113
**Shelter in the house yard**												
Yes (versus no)	717	89.5	170	85.9	0.71	0.45	1.13	0.155	0.66	0.4	1.1	0.119
**Animal accommodation in the yard**												
Yes (versus no)	558	69.7	128	64.7	0.8	0.57	1.11	0.176	0.78	0.54	1.12	0.183
**Poultry accommodation in the yard**												
Yes (versus no)	605	75.5	131	66.2	0.63	0.45	0.89	0.009	0.75	0.52	1.09	0.135
**Forest/wood at eye range**												
Yes (versus no)	255	31.9	80	40.4	1.44	1.05	1.99	0.026	1.49	1.02	2.17	0.039
***Balanites aegyptica* around the yard**												
Yes (versus no)	694	86.5	186	93.9	2.6	1.37	4.93	0.001	1.6	0.77	3.34	0.195
***Azadirachta indica* around the yard**												
Yes (versus no)	695	87	181	91.4	1.59	0.93	2.73	0.076	1.92	1.06	3.49	0.026
***Acacia nilotica* around the yard**												
Yes (versus no)	348	43.5	125	63.1	2.22	1.61	3.07	<0.001	1.77	1.24	2.55	0.002
***Acacia mellifera* around the yard**												
Yes (versus no)	183	22.9	71	35.9	1.88	1.35	2.63	<0.001	1.31	0.9	1.91	0.158
**Distance to the closest house yard** *(1 missing)*												
Share a common limit	591	73.9	169	85.4	1			0.001	1			0.005
Space in between (<10m)	119	14.9	14	7.1	0.41	0.23	0.74		0.41	0.22	0.76	
More than 10 m	90	11.3	15	7.6	0.58	0.33	1.03		0.62	0.34	1.16	
**Animals in the yard at night** ***												
Yes (*versus* no)	688	86	158	79.8	0.64	0.43	0.96	0.034	0.69	0.44	1.08	0.107
**Sheep in the yard at night** ***												
Yes (*versus* no)	296	37	59	29.8	0.72	0.52	1.01	0.057	0.78	0.54	1.12	0.177
**Dog in the yard at night** ***												
Yes (*versus* no)	55	6.9	21	10.6	1.58	0.93	2.68	0.1	1.92	1.05	3.49	0.037
**Uncovered animal burrows in the yard** *** *(2 don't know)*												
No	325	40.7	75	37.9	1			0.15	1			0.187
Yes, but not many	71	8.9	11	5.6	0.67	0.34	1.33		0.62	0.3	1.28	
Yes, many	403	50.4	112	56.6	1.2	0.87	1.67		1.15	0.8	1.64	
**Fire or smoke as repellent indoor** *** *(3 missing*, *2 don't know)*												
Daily	295	36.9	92	47.2	1			0.027	1			0.084
Frequently / sometimes	193	24.2	43	22.1	0.71	0.48	1.07		0.78	0.5	1.21	
Rarely / never	311	38.9	60	30.8	0.62	0.43	0.89		0.74	0.49	1.11	
**Smoke from *Acacia seyal* as indoor repellent**	434	54.2	132	66.7	1.69	1.22	2.34	0.001	1.47	1.02	2.12	0.038
**Ground nut oil as animal body repellent**	18	2.3	12	6.1	3.38	1.56	7.34	0.003	3.57	1.49	8.58	0.005

LR: Likelihood ratio; VL: visceral leishmaniasis; OR: Odds Ratio; 95%CI: 95% Confidence Interval;

* Adjusted for age, sex, their interaction, and village as random effect;

**Housework, too young, attending school, unemployed.

*** In the rainy season as a proxy of the whole year (trend of association being similar in both seasons though some factors less frequent in the dry season).

The use of smoke from *Acacia seyal* as indoor repellent in the rainy season was associated with an increased risk of VL. Also, repellent for animal (chemical, local tar, smoke from grass, natural oil) was frequently used but only the use of ground nut body oil was associated with an increased risk of VL.

The final multivariate model combining independent individual and household VL determinants is presented in the Table 3.

**Table 3:** Multivariate logistic regression mixed model of individual and household determinants for visceral leishmaniasis, with village as a random effect. Gedaref, Sudan, 2012–2013.

(N = 998)	OR*	95%CI*		p-value*
**Gender,** *in <10 years*				
Male	1			<0.001
Female	0.38	0.23	0.65	
**Gender,** *≥ 10 years*				
Male	1			<0.001
Female	0.51	0.34	0.78	
**Age,** *per 10 years*, *among males*	0.53	0.23	0.65	<0.001
**Age,** *per 10 years*, *among females*	0.71	0.59	0.85	<0.001
**Main evening outdoor activities** **				
Stay indoor only	1			<0.001
Playing	1.77	1.08	2.90	
TV / radio	3.84	1.92	7.68	
Discussing-relaxing	1.44	0.85	2.45	
Cooking/house activities	0.55	0.17	1.83	
Other activities	0.39	0.08	1.97	
**Sleep location** **				
In the house yard	1			0.002
Outside the house yard	3.91	1.70	9.00	
**Never sleeping in the field/farm** **	2.62	1.32	5.17	0.004
**Household size** *(increase of one person)*	1.07	1.01	1.13	0.024
**Source of drinking water** *(dry season)*				
Village water tank	1			0.014
River/surface water	0.36	0.18	0.71	
Pump, well, water sellers	0.63	0.40	0.99	
**Forest at eye range**	1.71	1.14	2.55	0.009
***Acacia nilotica* in yard’s surroundings**	1.72	1.17	2.53	0.006
**Distance to the closest house**				
Share a common limit	1			<0.001
Space in between (<10m)	0.35	0.18	0.67	
More than 10 m	0.51	0.26	1.00	
**Animals in the yard at night** **	0.56	0.34	0.92	0.024
**Dog in the yard at night** **	2.43	1.27	4.69	0.010
**Smoke from *Acacia seyal* as indoor repellent**	1.57	1.06	2.32	0.024
**Ground nut oil as animal body repellent**	4.15	1.65	10.5	0.003

OR: Odds Ratio; 95%CI: 95% Confidence Interval;

*Adjusted for village as a random effect (14% of variance, 95%CI 5–35%);

** During the rainy season; The level of education of the participant was not included in the model due to collinearity with age.

As shown in the Table 4, during the year prior the study, 7.4% of controls compared to 71.7% of cases had at least one household member who suffered from VL (p<0.001) and, among those, cases had more often 2 to 4 household members affected (21.8% versus 10.2%). Before that, VL was reported to have occurred in the households of about 74% of all participants. In the year prior the study, a patient with VL in an adjacent house was reported by 30.8% of the controls and in 42.6% of the cases. Before that period, VL was reported to have occurred in a neighbour of 95.6% of the controls and 90.2% of the cases. Adjusting for individual and household determinants did not affect the size of the associations between VL and previous VL patients in the household or an adjacent house (Table 4). When combining the variables related to VL patients in the household and the adjacent houses in a multivariate model, reporting a VL patient(s) in the household in the previous year remained the only independent VL determinant.

**Table 4:** Patients with visceral leishmaniasis in the household or an adjacent house: crude and adjusted analysis. Gedaref State, Sudan, 2012–2013.

	Control (N = 801)		Cases (N = 198)		Univariate				Adjusted*			
	n	%	n	%	Crude OR	95% CI		LR test	OR*	95% CI*		LR test*
**In the past year**												
**Among household members** **												
No VL	742	92.6	56	28.3	1			<0.001	1			<0.001
≥ 1 VL case but no or unknown rash	44	5.5	117	59.1	35.2	22.7	54.7		38.4**	22.8	64.7	
≥ 1 VL case who developed a rash	15	1.9	25	12.6	22.1	11.0	44.3		20.1**	8.9	45.4	
**Among adjacent house** *(17 missing)*												
No VL	548	69.2	109	57.4	1			0.003	1			0.235
≥ 1 VL case but no or unknown rash	188	23.7	56	29.5	1.50	1.04	2.15		1.05	0.68	1.61	
≥ 1 VL case who developed a rash	56	7.1	25	13.2	2.24	1.34	3.75		1.68	0.93	3.04	
**Before past year**												
**Among household members** *(3 missing)*												
No VL	213	26.6	45	23.1	1			0.360	1			0.705
≥ 1 VL case but no or unknown rash	423	52.8	114	58.5	1.28	0.87	1.87		0.88	0.56	1.4	
≥ 1 VL case who developed a rash	165	20.6	36	18.5	1.03	0.64	1.67		0.79	0.45	1.4	
**Among adjacent households** *(143 missing)*												
No VL	31	4.4	14	8.8	1			0.061	1			0.020
≥ 1 VL case but no or unknown rash	422	60.6	99	62.3	0.52	0.27	1.01		0.39	0.17	0.91	
≥ 1 VL case who developed a rash	244	35.0	46	28.9	0.42	0.21	0.85		0.29	0.12	0.69	

LR: Likelihood ratio; VL: visceral leishmaniasis; OR: Odds Ratio; 95%CI: 95% Confidence Interval;

* Adjusted for age, sex, their interaction, and village as random effect and for all determinants included in the multivariate model presented in the Table 3;

** Only independent VL determinant found when combining the variables related to VL patients in the household and the adjacent houses in a multivariate model.

## Discussion

In this study, living in the proximity of VL patients was the strongest VL risk factor. Three out of four cases reported having a household member sick with VL in the last year compared to less than one out of ten controls. This has also been reported in the Indian subcontinent [13,29] and for asymptomatic VL in Ethiopia [30]. It underlines the importance of studying further the transmission dynamics between patients and their household contacts [31]. However, the role of PKDL as potential reservoir remains unclear. Indeed, reporting one or more household member(s) sick with VL in the past year was associated with a lower OR if the household member(s) also developed a rash compatible with PKDL. This might suggest that individuals with PKDL are less infectious than individuals with active VL but this might also result from misclassification of rashes as being PKDL although these were due to other diseases.

As previously reported, the risk of VL was higher in males and the young age groups [21] and tended to increase with household size which logically increases the number of possible cases in the household [17]. However, this increased VL risk with the household size might have been overestimated due to the sampling procedures that tended to favour controls from smaller households (by selecting only one control per house). However, important overestimation is unlikely because weighting the final multivariate model to account for the number of eligible controls per house did not change the estimation of the OR nor its precision (S2 Table). By contrast [20,21], ethnicity was not associated with VL after adjustment despite the Fallata/Hausa/Zabarma ethnic group being less represented among VL cases. This does not support the hypothesis of a genetic predisposition independent from environmental or behavioural factors. Most ethnic groups settled in this VL endemic area a long time ago possibly reducing the difference in terms of exposure and development of natural immunity.

Also in contrast, there was no evidence for an association between VL and neither the socioeconomic [15,19,32] nor housing factors [15,17]. Cracks in the walls, an important factor in Ethiopia [17], increased the risk of VL in adjusted but not in the multivariate analysis. This supports a predominant role of outdoor biting, as does the association with VL risk and outdoor evening activities (such as watching TV/listening to the radio or playing). This was more obvious during the rainy than the dry season during which most inhabitants remain unprotected outdoor in the evening. This outdoor exposure during the known biting-time and season of the sandflies might explain why bed-nets did not appear protective against clinical VL, adding to the mixed results reported in the literature [15,33]. Innovative preventive measures deserve further consideration. For example, it might be interesting to evaluate the effectiveness of targeted personal protection strategies, such as the use of adapted chemical repellent use or insecticide-impregnated clothing for household members of VL cases or to inhabitants of villages currently experiencing an increase in VL cases.

Although the most frequent soil around the participants’ yards, there was no association with VL and black cotton soil, whose cracks might offer breeding sites to sandflies [34]. The predominance of black cotton soil in the study area might have limited the possibility to find difference between cases and controls and, consequently, an association with VL. Sandy soil in or around the house yard appeared protective in the univariate analysis but not after adjustment. Soil composition is quite homogenous at village level, and in the yard, the soil is often covered with gravels or sand filling the cracks. This might possibly limit sandflies breeding opportunities and might explain sleeping outside the house yard as a VL risk factor. Also, a buffer space between house yards appeared to offer some protection, possibly against transmission from VL cases living in neighbouring yards.

Having a forest, a likely natural focus of sandflies, at eye range from the house yard appeared as a risk factor for VL as well as the presence of *Acacia nilotica* in its immediate surroundings. However, *Balanites aegyptica*, *Acacia seyal* [4,20] and *Azadirachta indica* [21], that have been reported associated with VL, were not in this study after adjustment. Only the use of smoke *of Acacia seyal* as indoor repellent appeared as an independent determinant of VL. During the time of the survey, about a third of the villages investigated were involved in planting *Azadirachta indica* or cutting *Balanites aegyptica and Acacia seyal*. Therefore reverse cause bias cannot be excluded because such activities might have been more intense around the houses of cases. Also, the surrounding vegetation, and microecological conditions influencing sandflies density, might have been different in the farm and the village. Consequently, sleeping in the field or the farm was associated with a decrease in VL risk, while the opposite was found in Ethiopia [17,35].

The association between having a dog in the yard at night and the risk of VL reinforces the role for dogs in sustaining VL transmission in eastern Africa [16,36]. Asymptomatic dogs have been found carriers of *Leishmania* in Sudan [37,38]. The presence of other animals in the yard at night was found protective. In Ethiopia, *P*. *orientalis* were shown to feed mainly on cattle [39] although none of the sandflies caught in that study was infected with *Leishmania*. Presence of animals or their ownership has alternatively been reported either as VL risk factor or protective factor [13,14,17,19,21]. Consequently, it is important to study locally the possible zoonotic transmission (especially with dogs) and to analyse the bloodmeal of sandflies caught in the house yards of the area. The use of ground nut oil as an animal repellent was associated with an increased risk of VL. The use of insecticide for livestock has also been reported as a VL risk factor in Kenya [19]. This suggests that ground nut oil either repel sandflies that consequently feed on another unprotected host, or actually attracts sandflies to the area. This finding needs confirmation but suggest that other animal repellents should be explored as alternative to ground nut oil.

The data were collected through a structured questionnaire by surveyors carefully trained to ensure standardization of its administration. However, a questionnaire might not always capture accurately the variation in population behaviours. Surveyors were not blinded of the participant status and cases were asked to recall the time period before the onset of the case VL symptoms. This might have contributed to inaccuracies and possible recall or observer bias. Also, because numerous variables were investigated and multiple statistical tests done, random findings cannot be excluded. Notably, the association observed between VL and the source of drinking water is unclear and might result from multiplicity of statistical testing or residual confounding due to imperfect adjustment on villages of residence.

Cases were recruited in the Tabarak Allah hospital. Patients with VL that did not attend this health facility were not included and this might have limited the comparability of cases to community control. However these likely represented only a small fraction of all VL cases occurring in the area where even habitants living in decentralised villages tend to present to Tabarak Allah hospital spontaneously [23]. Also, patients from nomadic population or seasonal workers were not included and this might limit the generalizability of the results. Although an important population at risk in some area [15], they represented less than 5% of the primary VL cases in Tabarak Allah during the study period.

Finally, this study compared VL clinical cases to non-infected controls. Consequently, it evaluated potential determinants for both infection and progression towards VL clinical disease but cannot disentangle these two aspects. In order to fully evaluate determinants for infection, we should also have included cases with subclinical infection. However, laboratory methods to identify subclinical infections are imperfect, which would have led to misclassification. Similarly, the duration of positive rK39 in subclinical infection is unknown and it is thus likely that individuals with past subclinical infection have been included as controls, possibly leading to an underestimation of the strength of some associations.

In conclusion, because of the exploratory approach, these results do not provide strong evidence of causality but still, they provide useful suggestions for guiding further intervention studies on VL preventive measures. Control strategies would benefit from further research on VL transmission within house yards and across villages. In the meantime, efforts should target households of patients diagnosed with VL.

## Supporting Information

S1 File Questionnaire in English. (PDF)

S1 Table Univariate analysis of all determinants. Case-control study, Sudan, 2012–13.
(DOC)

S2 Table Multivariate models of individual and household determinants for visceral leishmaniasis (with village as a random effect). (DOC)

S1 Checklist STROBE checklist for case-control studies. (DOC)
