# Supplementary material for: Determinants of Visceral Leishmaniasis: A Case-Control Study in Gedaref State, Sudan
Source: PLoS Negl Trop Dis. 2015 Nov 6;9(11):e0004187. doi: 10.1371/journal.pntd.0004187 (PMC4636291; doi:10.1371/journal.pntd.0004187)
Supplement: S1 File — (PDF) [file pntd.0004187.s001.pdf]

Risk factors of kala-azar, Gedaref

Date of the interview (dd/mm/yyyy): |__|__|/|__|__|/201|__|

Starting time |__|__|h: |__|__| min   Name of the interviewer_______________

Part 1 - Participant information

1	Age	|__|__|	years (write 00 if less than 1 year)
2	Sex	|__|	1. Male / 2. Female
3	Ethnicity/tribe	|__|__|	1.Massalit / 2.Dajo / 3.Fur / 4.Fallata / 5.Barno / 6.Rezegat/ 7. Arab / 8.Beni Aamir / 9. Bargo / 10.Hausa / 11.Zagawa / 12. Tama / 13.Nuba / 14. Salamat / 15. Birgid / 16. Rachid / 17. Zabarma / 18. Bilala / 99. Don't know<br>19. Other, specify ______________________________
	If case of an adult, go to 5. Otherwise, continue.		
4	In case of a child (< 15 years): what is the relationship of the respondent with the child?	|__|	1. Mother/ 2. Father/ 3. Grandparent/ 4. Uncle-/ 5. Sister-brother/ 6. Other, specify:_______________

Part 2 - Household characteristics and socio-economic information

	How many persons are part of the household for each of the following group?	**Male**	**Female**	
5	**<10 years**	|__|__|	|__|__|	Number<br>(including the participant and the persons temporarily absent)<br>99. Do not know
6	**10-19 years**	|__|__|	|__|__|	
7	**20-39 years**	|__|__|	|__|__|	
8	**40 years or more**	|__|__|	|__|__|	
9	What is the education level of the participant?	|__|		0. Too young to go to school./ 1. Illiterate (cannot read/cannot write)/ 2. Uncompleted 1ary school/ 3. Completed 1ary school/ 4. Uncompleted 2ary school/ 5. Completed 2ary school or more/ 6. Literate through Koranic/Khalwa school only / 7. Currently attending school/ 8. Other: specify:_______________ 9. Don't know
10	What is the main occupation of participant?	|__|__|		01. Farmer (own the land, rent the land) / 02. Agricultural waged labour / 03. Herd animals / 04. Unemployed (Jobless or too old to take job)/ 05. Going to school-student / 06. Water selling and trucking / 07. Tea selling and catering / 08. Porter / 09. Merchant / 10. Petty trade/ 11. Skilled labour (carpenters, construction, welders, driver etc)/ 12. Salaried work (Public service, MoH, MSF, others)/ 13. Playing/stay with mother/too young to have a specific occupation/ 14. Take care of the house/ 99. Don't know/ 15. Other, specify:_________
	In case of a child, go to 12. Otherwise, continue.			
11	Is the participant the head of family?<br>If yes, go to 15. Otherwise, continue	|__|		0. No/ 1. Yes
12	What is the sex of the head of family?	|__|		1. Male / 2. Female
13	What is the education level of the head of the family?	|__|		Same code as question 9<br>8. Other: specify:_______________<br>9. Don't know
14	What is the main occupation of the head of the family?	|__|		Same code as question 10<br>15. Other, specify:_________

|__| - |__|__| - |__|__|__| - 1 |__| - |__|__| - |__|__|__|__| - 2

Team - Village _- Waypoint   Team - Village - MSF ID number

	Does the participant' household own:		
15	≥ one tukul/home in this house yard?	|__|	0. No / 1. Yes / 9. Don't know
16	≥ one tukul/home outside this house yard?	|__|	0. No / 1. Yes / 9. Don't know
17	lands/fields?	|__|	0. No / 1. Yes / 9. Don't know
18	a radio?	|__|	0. No / 1. Yes / 9. Don't know
19	a TV?	|__|	0. No / 1. Yes / 9. Don't know
20	a bicycle?	|__|	0. No / 1. Yes / 9. Don't know
21	a motorcycle?	|__|	0. No / 1. Yes / 9. Don't know
22	a car, truck or tractor?	|__|	0. No / 1. Yes / 9. Don't know
23	mobile phone(s) ?	|__|	0. No / 1. Yes / 9. Don't know
24	a donkey cart (karo)	|__|	0. No / 1. Yes / 9. Don't know
25	a generator?	|__|	0. No / 1. Yes / 9. Don't know
26	cereal stock in the tukul/home?	|__|	0. No / 1. Yes / 9. Don't know
27	cereal stock in the house yard?	|__|	0. No / 1. Yes / 9. Don't know
28	cereal stock outside the house yard?	|__|	0. No / 1. Yes / 9. Don't know
29	cattle, goat, sheep or animal for trade?	|__|	0. No / 1. Yes / 9. Don't know
30	***During the dry season*** What is the household main source of drinking water?	|__|	1. Village water tank / 2. Surface water (River or streams)/ 3. Other surface water (stagnant: pond/pool/swamp) / 4. Hand water pump (bore hole)/ 5. Well / 6. Water sellers/ 7. Sabaloga (collected rain water)/ 8. Other, specify ______________________ 9. don't know
31	***During the dry season*** Do the participant him/herself fetch surface water (river/swamps)? (in case of a child: do the child accompany anybody to fetch surface water?)	|__|	1. Daily/ 2. Frequently, often / 3. Sometimes, occasionally/ 4. Rarely/ 5. Never/ 9. Don't know
32	***During rainy season*** What is the household main source of drinking water?	|__|	Same code as above<br>8. Other, specify ________________________
33	***During the rainy season*** Do the participant him/herself fetch water from surface water (river/swamps) ? (in case of a child: do the child accompany anybody to fetch surface water?)	|__|	1. Daily/ 2. Frequently, often / 3. Sometimes, occasionally/ 4. Rarely/ 5. Never/ 9. Don't know

Part 3 – Movement history

34	Since when is the participant living in this village?	|__|	1. Since birth / 2. Since ≥ 1 year, specify: number of years |__|__|/ 3. Since <1 year, specify: number of months |__|__|/ 4. Other: specify _______. / 9. Don't know
35	Did the participant travel or stay outside the village for ≥ 2 weeks over the past year?<br>If no, go to 41. Otherwise, continue.	|__|	0. No / 1. Yes / 9. Don't know
36	how many times?	|__|	9. Don't know
If yes, list the travels ≥2 weeks with **location** (0. In neighbouring village inside Gureisha locality/ 1. Outside Gureisha			

locality but other locality of Gedaref state/ 2. Gedaref town / 3. Outside Gedaref state/ 4. Outside Sudan (specify) / 9. Don't know), **duration** (Number of weeks/ 99 = don't know) and **purpose** (1. Trade/business/professional purpose/ 2. Study/ 3. herding cattle / 4. hospitalised or medical consultation / 5. Farming or harvesting / 6. Visiting relatives/ 7. Other, specify /9. Don't know) (For a young child, if necessary, consider the purpose of the accompanying adult)					
37	Location<br>Travel 1 |__| Specify: _______	if in one of the 24 villages, specify code Otherwise, write '00'<br>|__|__|	Duration (weeks)<br>|__|__|	Purpose<br>|__|	Specify: __________________
38	Travel 2 |__| Specify: _______	|__|__|	|__|__|	|__|	Specify: __________________
39	Travel 3 |__| Specify: _______	|__|__|	|__|__|	|__|	Specify: __________________
40	Travel 4 |__| Specify: _______	|__|__|	|__|__|	|__|	Specify: __________________

Part 4. Night activities and sleeping habits

		During the rainy season	During the dry season	
41	When does the participant usually go to sleep?	|__|	|__|	1. Before sunset/moghrab prayer/ 2. At sunset/moghrab prayer / 3. After sunset/moghrab prayer/ 4. Very variable from one day to another / 5. Not relevant, baby less than one year/ 9.Do not know.
	If the answer is before or at moghrab prayer or if the : go to 47.<br>*In case of a child less than one year old go to 44.*<br>*Otherwise, continue.*			
42	*If possible specify the exact time*<br>*If the participant answers a range, take the middle of it.*	|__|__|h<br>|__|__|m	|__|__|h<br>|__|__|m	99 = don't know
	If the participant gave the exact time, go to 44. Otherwise continue			
43	In average/usually, how many hours from sunset/moghrab prayer to the time the participant go to sleep?	|__|	|__|	0 = less than 1h30 (before Isha prayer) / 1 = 1h30 (at Isha prayer) / 2 = About 2 hours (just after Isha prayer) / 3 = 3 hours / 4 = 4 hours / 5 = 5 hours or midnight/ 6 = 6 hours or more (after midnight) / 7 = Do not know the number of hours but usually before midnight / 8 = Very variable from one day to another/ 9 = don't know
44	Between the sunset and the time (s)he goes to sleep, where does the participant usually stay?	|__|	|__|	1. Indoor (in a place closed with walls (tukul or other) only / 2. Outdoor (open area / 3. Both indoor and outdoor/ 9 = don't know
	If 'indoor only' go to 47. Otherwise, continue.			
45	Between the sunset and the time (s)he goes to sleep, what is his/her main activity when he/she is outdoor (open area)?	|__|	|__|	1. Farming/ 2. Herding animals/ 3. Playing; too young to have specific activities / 4. Watching TV or listening to radio/ 5. Discussing-relaxing / 6. Selling at the market/ 7. Cooking or other house activities / 8. Other, specify: ___________/ 9. Don't know
46	Between the sunset and the time (s)he goes to sleep, where is (s)he usually located when (s)he is outdoor (open area)?	|__|__|	|__|__|	01. House yard / 02. Market / 03. Forest / 04. Fields / 05. Next to the house yard / 06. In the village but not next to the house yard nor in the market / 07. In a neighbouring village/ 08. Near the river / 99. Don't know/ 10. Other, specify ______________________

47	Does the participant usually sleep in this house?<br>*If yes go to question 49. otherwise, continue.*	|__|	|__|	0. No / 1. Yes / 9. Don't know
48	Where does the participant usually sleep?	|__|	|__|	1. In the village but not this house yard/ 2. In a neighbouring village/ 3. Farm/field / 4. Forest/ 5. Other, specify ______________/ 9. Don't know
49	When in this house yard, where does the participant usually sleep?<br>*If the answer is 1, 2 or 3, keep going.*<br>*In case of a child less than one year old go to 53.*<br>*Otherwise, go to 52.*	|__|	|__|	1. In a brick/cement room / 2. In a tukul/ 3. In a "local" room/ 4 Under a shelter / 5. In an open space/ 6. Under a tree/ 7. Variable according to the weather / 8. Other, specify ___________9. Don't know
50	Before sunrise, does the participant usually go outdoor at night before soboh prayer (5h30-5h45)?	|__|	|__|	1. Daily/ 2. Frequently, often / 3. Sometimes, occasionally/ 4. Rarely/ 5. Never/ 9. Don't know
51	Does the participant usually go outdoor (open area) about the time of soboh prayer (early morning)?	|__|	|__|	1. Daily/ 2. Frequently, often / 3. Sometimes, occasionally/ 4. Rarely/ 5. Never/ 9. Don't know
52	At night, where does the participant go for toilet purpose?	|__|	|__|	1. Latrine in the house yard / 2. Latrines outside the house yard (neighbouring house or other place) / 3. In the house yard but no latrines / 4. Outside the house yard but no latrines/ 5. Not relevant, young child/ 9. Don't know
	**Observation : ask the participant (or care taker) to show you the room/tukul where the participant sleeps when (s)he is in this house yard (if several room/tukul, ask for the main one)**			
53	What is the type of walls in the tukul/room where the participant is used to sleep?	|__|		1. Concrete-brick/ 2. „Strong mud" (mixed or with layer of animal dung) with outside grass or plastic sheeting 3. Strong mud only (nothing outside)/ 4. Cane plastered with mud/ 5. Grass/cane/wood unplastered (uncompleted)/ 6. Metal/zinc 7. Other, specify__________/ 9. Don't know
	If the answer is 5 or 6 go to 55. Otherwise, continue.			
54	Are the walls cracked?	|__|		0. No / 1. Yes, many / 2. Yes but not many / 9. Don't know
55	What is the type of roof in the tukul/room he/she is used to sleep?	|__|		1. Grass and wood / 2. Metalic-zinc / 3. Corrugated iron-cement/ 4. Cane/strong straw/ 7 Other, specify____________/ 9. Don't know
56	What is the type of floor in the tukul/room he/she is used to sleep?	|__|		1. Black cotton soil / 2. Earthen covered with gravel / 3. Sand / 4 Sandy soil (azaza)/ 5. Rock, stone and gravel soil / 6. Cemented/ 7. Mixture based from donkeys and cows dung/ 8. Other, specify_________/ 9. Don't know
57	What is the type of windows in the tukul/room he/she is used to sleep?	|__|		1. No windows/ 2. Windows with shutter/ 3. Windows with screen or curtain/ 4. Open, not protected windows/ 9. Don't know
58	Are there termites in the tukul/room he/she is used to sleep?	|__|		0. No / 1. Yes, many / 2. Yes but not many / 9. Don't know

59	On what the participant usually sleeps?	|__|	|__|	1. On the floor/ 2. On a bed/ 3. On a sheet/ 4. On a mat or matress / 9. Don't know
60	Do not ask: according to your opinion, what is the quality of this tukul?	|__|		1. Very good/ 2. Good/ 3. Poor / 4. Very poor
	**Stop observation**			
61	How many persons usually sleep in the same room/tukul?	|__|__|	|__|__|	Number of persons (including participant) / 99. Do not know
62	Is the floor made wet during the dry season to keep the temperature low or to sweep the floor?		|__|	1. Daily/ 2. Frequently, often / 3. Sometimes, occasionally/ 4. Rarely/ 5. Never/ 9. Don't know
63	Is sand or gravels put on the floor inside the tukul during the rainy season?	|__|		1. Daily/ 2. Frequently, often / 3. Sometimes, occasionally/ 4. Rarely/ 5. Never/ 9. Don't know
64	Was the room/tukul sprayed inside in the last year?	|__|		0. No / 1. Yes, by the household/ 2. Yes, by the MoH or other organisation/ 9. Don't know
65	Does the participant usually sleep covered?<br>*If no go to 67. Otherwise, continue.*	|__|	|__|	1. Covered / 2. Not covered / 3. variable according to the weather/ 9. Don't know
66	*If covered, with what?*	|__|	|__|	1. a sheet / 2. a blanket / 3. Sheet or blanket according to the weather / 4. Other, specify ______ / 9. Don't know
67	Does the participant sleep under a mosquito net *(before hospitalisation for cases)*?<br>*If no go to 71. Otherwise,* continue.	|__|	|__|	1. Yes / 2. No, never / 9. Don't know
68	How frequently does (s)he sleep under the mosquito net?	|__|	|__|	1. Daily/ 2. Frequently, often / 3. Sometimes, occasionally/ 4. Rarely/ 9. Don't know
69	Is the current mosquito net impregnated *(not for cases)*? **(check by observation)**	|__|		0. No / 1. Yes / 9. Don't know
70	Is it in good condition *(not for cases)*? **(check by observation)**	|__|		0. Bad condition / 1. Yes, good condition / 2. Slightly damaged / 9. Don't know
71	Are there usually animals at night in the room/tukul where (s)he is used to sleep?<br>*If no go to 73. Otherwise,* continue.	|__|	|__|	0. No / 1. Yes/ 9. Don't know
72	*What type and how many*	|__|__|Dogs<br>|__|__|Cattle<br>|__|__|Goat<br>|__|__|Sheep	|__|__|Dogs<br>|__|__|Cattle<br>|__|__|Goat<br>|__|__|Sheep	*Number*<br>*99= do not know*
73	Are there usually animals at night in this house yard? (including animals located in animal accommodation)<br>*If no go to 75. Otherwise,* continue.	|__|	|__|	0. No / 1. Yes/ 9. Don't know
74	*What type and how many (count all whether or not they are in the cattle shed)*	|__|__|Dogs<br>|__|__|Donky<br>|__|__|Cattle<br>|__|__|Camel<br>|__|__|Horse<br>|__|__|Goat<br>|__|__|Sheep	|__|__|Dogs<br>|__|__|Donky<br>|__|__|Cattle<br>|__|__|Camel<br>|__|__|Horse<br>|__|__|Goat<br>|__|__|Sheep	*Number*<br>*99= do not know*

75	Are there usually animals at night in the adjacent house yard/immediate surroundings where he/she is used to sleep?<br>*If no go to 77. Otherwise,* continue.	|__|	|__|	0. No / 1. Yes/ 9. Don't know
76	*What type and how many*	|__|__|Dogs<br>|__|__|Donky<br>|__|__|Cattle<br>|__|__|Camel<br>|__|__|Horse<br>|__|__|Goat<br>|__|__|Sheep	|__|__|Dogs<br>|__|__|Donky<br>|__|__|Cattle<br>|__|__|Camel<br>|__|__|Horse<br>|__|__|Goat<br>|__|__|Sheep	*Number*<br>*88 = yes but do not know the number*<br>*99= do not know*
77	Are there rats/rodents in the room/tukul where he/she is used to sleep?	|__|	|__|	0. No / 1. Yes / 9. Don't know
78	Are there animals burrows in the room/tukul where he/she is used to sleep?	|__|	|__|	0. No / 1. Yes, many / 2. Yes but not many / 9. Don't know
79	Are there animals burrows (uncovered) in the house yard where he/she is used to sleep?	|__|	|__|	0. No / 1. Yes, many/ 2. Yes but not many / 9. Don't know
80	Are there usually bodies of water in the house yard? (if several, report the biggest one)	|__|	|__|	0. No / 1. Yes, 'local sewage' / 2. Yes, stagnant rain water/ / 3 Other, specify: ________ 9. Don't know
81	Are there usually bodies of water in the immediate surroundings of the house yard?	|__|	|__|	0. No / 1. Yes, pond/ 2. Yes, river, stream / 3. Yes, stagnant rain water / 4. Other, specify: ________<br>9. Don't know
82	Does the participant sometimes sleep in the field/farm?<br>*If not, go to 86. Otherwise, continue.*	|__|	|__|	1. Yes / 2. No, never / 9. Don't know
83	How many nights over the season?	|__|__|	|__|__|	Number of nights<br>99. Do not know
84	When in the field, where does the participant usually sleep?	|__|	|__|	1. In a brick/cement room / 2. Inside a tukul / 3. In a "local" room/ 4 Under a shelter / 5. In an open space/ 6. Under a tree/ 7. Variable according to the weather / 8. Other, specify ___________9. Don't know
85	When in the field, does the participant sleep under a mosquito net *(before hospitalisation for cases)*?	|__|	|__|	1. Daily/ 2. Frequently, often / 3. Sometimes, occasionally/ 4. Rarely/ 5. Never/ 9. Don't know
85b	Does the participant sometimes herd animals? (In case child, does s(he) accompany other person herding animals)?<br>*If not, go to 90. Otherwise, continue.*	|__|	|__|	1. Yes / 2. No / 9. Don't know
86	Does the participant sometimes sleep outside the village for herding cattle?<br>*If not, go to 90. Otherwise, continue.*	|__|	|__|	1. Yes / 2. No, never / 9. Don't know
87	How many nights over the season?	|__|__|	|__|__|	Number of nights<br>99. Do not know
88	When herding cattle, where does the participant usually sleep?	|__|	|__|	0. Tent / 1. In a brick/cement room / 2. Inside a tukul / 3. In a "local" room/ 4 Under a shelter / 5. In an open space/ 6. Under a tree/ 7. Variable according

				to the weather / 8. Other, specify ___________9. Don't know
89	When herding cattle, does the participant sleep under a mosquito net *(before hospitalisation for cases)*?	|__|	|__|	1. Daily/ 2. Frequently, often / 3. Sometimes, occasionally/ 4. Rarely/ 5. Never/ 9. Don't know
90	Does the participant usually have naps during day time?	|__|	|__|	1. Daily/ 2. Frequently, often / 3. Sometimes, occasionally/ 4. Rarely/ 5. Never / 9. Don't know
	If never, go to 100. Otherwise, continue.			
91	Is he/she having the nap under trees?	|__|	|__|	1. Daily/ 2. Frequently, often / 3. Sometimes, occasionally/ 4. Rarely/ 5. Never / 9. Don't know
	If never, go to 100. Otherwise, continue.			
	Type of trees (***cfr pictures)***			
92	Taleh (Acacia seyal)	|__|	|__|	0. No / 1. Yes / 9. Don't know
93	Lalob or higleeg (Balanites aegyptiaca)	|__|	|__|	0. No / 1. Yes / 9. Don't know
94	Neem (azadirachta indica)	|__|	|__|	0. No / 1. Yes / 9. Don't know
95	Hashab (Acacia senegal)	|__|	|__|	0. No / 1. Yes / 9. Don't know
96	Kiter (Acacia Mullifera)	|__|	|__|	0. No / 1. Yes / 9. Don't know
97	Sonot/Garad	|__|	|__|	0. No / 1. Yes / 9. Don't know
98	Sidir	|__|	|__|	0. No / 1. Yes / 9. Don't know
99	Other, specify____________________	|__|	|__|	0. No / 1. Yes / 9. Don't know
100	Does the participant have activities in the wood/forest (playing, cutting wood, hunting)? (In case child, does s(he) accompany other person in the forest)?	|__|	|__|	1. Yes / 2. No, never / 9. Don't know
	If no go to 102 otherwise, continue			
101	How frequently?	|__|	|__|	1. Daily/ 2. Frequently, often / 3. Sometimes, occasionally/ 4. Rarely/ 9. Don't know
102	Is the participant using chemical insect repellents for himself?	|__|	|__|	1. Daily/ 2. Frequently, often / 3. Sometimes, occasionally/ 4. Rarely/ 5. Never/ 9. Don't know
103	Is the participant using natural oil or other products for his/her body?	|__|	|__|	1. Daily/ 2. Frequently, often / 3. Sometimes, occasionally/ 4. Rarely/ 5. Never/ 9. Don't know
	If never go to 105 otherwise, continue			
104	Specify the type of oil	|__|	|__|	1. Simsim/sesame oil / 2. Neem oil / 3. Oil from Lalob / 4. Ground nut/beans oil / 5. Petroleum product (gazoline) / 6. Other Specify: _______________/ 9. Do not know
105	Is participant using fire or smoking woods as insect repellent in the tukul/room?	|__|	|__|	1. Daily/ 2. Frequently, often / 3. Sometimes, occasionally/ 4. Rarely/ 5. Never/ 9. Don't know
	If never go to 107 otherwise, continue			
106	Specify the type of wood/material			1. Taleh / 2. Neem / 3. Lalob / 4 Other, specify: _____________________/ 9. Don't know
	If the participant's household has no animals (see 73 and 75), go to 111, otherwise, continue			

107	Does the household use insect repellent for the body of animals?	|__|	|__|	1. Daily/ 2. Frequently, often / 3. Sometimes, occasionally/ 4. Rarely/ 5. Never/ 9. Don't know
	If no go to 109 otherwise, continue			
108	Specify the type of repellent	|__|	|__|	1. Chemical / 2. Local 'Tar' (Quotran)/ 3. Other, specify: ____________________/ 9. Don't know
109	Is the participant using other insect repellents for animals?	|__|	|__|	1. Daily/ 2. Frequently, often / 3. Sometimes, occasionally/ 4. Rarely/ 5. Never/ 9. Don't know
	If no go to 111 otherwise, continue			
110	The type of repellent	|__|	|__|	1. Smoke from grass / 2. Smoke from wood/ 3. Smoke from grass and wood/ 4. Other, specify: ________________/ 9. Don't know

Part 5 - Personal medical history and knowledge of VL

111	Does the participant currently take a long term (at least one month) medical treatment prescribed by a medical person (do not consider treatment related to Kala Azar)?	|__|	0. No / 1. Yes / 9. Don't know	
	If no, go to 113 *(144 for case)*. Otherwise continue			
112	Specify the type of treatment: ______________________			
113	Did the participant (or his/her caretaker) know Kala azar before the interview *(do not ask this question to cases)*?	|__|	0. No / 1. Yes	
	If no, go to 122. Otherwise, continue.			
		In the past year *(in the year before the participant was sick for cases)*	Before the past year *(more than one year before the participant was sick for cases)*	
114	Has any of the household member of the participant been sick with KA?	|__|	|__|	0. No/ 1. Yes / 9. Don't know
	If no, go to 119. Otherwise, continue.			
115	How many persons?	|__|__|	|__|__|	99. Do not know *(For case, do not count the participant!)*
116	How many of them received a medical treatment (in health center or hospital)?	|__|__|	|__|__|	99. Do not know *(For case, do not count the participant!)*
117	How many of them developed a rash (PKDL - show picture)?	|__|__|	|__|__|	99. Do not know *(For case, do not count the participant!)*
	If none, go to 119. Otherwise, continue			
118	Was (any of) the(se) rash(es) still present in the last year *(in the year before the participant was sick for cases)*?	|__|	|__|	0. No/ 1. Yes / 9. Don't know
119	Has any inhabitant of the adjacent/bordering houses been sick with KA?	|__|	|__|	0. No/ 1. Yes / 9. Don't know

	If no, go to 122. Otherwise, continue.			
120	How many persons?	|__|__|	|__|__|	99. Do not know
121	How many of them received a medical treatment (in health center or hospital)?	|__|__|	|__|__|	99. Do not know
122	Has any inhabitant of the adjacent/bordering houses been presenting such a rash (PKDL - show picture)?	|__|	|__|	0. No/ 1. Yes / 9. Don't know
	If no, go to 125. Otherwise, continue.			
123	How many persons?	|__|__|	|__|__|	99. Do not know
124	Was (any of) the(se) rash(es) still present in the last year *(in the year before the participant was sick for cases)*?	|__|	|__|	0. No/ 1. Yes / 9. Don't know
	For the following questions: note the answer spontaneously given! Do not suggest anything! Do not ask the question if the person did not know KA before (see question 133)			
	What can be done to prevent KA?			
125	Use a mosquito net	|__|	0. Not reported by the participant (or his/her caretaker)/ 1. Spontaneously reported by the participant (or his/her caretaker)	
126	Use insect repellent for human	|__|	(same code as above)	
127	Use insect repellent for animals	|__|	(same code as above)	
128	Spray home (inside)	|__|	(same code as above)	
129	Cut the trees	|__|	(same code as above)	
130	Covering/filling crack in the soil/walls	|__|	(same code as above)	
131	Killing dogs	|__|	(same code as above)	
132	Fighting rodents	|__|	(same code as above)	
133	Cleaning in and around the house	|__|	(same code as above)	
134	Stay inside the tukul, not going out at night	|__|	(same code as above)	
135	Cover your body	|__|	(same code as above)	
136	Other specify ______________________________	|__|	(same code as above)	

Part 6 – House observation

	In the house yard, count and report the number of		Number
137	Brick cement room	|__|	
138	Tukuls	|__|	
139	Local room	|__|	
140	Shelters	|__|	
141	Animal's accomodation (no poultry, no birds)	|__|	
142	Poultry accommodation	|__|	
143	Is there "farming/crops" in the house yard?	|__|	0. No / 1. Yes

144	What is the type of soil in the house yard?	|__|	1. Black cotton soil / 2. Earthen covered with gravel / 3. Sand / 4 Sandy soil (azaza)/ 5. Rock, stone and gravel soil / 6. Cemented/ 7. Mixture based from donkeys and cows dung/ 8. Other, specify_________/ 9. Don't know
145	What is the type of soil in the 10 meters around the house yard?	|__|	1. Black cotton soil / 2. Earthen covered with gravel / 3. Sand / 4 Sandy soil (azaza)/ 5. Rock, stone and gravel soil / 6. Cemented/ 7. Mixture based from donkeys and cows dung/ 8. Other, specify_________/ 9. Don't know
	Count the number of following trees:	*In the house yard*	*At in the neighbouring house yard and immediate surroundings of the house yard*
146	Taleh (Acacia seyal)	|__|__|	|__|__|
147	Lalob or higleeg (Balanites aegyptiaca)	|__|__|	|__|__|
148	Neem (azadirachta indica)	|__|__|	|__|__|
149	Hashab (Acacia senegal	|__|__|	|__|__|
150	Kiter (Acacia Mullifera)	|__|__|	|__|__|
151	Sonot/ Garad	|__|__|	|__|__|
152	Sidir	|__|__|	|__|__|
153	Other trees? Specify ____________	|__|__|	|__|__|
154	Count the number of termite hills (write 00 if none):	|__|__|	|__|__|
155	Did you cut trees in the house yard over the past year?	|__|	0. No / 1. Yes / 9. Don't know
	If no, go to 156. Otherwise continue		
158	How many?	|__|__|	99: Do not know
156	Is there a forest/wood at eye range from the household?	|__|	0. No / 1. Yes, small forest/ 2. Yes or dense forest / 9. Don't know
157	How far is the closest house?	|__|	1. Share a common limit (wall or other – no space between)/ 2. Space in between (less than 10 meters)/ 3. More than 10 meters but less than 100 meters / 4. More than 100 meters / 9 Do no know.

Thank the participant for his/her participation and give him/her a mosquito net

Ending time |__|__|h : |__|__| min

Comments :____

____

____

____

____

____

|__| - |__|__| - |__|__|__|__| - 1 |__| - |__|__| - |__|__|__|__| - 2

Team - Village _- Waypoint Team - Village - MSF ID number
