# Supplementary material for: Determinants of Visceral Leishmaniasis: A Case-Control Study in Gedaref State, Sudan
Source: PLoS Negl Trop Dis. 2015 Nov 6;9(11):e0004187. doi: 10.1371/journal.pntd.0004187 (PMC4636291; doi:10.1371/journal.pntd.0004187)
Supplement: S1 Table — Case-control study, Sudan, 2012–13. (DOC) [file pntd.0004187.s002.doc]

**Univariate analysis of all determinants. Case-control study, Sudan, 2012-1**3.

|  | | | | | | | |  | | | | | | | | | | | | | | | | | | | | | | | | | | | | | | | | | | | | | | | | | | | | | | | | | | | | | Control (N=801) | | | | | | | | | | | | | | | | | | | | | | | | | | | | | | | | | | | | | | | | | | | | | | | | | Case (N=198) | | | | | | | | | | | | | | | | | | | | | | | | | | | | | | | | | | | | | p-value *(Pearson chi², Fisher’s exact, t-test, Wilcoxon)* | | | | | | | | | | | | | | | | | | |
| --- | --- | --- | --- | --- | --- | --- | --- | --- | --- | --- | --- | --- | --- | --- | --- | --- | --- | --- | --- | --- | --- | --- | --- | --- | --- | --- | --- | --- | --- | --- | --- | --- | --- | --- | --- | --- | --- | --- | --- | --- | --- | --- | --- | --- | --- | --- | --- | --- | --- | --- | --- | --- | --- | --- | --- | --- | --- | --- | --- | --- | --- | --- | --- | --- | --- | --- | --- | --- | --- | --- | --- | --- | --- | --- | --- | --- | --- | --- | --- | --- | --- | --- | --- | --- | --- | --- | --- | --- | --- | --- | --- | --- | --- | --- | --- | --- | --- | --- | --- | --- | --- | --- | --- | --- | --- | --- | --- | --- | --- | --- | --- | --- | --- | --- | --- | --- | --- | --- | --- | --- | --- | --- | --- | --- | --- | --- | --- | --- | --- | --- | --- | --- | --- | --- | --- | --- | --- | --- | --- | --- | --- | --- | --- | --- | --- | --- | --- | --- | --- | --- | --- | --- | --- | --- | --- | --- | --- | --- | --- | --- | --- | --- | --- | --- | --- |
|  | | | | | | | |  | | | | | | | | | | | | | | | | | | | | | | | | | | | | | | | | | | | | | | | | | | | | | | | | | | | | | n | | | | | | | | | | | | | | | | | | | | | | | | % | | | | | | | | | | | | | | | | | | | | | | | | | n | | | | | | | | | | | | | | | | | | | % | | | | | | | | | | | | | | | | | |  |  |  |  |  |  |  |  |  |  |  |  |  |  |  |  |  |  |  |
| Age, sex and village of residence (variables included in all thematic multivariate model) | | | | | | | | | | | | | | | | | | | | | | | | | | | | | | | | | | | | | | | | | | | | | | | | | | | | | | | | | | | | | | | | | | | | | | | | | | | | | | | | | | | | | | | | | | | | | | | | | | | | | | | | | | | | | | | | | | | | | | | | | | | | | | | | | | | | | | | | | | | | | | | | | | | | | | | | | | | | | | | | | | | | | |
| **Age** | | | | | | | | 0 to 9 years | | | | | | | | | | | | | | | | | | | | | | | | | | | | | | | | | | | | | | | | | | | | | | | | | | | | | 283 | | | | | | | | | | | | | | | | | | | | | | | | 35.3 | | | | | | | | | | | | | | | | | | | | | | | | | 104 | | | | | | | | | | | | | | | | | | | 52.5 | | | | | | | | | | | | | | | | | | <0.001 | | | | | | | | | | | | | | | | | | |
|  | | | | | | | | 10 to 19 years | | | | | | | | | | | | | | | | | | | | | | | | | | | | | | | | | | | | | | | | | | | | | | | | | | | | | 196 | | | | | | | | | | | | | | | | | | | | | | | | 24.5 | | | | | | | | | | | | | | | | | | | | | | | | | 52 | | | | | | | | | | | | | | | | | | | 26.3 | | | | | | | | | | | | | | | | | |  | | | | | | | | | | | | | | | | | | |
|  | | | | | | | | 20 to 39 years | | | | | | | | | | | | | | | | | | | | | | | | | | | | | | | | | | | | | | | | | | | | | | | | | | | | | 187 | | | | | | | | | | | | | | | | | | | | | | | | 23.4 | | | | | | | | | | | | | | | | | | | | | | | | | 24 | | | | | | | | | | | | | | | | | | | 12.1 | | | | | | | | | | | | | | | | | |  | | | | | | | | | | | | | | | | | | |
|  | | | | | | | | 40 years or more | | | | | | | | | | | | | | | | | | | | | | | | | | | | | | | | | | | | | | | | | | | | | | | | | | | | | 135 | | | | | | | | | | | | | | | | | | | | | | | | 16.9 | | | | | | | | | | | | | | | | | | | | | | | | | 18 | | | | | | | | | | | | | | | | | | | 9.1 | | | | | | | | | | | | | | | | | |  | | | | | | | | | | | | | | | | | | |
| **Sex** | | | | | | | | Female | | | | | | | | | | | | | | | | | | | | | | | | | | | | | | | | | | | | | | | | | | | | | | | | | | | | | 387 | | | | | | | | | | | | | | | | | | | | | | | | 48.3 | | | | | | | | | | | | | | | | | | | | | | | | | 74 | | | | | | | | | | | | | | | | | | | 37.4 | | | | | | | | | | | | | | | | | | 0.006 | | | | | | | | | | | | | | | | | | |
|  | | | | | | | | Male | | | | | | | | | | | | | | | | | | | | | | | | | | | | | | | | | | | | | | | | | | | | | | | | | | | | | 414 | | | | | | | | | | | | | | | | | | | | | | | | 51.7 | | | | | | | | | | | | | | | | | | | | | | | | | 124 | | | | | | | | | | | | | | | | | | | 62.6 | | | | | | | | | | | | | | | | | |  | | | | | | | | | | | | | | | | | | |
| **Village** | | | | | | | | Berber Al-Fugera | | | | | | | | | | | | | | | | | | | | | | | | | | | | | | | | | | | | | | | | | | | | | | | | | | | | | 87 | | | | | | | | | | | | | | | | | | | | | | | | 10.9 | | | | | | | | | | | | | | | | | | | | | | | | | 59 | | | | | | | | | | | | | | | | | | | 29.8 | | | | | | | | | | | | | | | | | | <0.001 | | | | | | | | | | | | | | | | | | |
|  | | | | | | | | Tabarak Allah | | | | | | | | | | | | | | | | | | | | | | | | | | | | | | | | | | | | | | | | | | | | | | | | | | | | | 84 | | | | | | | | | | | | | | | | | | | | | | | | 10.5 | | | | | | | | | | | | | | | | | | | | | | | | | 43 | | | | | | | | | | | | | | | | | | | 21.7 | | | | | | | | | | | | | | | | | |  | | | | | | | | | | | | | | | | | | |
|  | | | | | | | | Khuor Zaraf | | | | | | | | | | | | | | | | | | | | | | | | | | | | | | | | | | | | | | | | | | | | | | | | | | | | | 78 | | | | | | | | | | | | | | | | | | | | | | | | 9.7 | | | | | | | | | | | | | | | | | | | | | | | | | 7 | | | | | | | | | | | | | | | | | | | 3.5 | | | | | | | | | | | | | | | | | |  | | | | | | | | | | | | | | | | | | |
|  | | | | | | | | Jebel Ghana | | | | | | | | | | | | | | | | | | | | | | | | | | | | | | | | | | | | | | | | | | | | | | | | | | | | | 63 | | | | | | | | | | | | | | | | | | | | | | | | 7.9 | | | | | | | | | | | | | | | | | | | | | | | | | 24 | | | | | | | | | | | | | | | | | | | 12.1 | | | | | | | | | | | | | | | | | |  | | | | | | | | | | | | | | | | | | |
|  | | | | | | | | Al-asira | | | | | | | | | | | | | | | | | | | | | | | | | | | | | | | | | | | | | | | | | | | | | | | | | | | | | 62 | | | | | | | | | | | | | | | | | | | | | | | | 7.7 | | | | | | | | | | | | | | | | | | | | | | | | | 10 | | | | | | | | | | | | | | | | | | | 5.1 | | | | | | | | | | | | | | | | | |  | | | | | | | | | | | | | | | | | | |
|  | | | | | | | | Um Gzaz | | | | | | | | | | | | | | | | | | | | | | | | | | | | | | | | | | | | | | | | | | | | | | | | | | | | | 54 | | | | | | | | | | | | | | | | | | | | | | | | 6.7 | | | | | | | | | | | | | | | | | | | | | | | | | 1 | | | | | | | | | | | | | | | | | | | 0.5 | | | | | | | | | | | | | | | | | |  | | | | | | | | | | | | | | | | | | |
|  | | | | | | | | Birkat Norein | | | | | | | | | | | | | | | | | | | | | | | | | | | | | | | | | | | | | | | | | | | | | | | | | | | | | 52 | | | | | | | | | | | | | | | | | | | | | | | | 6.5 | | | | | | | | | | | | | | | | | | | | | | | | | 11 | | | | | | | | | | | | | | | | | | | 5.6 | | | | | | | | | | | | | | | | | |  | | | | | | | | | | | | | | | | | | |
|  | | | | | | | | Mashra Al Forsan | | | | | | | | | | | | | | | | | | | | | | | | | | | | | | | | | | | | | | | | | | | | | | | | | | | | | 51 | | | | | | | | | | | | | | | | | | | | | | | | 6.4 | | | | | | | | | | | | | | | | | | | | | | | | | 8 | | | | | | | | | | | | | | | | | | | 4.0 | | | | | | | | | | | | | | | | | |  | | | | | | | | | | | | | | | | | | |
|  | | | | | | | | Wad Arood | | | | | | | | | | | | | | | | | | | | | | | | | | | | | | | | | | | | | | | | | | | | | | | | | | | | | 41 | | | | | | | | | | | | | | | | | | | | | | | | 5.1 | | | | | | | | | | | | | | | | | | | | | | | | | 0 | | | | | | | | | | | | | | | | | | | 0.0 | | | | | | | | | | | | | | | | | |  | | | | | | | | | | | | | | | | | | |
|  | | | | | | | | Saref Al-ardeba | | | | | | | | | | | | | | | | | | | | | | | | | | | | | | | | | | | | | | | | | | | | | | | | | | | | | 36 | | | | | | | | | | | | | | | | | | | | | | | | 4.5 | | | | | | | | | | | | | | | | | | | | | | | | | 9 | | | | | | | | | | | | | | | | | | | 4.6 | | | | | | | | | | | | | | | | | |  | | | | | | | | | | | | | | | | | | |
|  | | | | | | | | Muodereia & El-jakka | | | | | | | | | | | | | | | | | | | | | | | | | | | | | | | | | | | | | | | | | | | | | | | | | | | | | 32 | | | | | | | | | | | | | | | | | | | | | | | | 4.0 | | | | | | | | | | | | | | | | | | | | | | | | | 7 | | | | | | | | | | | | | | | | | | | 3.5 | | | | | | | | | | | | | | | | | |  | | | | | | | | | | | | | | | | | | |
|  | | | | | | | | Wad Koly | | | | | | | | | | | | | | | | | | | | | | | | | | | | | | | | | | | | | | | | | | | | | | | | | | | | | 30 | | | | | | | | | | | | | | | | | | | | | | | | 3.8 | | | | | | | | | | | | | | | | | | | | | | | | | 7 | | | | | | | | | | | | | | | | | | | 3.5 | | | | | | | | | | | | | | | | | |  | | | | | | | | | | | | | | | | | | |
|  | | | | | | | | Um Tokel | | | | | | | | | | | | | | | | | | | | | | | | | | | | | | | | | | | | | | | | | | | | | | | | | | | | | 25 | | | | | | | | | | | | | | | | | | | | | | | | 3.1 | | | | | | | | | | | | | | | | | | | | | | | | | 1 | | | | | | | | | | | | | | | | | | | 0.5 | | | | | | | | | | | | | | | | | |  | | | | | | | | | | | | | | | | | | |
|  | | | | | | | | Teibar | | | | | | | | | | | | | | | | | | | | | | | | | | | | | | | | | | | | | | | | | | | | | | | | | | | | | 21 | | | | | | | | | | | | | | | | | | | | | | | | 2.6 | | | | | | | | | | | | | | | | | | | | | | | | | 4 | | | | | | | | | | | | | | | | | | | 2.0 | | | | | | | | | | | | | | | | | |  | | | | | | | | | | | | | | | | | | |
|  | | | | | | | | Areeda South | | | | | | | | | | | | | | | | | | | | | | | | | | | | | | | | | | | | | | | | | | | | | | | | | | | | | 18 | | | | | | | | | | | | | | | | | | | | | | | | 2.2 | | | | | | | | | | | | | | | | | | | | | | | | | 5 | | | | | | | | | | | | | | | | | | | 2.5 | | | | | | | | | | | | | | | | | |  | | | | | | | | | | | | | | | | | | |
|  | | | | | | | | Wad Harry | | | | | | | | | | | | | | | | | | | | | | | | | | | | | | | | | | | | | | | | | | | | | | | | | | | | | 16 | | | | | | | | | | | | | | | | | | | | | | | | 2.0 | | | | | | | | | | | | | | | | | | | | | | | | | 0 | | | | | | | | | | | | | | | | | | | 0.0 | | | | | | | | | | | | | | | | | |  | | | | | | | | | | | | | | | | | | |
|  | | | | | | | | Jazeer Al-Dabi | | | | | | | | | | | | | | | | | | | | | | | | | | | | | | | | | | | | | | | | | | | | | | | | | | | | | 13 | | | | | | | | | | | | | | | | | | | | | | | | 1.6 | | | | | | | | | | | | | | | | | | | | | | | | | 0 | | | | | | | | | | | | | | | | | | | 0.0 | | | | | | | | | | | | | | | | | |  | | | | | | | | | | | | | | | | | | |
|  | | | | | | | | Debekar | | | | | | | | | | | | | | | | | | | | | | | | | | | | | | | | | | | | | | | | | | | | | | | | | | | | | 9 | | | | | | | | | | | | | | | | | | | | | | | | 1.1 | | | | | | | | | | | | | | | | | | | | | | | | | 0 | | | | | | | | | | | | | | | | | | | 0.0 | | | | | | | | | | | | | | | | | |  | | | | | | | | | | | | | | | | | | |
|  | | | | | | | | Al-Lyah | | | | | | | | | | | | | | | | | | | | | | | | | | | | | | | | | | | | | | | | | | | | | | | | | | | | | 9 | | | | | | | | | | | | | | | | | | | | | | | | 1.1 | | | | | | | | | | | | | | | | | | | | | | | | | 1 | | | | | | | | | | | | | | | | | | | 0.5 | | | | | | | | | | | | | | | | | |  | | | | | | | | | | | | | | | | | | |
|  | | | | | | | | Marpata | | | | | | | | | | | | | | | | | | | | | | | | | | | | | | | | | | | | | | | | | | | | | | | | | | | | | 9 | | | | | | | | | | | | | | | | | | | | | | | | 1.1 | | | | | | | | | | | | | | | | | | | | | | | | | 0 | | | | | | | | | | | | | | | | | | | 0.0 | | | | | | | | | | | | | | | | | |  | | | | | | | | | | | | | | | | | | |
|  | | | | | | | | Areeda North | | | | | | | | | | | | | | | | | | | | | | | | | | | | | | | | | | | | | | | | | | | | | | | | | | | | | 4 | | | | | | | | | | | | | | | | | | | | | | | | 0.5 | | | | | | | | | | | | | | | | | | | | | | | | | 0 | | | | | | | | | | | | | | | | | | | 0.0 | | | | | | | | | | | | | | | | | |  | | | | | | | | | | | | | | | | | | |
|  | | | | | | | | Dagendiba | | | | | | | | | | | | | | | | | | | | | | | | | | | | | | | | | | | | | | | | | | | | | | | | | | | | | 3 | | | | | | | | | | | | | | | | | | | | | | | | 0.4 | | | | | | | | | | | | | | | | | | | | | | | | | 0 | | | | | | | | | | | | | | | | | | | 0.0 | | | | | | | | | | | | | | | | | |  | | | | | | | | | | | | | | | | | | |
|  | | | | | | | | Moshra Elnile | | | | | | | | | | | | | | | | | | | | | | | | | | | | | | | | | | | | | | | | | | | | | | | | | | | | | 2 | | | | | | | | | | | | | | | | | | | | | | | | 0.3 | | | | | | | | | | | | | | | | | | | | | | | | | 1 | | | | | | | | | | | | | | | | | | | 0.5 | | | | | | | | | | | | | | | | | |  | | | | | | | | | | | | | | | | | | |
|  | | | | | | | | Al maderdaoma | | | | | | | | | | | | | | | | | | | | | | | | | | | | | | | | | | | | | | | | | | | | | | | | | | | | | 2 | | | | | | | | | | | | | | | | | | | | | | | | 0.3 | | | | | | | | | | | | | | | | | | | | | | | | | 0 | | | | | | | | | | | | | | | | | | | 0.0 | | | | | | | | | | | | | | | | | |  | | | | | | | | | | | | | | | | | | |
| Thematic section (individual level): Demographic characteristics | | | | | | | | | | | | | | | | | | | | | | | | | | | | | | | | | | | | | | | | | | | | | | | | | | | | | | | | | | | | | | | | | | | | | | | | | | | | | | | | | | | | | | | | | | | | | | | | | | | | | | | | | | | | | | | | | | | | | | | | | | | | | | | | | | | | | | | | | | | | | | | | | | | | | | | | | | | | | | | | | | | | | |
| **Ethnicity** | | | | | | | | Massalit | | | | | | | | | | | | | | | | | | | | | | | | | | | | | | | | | | | | | | | | | | | | | | | | | | | | | 263 | | | | | | | | | | | | | | | | | | | | | | | | 34.8 | | | | | | | | | | | | | | | | | | | | | | | | | 57 | | | | | | | | | | | | | | | | | | | 31.0 | | | | | | | | | | | | | | | | | | Not available (NA) | | | | | | | | | | | | | | | | | | |
| (60 missing) | | | | | | | | Zabarma | | | | | | | | | | | | | | | | | | | | | | | | | | | | | | | | | | | | | | | | | | | | | | | | | | | | | 74 | | | | | | | | | | | | | | | | | | | | | | | | 9.8 | | | | | | | | | | | | | | | | | | | | | | | | | 14 | | | | | | | | | | | | | | | | | | | 7.6 | | | | | | | | | | | | | | | | | |  | | | | | | | | | | | | | | | | | | |
|  | | | | | | | | Fallata | | | | | | | | | | | | | | | | | | | | | | | | | | | | | | | | | | | | | | | | | | | | | | | | | | | | | 65 | | | | | | | | | | | | | | | | | | | | | | | | 8.6 | | | | | | | | | | | | | | | | | | | | | | | | | 3 | | | | | | | | | | | | | | | | | | | 1.6 | | | | | | | | | | | | | | | | | |  | | | | | | | | | | | | | | | | | | |
|  | | | | | | | | Hausa | | | | | | | | | | | | | | | | | | | | | | | | | | | | | | | | | | | | | | | | | | | | | | | | | | | | | 59 | | | | | | | | | | | | | | | | | | | | | | | | 7.8 | | | | | | | | | | | | | | | | | | | | | | | | | 7 | | | | | | | | | | | | | | | | | | | 3.8 | | | | | | | | | | | | | | | | | |  | | | | | | | | | | | | | | | | | | |
|  | | | | | | | | Tama | | | | | | | | | | | | | | | | | | | | | | | | | | | | | | | | | | | | | | | | | | | | | | | | | | | | | 52 | | | | | | | | | | | | | | | | | | | | | | | | 6.9 | | | | | | | | | | | | | | | | | | | | | | | | | 37 | | | | | | | | | | | | | | | | | | | 20.1 | | | | | | | | | | | | | | | | | |  | | | | | | | | | | | | | | | | | | |
|  | | | | | | | | Gemir | | | | | | | | | | | | | | | | | | | | | | | | | | | | | | | | | | | | | | | | | | | | | | | | | | | | | 36 | | | | | | | | | | | | | | | | | | | | | | | | 4.8 | | | | | | | | | | | | | | | | | | | | | | | | | 11 | | | | | | | | | | | | | | | | | | | 6.0 | | | | | | | | | | | | | | | | | |  | | | | | | | | | | | | | | | | | | |
|  | | | | | | | | Arnga | | | | | | | | | | | | | | | | | | | | | | | | | | | | | | | | | | | | | | | | | | | | | | | | | | | | | 27 | | | | | | | | | | | | | | | | | | | | | | | | 3.6 | | | | | | | | | | | | | | | | | | | | | | | | | 8 | | | | | | | | | | | | | | | | | | | 4.4 | | | | | | | | | | | | | | | | | |  | | | | | | | | | | | | | | | | | | |
|  | | | | | | | | Messeria (Sudanese, Arabs) | | | | | | | | | | | | | | | | | | | | | | | | | | | | | | | | | | | | | | | | | | | | | | | | | | | | | 20 | | | | | | | | | | | | | | | | | | | | | | | | 2.7 | | | | | | | | | | | | | | | | | | | | | | | | | 4 | | | | | | | | | | | | | | | | | | | 2.2 | | | | | | | | | | | | | | | | | |  | | | | | | | | | | | | | | | | | | |
|  | | | | | | | | Rachid (Sudanese, Arabs) | | | | | | | | | | | | | | | | | | | | | | | | | | | | | | | | | | | | | | | | | | | | | | | | | | | | | 19 | | | | | | | | | | | | | | | | | | | | | | | | 2.5 | | | | | | | | | | | | | | | | | | | | | | | | | 2 | | | | | | | | | | | | | | | | | | | 1.1 | | | | | | | | | | | | | | | | | |  | | | | | | | | | | | | | | | | | | |
|  | | | | | | | | Salamat (Sudanese, Arabs) | | | | | | | | | | | | | | | | | | | | | | | | | | | | | | | | | | | | | | | | | | | | | | | | | | | | | 10 | | | | | | | | | | | | | | | | | | | | | | | | 1.3 | | | | | | | | | | | | | | | | | | | | | | | | | 1 | | | | | | | | | | | | | | | | | | | 0.5 | | | | | | | | | | | | | | | | | |  | | | | | | | | | | | | | | | | | | |
|  | | | | | | | | Rezegat (Sudanese, Arabs) | | | | | | | | | | | | | | | | | | | | | | | | | | | | | | | | | | | | | | | | | | | | | | | | | | | | | 8 | | | | | | | | | | | | | | | | | | | | | | | | 1.1 | | | | | | | | | | | | | | | | | | | | | | | | | 2 | | | | | | | | | | | | | | | | | | | 1.1 | | | | | | | | | | | | | | | | | |  | | | | | | | | | | | | | | | | | | |
|  | | | | | | | | Beni Halba (Sudanese, Arabs) | | | | | | | | | | | | | | | | | | | | | | | | | | | | | | | | | | | | | | | | | | | | | | | | | | | | | 6 | | | | | | | | | | | | | | | | | | | | | | | | 0.8 | | | | | | | | | | | | | | | | | | | | | | | | | 0 | | | | | | | | | | | | | | | | | | | 0.0 | | | | | | | | | | | | | | | | | |  | | | | | | | | | | | | | | | | | | |
|  | | | | | | | | Other Sudanese, Arabs | | | | | | | | | | | | | | | | | | | | | | | | | | | | | | | | | | | | | | | | | | | | | | | | | | | | | 18 | | | | | | | | | | | | | | | | | | | | | | | | 2.4 | | | | | | | | | | | | | | | | | | | | | | | | | 5 | | | | | | | | | | | | | | | | | | | 2.7 | | | | | | | | | | | | | | | | | |  | | | | | | | | | | | | | | | | | | |
|  | | | | | | | | Dajo (Sudanese, Non Arabs) | | | | | | | | | | | | | | | | | | | | | | | | | | | | | | | | | | | | | | | | | | | | | | | | | | | | | 12 | | | | | | | | | | | | | | | | | | | | | | | | 1.6 | | | | | | | | | | | | | | | | | | | | | | | | | 6 | | | | | | | | | | | | | | | | | | | 3.3 | | | | | | | | | | | | | | | | | |  | | | | | | | | | | | | | | | | | | |
|  | | | | | | | | Barno (Sudanese, Non Arabs) | | | | | | | | | | | | | | | | | | | | | | | | | | | | | | | | | | | | | | | | | | | | | | | | | | | | | 11 | | | | | | | | | | | | | | | | | | | | | | | | 1.5 | | | | | | | | | | | | | | | | | | | | | | | | | 1 | | | | | | | | | | | | | | | | | | | 0.5 | | | | | | | | | | | | | | | | | |  | | | | | | | | | | | | | | | | | | |
|  | | | | | | | | Zagawa (Sudanese, Non Arabs) | | | | | | | | | | | | | | | | | | | | | | | | | | | | | | | | | | | | | | | | | | | | | | | | | | | | | 11 | | | | | | | | | | | | | | | | | | | | | | | | 1.5 | | | | | | | | | | | | | | | | | | | | | | | | | 5 | | | | | | | | | | | | | | | | | | | 2.7 | | | | | | | | | | | | | | | | | |  | | | | | | | | | | | | | | | | | | |
|  | | | | | | | | Birgid (Sudanese, Non Arabs) | | | | | | | | | | | | | | | | | | | | | | | | | | | | | | | | | | | | | | | | | | | | | | | | | | | | | 9 | | | | | | | | | | | | | | | | | | | | | | | | 1.2 | | | | | | | | | | | | | | | | | | | | | | | | | 7 | | | | | | | | | | | | | | | | | | | 3.8 | | | | | | | | | | | | | | | | | |  | | | | | | | | | | | | | | | | | | |
|  | | | | | | | | Fur (Sudanese, Non Arabs) | | | | | | | | | | | | | | | | | | | | | | | | | | | | | | | | | | | | | | | | | | | | | | | | | | | | | 6 | | | | | | | | | | | | | | | | | | | | | | | | 0.8 | | | | | | | | | | | | | | | | | | | | | | | | | 1 | | | | | | | | | | | | | | | | | | | 0.5 | | | | | | | | | | | | | | | | | |  | | | | | | | | | | | | | | | | | | |
|  | | | | | | | | Other Sudanese, Non Arabs | | | | | | | | | | | | | | | | | | | | | | | | | | | | | | | | | | | | | | | | | | | | | | | | | | | | | 3 | | | | | | | | | | | | | | | | | | | | | | | | 0.4 | | | | | | | | | | | | | | | | | | | | | | | | | 2 | | | | | | | | | | | | | | | | | | | 1.1 | | | | | | | | | | | | | | | | | |  | | | | | | | | | | | | | | | | | | |
|  | | | | | | | | Other | | | | | | | | | | | | | | | | | | | | | | | | | | | | | | | | | | | | | | | | | | | | | | | | | | | | | 46 | | | | | | | | | | | | | | | | | | | | | | | | 6.1 | | | | | | | | | | | | | | | | | | | | | | | | | 11 | | | | | | | | | | | | | | | | | | | 8.2 | | | | | | | | | | | | | | | | | |  | | | | | | | | | | | | | | | | | | |
| **Education** (281 too young to go to school, 2 missing) | | | | | | | | | | | | | | | | | | | | | | | | | | | | | | | | | | | | | | | | | | | | | | | | | | | | | | | | | | | | |  | | | | | | | | | | | | | | | | | | | | | | | |  | | | | | | | | | | | | | | | | | | | | | | | | |  | | | | | | | | | | | | | | | | | | |  | | | | | | | | | | | | | | | | | | <0.001 | | | | | | | | | | | | | | | | | | |
|  | | | | | | | | Illiterate | | | | | | | | | | | | | | | | | | | | | | | | | | | | | | | | | | | | | | | | | | | | | | | | | | | | | 126 | | | | | | | | | | | | | | | | | | | | | | | | 21.4 | | | | | | | | | | | | | | | | | | | | | | | | | 18 | | | | | | | | | | | | | | | | | | | 13.9 | | | | | | | | | | | | | | | | | |  | | | | | | | | | | | | | | | | | | |
|  | | | | | | | | Primary school | | | | | | | | | | | | | | | | | | | | | | | | | | | | | | | | | | | | | | | | | | | | | | | | | | | | | 122 | | | | | | | | | | | | | | | | | | | | | | | | 20.7 | | | | | | | | | | | | | | | | | | | | | | | | | 15 | | | | | | | | | | | | | | | | | | | 11.6 | | | | | | | | | | | | | | | | | |  | | | | | | | | | | | | | | | | | | |
|  | | | | | | | | Secondary school | | | | | | | | | | | | | | | | | | | | | | | | | | | | | | | | | | | | | | | | | | | | | | | | | | | | | 35 | | | | | | | | | | | | | | | | | | | | | | | | 5.9 | | | | | | | | | | | | | | | | | | | | | | | | | 6 | | | | | | | | | | | | | | | | | | | 4.6 | | | | | | | | | | | | | | | | | |  | | | | | | | | | | | | | | | | | | |
|  | | | | | | | | Literate through Koranic school | | | | | | | | | | | | | | | | | | | | | | | | | | | | | | | | | | | | | | | | | | | | | | | | | | | | | 144 | | | | | | | | | | | | | | | | | | | | | | | | 24.4 | | | | | | | | | | | | | | | | | | | | | | | | | 12 | | | | | | | | | | | | | | | | | | | 9.3 | | | | | | | | | | | | | | | | | |  | | | | | | | | | | | | | | | | | | |
|  | | | | | | | | Currently attending school | | | | | | | | | | | | | | | | | | | | | | | | | | | | | | | | | | | | | | | | | | | | | | | | | | | | | 162 | | | | | | | | | | | | | | | | | | | | | | | | 27.5 | | | | | | | | | | | | | | | | | | | | | | | | | 78 | | | | | | | | | | | | | | | | | | | 60.5 | | | | | | | | | | | | | | | | | |  | | | | | | | | | | | | | | | | | | |
| **Occupation** | | | | | | | | Too young | | | | | | | | | | | | | | | | | | | | | | | | | | | | | | | | | | | | | | | | | | | | | | | | | | | | | 228 | | | | | | | | | | | | | | | | | | | | | | | | 28.5 | | | | | | | | | | | | | | | | | | | | | | | | | 73 | | | | | | | | | | | | | | | | | | | 36.9 | | | | | | | | | | | | | | | | | | <0.001 | | | | | | | | | | | | | | | | | | |
|  | | | | | | | | Going to school-student | | | | | | | | | | | | | | | | | | | | | | | | | | | | | | | | | | | | | | | | | | | | | | | | | | | | | 180 | | | | | | | | | | | | | | | | | | | | | | | | 22.5 | | | | | | | | | | | | | | | | | | | | | | | | | 79 | | | | | | | | | | | | | | | | | | | 39.9 | | | | | | | | | | | | | | | | | |  | | | | | | | | | | | | | | | | | | |
|  | | | | | | | | Housework | | | | | | | | | | | | | | | | | | | | | | | | | | | | | | | | | | | | | | | | | | | | | | | | | | | | | 150 | | | | | | | | | | | | | | | | | | | | | | | | 18.7 | | | | | | | | | | | | | | | | | | | | | | | | | 17 | | | | | | | | | | | | | | | | | | | 8.6 | | | | | | | | | | | | | | | | | |  | | | | | | | | | | | | | | | | | | |
|  | | | | | | | | Unemployed | | | | | | | | | | | | | | | | | | | | | | | | | | | | | | | | | | | | | | | | | | | | | | | | | | | | | 28 | | | | | | | | | | | | | | | | | | | | | | | | 3.5 | | | | | | | | | | | | | | | | | | | | | | | | | 3 | | | | | | | | | | | | | | | | | | | 1.5 | | | | | | | | | | | | | | | | | |  | | | | | | | | | | | | | | | | | | |
|  | | | | | | | | Farmer | | | | | | | | | | | | | | | | | | | | | | | | | | | | | | | | | | | | | | | | | | | | | | | | | | | | | 138 | | | | | | | | | | | | | | | | | | | | | | | | 17.2 | | | | | | | | | | | | | | | | | | | | | | | | | 13 | | | | | | | | | | | | | | | | | | | 6.6 | | | | | | | | | | | | | | | | | |  | | | | | | | | | | | | | | | | | | |
|  | | | | | | | | Herd animals | | | | | | | | | | | | | | | | | | | | | | | | | | | | | | | | | | | | | | | | | | | | | | | | | | | | | 10 | | | | | | | | | | | | | | | | | | | | | | | | 1.3 | | | | | | | | | | | | | | | | | | | | | | | | | 0 | | | | | | | | | | | | | | | | | | | 0.0 | | | | | | | | | | | | | | | | | |  | | | | | | | | | | | | | | | | | | |
|  | | | | | | | | Agricultural waged labour | | | | | | | | | | | | | | | | | | | | | | | | | | | | | | | | | | | | | | | | | | | | | | | | | | | | | 1 | | | | | | | | | | | | | | | | | | | | | | | | 0.1 | | | | | | | | | | | | | | | | | | | | | | | | | 4 | | | | | | | | | | | | | | | | | | | 2.0 | | | | | | | | | | | | | | | | | |  | | | | | | | | | | | | | | | | | | |
|  | | | | | | | | Skilled labour | | | | | | | | | | | | | | | | | | | | | | | | | | | | | | | | | | | | | | | | | | | | | | | | | | | | | 34 | | | | | | | | | | | | | | | | | | | | | | | | 4.2 | | | | | | | | | | | | | | | | | | | | | | | | | 5 | | | | | | | | | | | | | | | | | | | 2.5 | | | | | | | | | | | | | | | | | |  | | | | | | | | | | | | | | | | | | |
|  | | | | | | | | Petty trade | | | | | | | | | | | | | | | | | | | | | | | | | | | | | | | | | | | | | | | | | | | | | | | | | | | | | 19 | | | | | | | | | | | | | | | | | | | | | | | | 2.4 | | | | | | | | | | | | | | | | | | | | | | | | | 0 | | | | | | | | | | | | | | | | | | | 0.0 | | | | | | | | | | | | | | | | | |  | | | | | | | | | | | | | | | | | | |
|  | | | | | | | | Salaried work | | | | | | | | | | | | | | | | | | | | | | | | | | | | | | | | | | | | | | | | | | | | | | | | | | | | | 6 | | | | | | | | | | | | | | | | | | | | | | | | 0.7 | | | | | | | | | | | | | | | | | | | | | | | | | 2 | | | | | | | | | | | | | | | | | | | 1.0 | | | | | | | | | | | | | | | | | |  | | | | | | | | | | | | | | | | | | |
|  | | | | | | | | Merchant | | | | | | | | | | | | | | | | | | | | | | | | | | | | | | | | | | | | | | | | | | | | | | | | | | | | | 4 | | | | | | | | | | | | | | | | | | | | | | | | 0.5 | | | | | | | | | | | | | | | | | | | | | | | | | 1 | | | | | | | | | | | | | | | | | | | 0.5 | | | | | | | | | | | | | | | | | |  | | | | | | | | | | | | | | | | | | |
|  | | | | | | | | Tea selling and cater | | | | | | | | | | | | | | | | | | | | | | | | | | | | | | | | | | | | | | | | | | | | | | | | | | | | | 1 | | | | | | | | | | | | | | | | | | | | | | | | 0.1 | | | | | | | | | | | | | | | | | | | | | | | | | 0 | | | | | | | | | | | | | | | | | | | 0.0 | | | | | | | | | | | | | | | | | |  | | | | | | | | | | | | | | | | | | |
|  | | | | | | | | Porter | | | | | | | | | | | | | | | | | | | | | | | | | | | | | | | | | | | | | | | | | | | | | | | | | | | | | 1 | | | | | | | | | | | | | | | | | | | | | | | | 0.1 | | | | | | | | | | | | | | | | | | | | | | | | | 0 | | | | | | | | | | | | | | | | | | | 0.0 | | | | | | | | | | | | | | | | | |  | | | | | | | | | | | | | | | | | | |
|  | | | | | | | | Water selling and trucking | | | | | | | | | | | | | | | | | | | | | | | | | | | | | | | | | | | | | | | | | | | | | | | | | | | | | 1 | | | | | | | | | | | | | | | | | | | | | | | | 0.1 | | | | | | | | | | | | | | | | | | | | | | | | | 1 | | | | | | | | | | | | | | | | | | | 0.5 | | | | | | | | | | | | | | | | | |  | | | | | | | | | | | | | | | | | | |
| Under long term medical treatment (2 missing) | | | | | | | | | | | | | | | | | | | | | | | | | | | | | | | | | | | | | | | | | | | | | | | | | | | | | | | | | | | | | | 34 | | | | | | | | | | | | | | | | | | | | | | | | 4.3 | | | | | | | | | | | | | | | | | | | | | | | | 8 | | | | | | | | | | | | | | | | | | | | | | 4.0 | | | | | | | | | | | | | | | | | | 0.893 | | | | | | | | | | | | | | | |
| Thematic section (individual level): Travel history | | | | | | | | | | | | | | | | | | | | | | | | | | | | | | | | | | | | | | | | | | | | | | | | | | | | | | | | | | | | | | | | | | | | | | | | | | | | | | | | | | | | | | | | | | | | | | | | | | | | | | | | | | | | | | | | | | | | | | | | | | | | | | | | | | | | | | | | | | | | | | | | | | | | | | | | | | | | | | | | | | | | | |
| Living in village since birth | | | | | | | | | | | | | | | | | | | | | | | | | | | | | | | | | | | | | | | | | | | | | | | | | | | | | | | | | | | | | 583 | | | | | | | | | | | | | | | | | | | | | | | | | 72.8 | | | | | | | | | | | | | | | | | | | | | | | | 165 | | | | | | | | | | | | | | | | | | | | | 83.3 | | | | | | | | | | | | | | | | | | 0.002 | | | | | | | | | | | | | | | | |
| Years lived outside village [Median (IQR)] (N=251) | | | | | | | | | | | | | | | | | | | | | | | | | | | | | | | | | | | | | | | | | | | | | | | | | | | | | | | | | | | | | 15 | | | | | | | | | | | | | | | | | | | | | | | | | (8, 23) | | | | | | | | | | | | | | | | | | | | | | | | 12 | | | | | | | | | | | | | | | | | | | | | (6, 20) | | | | | | | | | | | | | | | | | | 0.218 | | | | | | | | | | | | | | | | |
| Travel outside village for ≥ 2 weeks (past year) | | | | | | | | | | | | | | | | | | | | | | | | | | | | | | | | | | | | | | | | | | | | | | | | | | | | | | | | | | | | | 190 | | | | | | | | | | | | | | | | | | | | | | | | | 23.7 | | | | | | | | | | | | | | | | | | | | | | | | 37 | | | | | | | | | | | | | | | | | | | | | 18.7 | | | | | | | | | | | | | | | | | | 0.130 | | | | | | | | | | | | | | | | |
| Number of times outside village for >= 2 weeks | | | | | | | | | | | | | | | | | | | | | | | | | | | | | | | | | | | | | | | | | | | | | | | | | | | | | | | | | | | | | | | | | | | | | | | | | | | | | | | | | | | | | | | | | | | | | | | | | | | | | | | | | | | | | |  | | | | | | | | | | | | | | | | | | | | |  | | | | | | | | | | | | | | | | | | 0.082 | | | | | | | | | | | | | | | | |
|  | | | | | | | | 0 | | | | | | | | | | | | | | | | | | | | | | | | | | | | | | | | | | | | | | | | | | | | | | | | | | | | | | 611 | | | | | | | | | | | | | | | | | | | | | | | | 76.3 | | | | | | | | | | | | | | | | | | | | | | | | 161 | | | | | | | | | | | | | | | | | | | | | 81.3 | | | | | | | | | | | | | | | | | |  | | | | | | | | | | | | | | | | |
|  | | | | | | | | 1 | | | | | | | | | | | | | | | | | | | | | | | | | | | | | | | | | | | | | | | | | | | | | | | | | | | | | | 172 | | | | | | | | | | | | | | | | | | | | | | | | 21.5 | | | | | | | | | | | | | | | | | | | | | | | | 30 | | | | | | | | | | | | | | | | | | | | | 15.2 | | | | | | | | | | | | | | | | | |  | | | | | | | | | | | | | | | | |
|  | | | | | | | | 2 | | | | | | | | | | | | | | | | | | | | | | | | | | | | | | | | | | | | | | | | | | | | | | | | | | | | | | 16 | | | | | | | | | | | | | | | | | | | | | | | | 2.0 | | | | | | | | | | | | | | | | | | | | | | | | 5 | | | | | | | | | | | | | | | | | | | | | 2.5 | | | | | | | | | | | | | | | | | |  | | | | | | | | | | | | | | | | |
|  | | | | | | | | 3 | | | | | | | | | | | | | | | | | | | | | | | | | | | | | | | | | | | | | | | | | | | | | | | | | | | | | | 2 | | | | | | | | | | | | | | | | | | | | | | | | 0.3 | | | | | | | | | | | | | | | | | | | | | | | | 2 | | | | | | | | | | | | | | | | | | | | | 1.0 | | | | | | | | | | | | | | | | | |  | | | | | | | | | | | | | | | | |
| Median number of weeks outside village (IQR) | | | | | | | | | | | | | | | | | | | | | | | | | | | | | | | | | | | | | | | | | | | | | | | | | | | | | | | | | | | | | | 4 | | | | | | | | | | | | | | | | | | | | | | | | (3, 8) | | | | | | | | | | | | | | | | | | | | | | | | 6 | | | | | | | | | | | | | | | | | | | | | (4, 10) | | | | | | | | | | | | | | | | | | 0.153 | | | | | | | | | | | | | | | | |
| **Travel destination** Travel inside Gureisha locality | | | | | | | | | | | | | | | | | | | | | | | | | | | | | | | | | | | | | | | | | | | | | | | | | | | | | | | | | | | | | | 21 | | | | | | | | | | | | | | | | | | | | | | | | 2.6 | | | | | | | | | | | | | | | | | | | | | | | | 3 | | | | | | | | | | | | | | | | | | | | | 1.5 | | | | | | | | | | | | | | | | | | 0.448 | | | | | | | | | | | | | | | | |
| Travel to Gedaref town | | | | | | | | | | | | | | | | | | | | | | | | | | | | | | | | | | | | | | | | | | | | | | | | | | | | | | | | | | | | | | 28 | | | | | | | | | | | | | | | | | | | | | | | | 3.5 | | | | | | | | | | | | | | | | | | | | | | | | 3 | | | | | | | | | | | | | | | | | | | | | 1.5 | | | | | | | | | | | | | | | | | | 0.150 | | | | | | | | | | | | | | | | |
| Travel in Gedaref State  (outside Gureisha & Gedaref town) | | | | | | | | | | | | | | | | | | | | | | | | | | | | | | | | | | | | | | | | | | | | | | | | | | | | | | | | | | | | | | 38 | | | | | | | | | | | | | | | | | | | | | | | | 4.7 | | | | | | | | | | | | | | | | | | | | | | | | 5 | | | | | | | | | | | | | | | | | | | | | 2.5 | | | | | | | | | | | | | | | | | | 0.168 | | | | | | | | | | | | | | | | |
| Travel outside Gedaref State | | | | | | | | | | | | | | | | | | | | | | | | | | | | | | | | | | | | | | | | | | | | | | | | | | | | | | | | | | | | | | 110 | | | | | | | | | | | | | | | | | | | | | | | | 13.7 | | | | | | | | | | | | | | | | | | | | | | | | 27 | | | | | | | | | | | | | | | | | | | | | 13.6 | | | | | | | | | | | | | | | | | | 0.972 | | | | | | | | | | | | | | | | |
| **Travel purpose** | | | | | | | | | | | | | | | | | | | | | | | | | | | | | | | | | | | | | | | | | | | | | | | | | | | | | | | | | | | | | |  | | | | | | | | | | | | | | | | | | | | | | | |  | | | | | | | | | | | | | | | | | | | | | | | |  | | | | | | | | | | | | | | | | | | | | |  | | | | | | | | | | | | | | | | | |  | | | | | | | | | | | | | | | | |
| Visiting relatives | | | | | | | | | | | | | | | | | | | | | | | | | | | | | | | | | | | | | | | | | | | | | | | | | | | | | | | | | | | | | | 106 | | | | | | | | | | | | | | | | | | | | | | | | 13.2 | | | | | | | | | | | | | | | | | | | | | | | | 20 | | | | | | | | | | | | | | | | | | | | | 10.1 | | | | | | | | | | | | | | | | | | 0.235 | | | | | | | | | | | | | | | | |
| Trade/business/professional purpose | | | | | | | | | | | | | | | | | | | | | | | | | | | | | | | | | | | | | | | | | | | | | | | | | | | | | | | | | | | | | | 61 | | | | | | | | | | | | | | | | | | | | | | | | 7.6 | | | | | | | | | | | | | | | | | | | | | | | | 12 | | | | | | | | | | | | | | | | | | | | | 6.1 | | | | | | | | | | | | | | | | | | 0.452 | | | | | | | | | | | | | | | | |
| Hospitalised or medical consultation | | | | | | | | | | | | | | | | | | | | | | | | | | | | | | | | | | | | | | | | | | | | | | | | | | | | | | | | | | | | | | 22 | | | | | | | | | | | | | | | | | | | | | | | | 2.8 | | | | | | | | | | | | | | | | | | | | | | | | 2 | | | | | | | | | | | | | | | | | | | | | 1.0 | | | | | | | | | | | | | | | | | | 0.198 | | | | | | | | | | | | | | | | |
| Study | | | | | | | | | | | | | | | | | | | | | | | |  | | | | | | | | | | | | | | | | | | | | | | | | | | | | | | | | | | | | | | 4 | | | | | | | | | | | | | | | | | | | | | | | | 0.5 | | | | | | | | | | | | | | | | | | | | | | | | 2 | | | | | | | | | | | | | | | | | | | | | 1.0 | | | | | | | | | | | | | | | | | | 0.340 | | | | | | | | | | | | | | | | |
| Others (Herding cattle, holidays, condolences) | | | | | | | | | | | | | | | | | | | | | | | | | | | | | | | | | | | | | | | | | | | | | | | | | | | | | | | | | | | | | | 2 | | | | | | | | | | | | | | | | | | | | | | | | 0.2 | | | | | | | | | | | | | | | | | | | | | | | | 1 | | | | | | | | | | | | | | | | | | | | | 0.5 | | | | | | | | | | | | | | | | | | 0.557 | | | | | | | | | | | | | | | | |
| Thematic section (individual level): Activities and sleeping habits in the rainy season | | | | | | | | | | | | | | | | | | | | | | | | | | | | | | | | | | | | | | | | | | | | | | | | | | | | | | | | | | | | | | | | | | | | | | | | | | | | | | | | | | | | | | | | | | | | | | | | | | | | | | | | | | | | | | | | | | | | | | | | | | | | | | | | | | | | | | | | | | | | | | | | | | | | | | | | | | | | | | | | | | | | | |
| **Fetch surface water** | | | | | | | | | | | | | | | | | | | | | | | | | | | | | | Daily | | | | | | | | | | | | | | | | | | | | | | | | | | | | | | | | | | | | | 111 | | | | | | | | | | | | | | | | | | | | | | | | | | 13.9 | | | | | | | | | | | | | | | | | | | | 18 | | | | | | | | | | | | | | | | | | | | | | | 9.1 | | | | | | | | | | | | | | | | | | 0.304 | | | | | | | | | | | |
|  | | | | | | | | | | | | | | | | | | | | | | | | | | | | | | Frequently | | | | | | | | | | | | | | | | | | | | | | | | | | | | | | | | | | | | | 45 | | | | | | | | | | | | | | | | | | | | | | | | | | 5.6 | | | | | | | | | | | | | | | | | | | | 8 | | | | | | | | | | | | | | | | | | | | | | | 4.0 | | | | | | | | | | | | | | | | | |  | | | | | | | | | | | |
|  | | | | | | | | | | | | | | | | | | | | | | | | | | | | | | Sometimes | | | | | | | | | | | | | | | | | | | | | | | | | | | | | | | | | | | | | 101 | | | | | | | | | | | | | | | | | | | | | | | | | | 12.6 | | | | | | | | | | | | | | | | | | | | 26 | | | | | | | | | | | | | | | | | | | | | | | 13.1 | | | | | | | | | | | | | | | | | |  | | | | | | | | | | | |
|  | | | | | | | | | | | | | | | | | | | | | | | | | | | | | | Rarely | | | | | | | | | | | | | | | | | | | | | | | | | | | | | | | | | | | | | 14 | | | | | | | | | | | | | | | | | | | | | | | | | | 1.8 | | | | | | | | | | | | | | | | | | | | 2 | | | | | | | | | | | | | | | | | | | | | | | 1.0 | | | | | | | | | | | | | | | | | |  | | | | | | | | | | | |
|  | | | | | | | | | | | | | | | | | | | | | | | | | | | | | | Never | | | | | | | | | | | | | | | | | | | | | | | | | | | | | | | | | | | | | 530 | | | | | | | | | | | | | | | | | | | | | | | | | | 66.2 | | | | | | | | | | | | | | | | | | | | 144 | | | | | | | | | | | | | | | | | | | | | | | 72.7 | | | | | | | | | | | | | | | | | |  | | | | | | | | | | | |
| **Activities in the forest** | | | | | | | | | | | | | | | | | | | | | | | | | | | | | | | | | | | Never | | | | | | | | | | | | | | | | | | | | | | | | | | | | | | | | 633 | | | | | | | | | | | | | | | | | | | | | | | | | | 79.4 | | | | | | | | | | | | | | | | | | | | 170 | | | | | | | | | | | | | | | | | | | | | | | 85.9 | | | | | | | | | | | | | | | | | | 0.038 | | | | | | | | | | | |
| **How frequently** | | | | | | | | | | | | | | | | | | | | | | | | | | | | | | | | Daily | | | | | | | | | | | | | | | | | | | | | | | | | | | | | | | | | | | 20 | | | | | | | | | | | | | | | | | | | | | | | | | | 12.1 | | | | | | | | | | | | | | | | | | | | 1 | | | | | | | | | | | | | | | | | | | | | | | 3.6 | | | | | | | | | | | | | | | | | | 0.097 | | | | | | | | | | | |
| (n=193) | | | | | | | | | | | | | | | | | | | | | | | | | | | | | | | | Frequently | | | | | | | | | | | | | | | | | | | | | | | | | | | | | | | | | | | 37 | | | | | | | | | | | | | | | | | | | | | | | | | | 22.4 | | | | | | | | | | | | | | | | | | | | 7 | | | | | | | | | | | | | | | | | | | | | | | 25.0 | | | | | | | | | | | | | | | | | |  | | | | | | | | | | | |
|  | | | | | | | | | | | | | | | | | | | | | | | | | | | | | | | | Sometimes | | | | | | | | | | | | | | | | | | | | | | | | | | | | | | | | | | | 89 | | | | | | | | | | | | | | | | | | | | | | | | | | 53.9 | | | | | | | | | | | | | | | | | | | | 20 | | | | | | | | | | | | | | | | | | | | | | | 71.4 | | | | | | | | | | | | | | | | | |  | | | | | | | | | | | |
|  | | | | | | | | | | | | | | | | | | | | | | | | | | | | | | | | Rarely | | | | | | | | | | | | | | | | | | | | | | | | | | | | | | | | | | | 19 | | | | | | | | | | | | | | | | | | | | | | | | | | 11.5 | | | | | | | | | | | | | | | | | | | | 0 | | | | | | | | | | | | | | | | | | | | | | | 0.0 | | | | | | | | | | | | | | | | | |  | | | | | | | | | | | |
|  | | | | | | | | | | | | | | | | | | | | | | | | | | | | | | | |  | | | | | | | | | | | | | | | | | | | | | | | | | | | | | | | | | | |  | | | | | | | | | | | | | | | | | | | | | | | | | |  | | | | | | | | | | | | | | | | | | | |  | | | | | | | | | | | | | | | | | | | | | | |  | | | | | | | | | | | | | | | | | |  | | | | | | | | | | | |
| **Naps during the day** | | | | | | | | | | | | | | | | | | | | | | | | | | | | | | | | | | | Daily | | | | | | | | | | | | | | | | | | | | | | | | | | | | | | | | 108 | | | | | | | | | | | | | | | | | | | | | | | | | | 13.5 | | | | | | | | | | | | | | | | | | | | 29 | | | | | | | | | | | | | | | | | | | | | | | 14.7 | | | | | | | | | | | | | | | | | | 0.879 | | | | | | | | | | | |
|  | | | | | | | | | | | | | | | | | | | | | | | | | | | | | | | | | | | Frequently | | | | | | | | | | | | | | | | | | | | | | | | | | | | | | | | 35 | | | | | | | | | | | | | | | | | | | | | | | | | | 4.4 | | | | | | | | | | | | | | | | | | | | 7 | | | | | | | | | | | | | | | | | | | | | | | 3.5 | | | | | | | | | | | | | | | | | |  | | | | | | | | | | | |
|  | | | | | | | | | | | | | | | | | | | | | | | | | | | | | | | | | | | Sometimes | | | | | | | | | | | | | | | | | | | | | | | | | | | | | | | | 301 | | | | | | | | | | | | | | | | | | | | | | | | | | 37.6 | | | | | | | | | | | | | | | | | | | | 70 | | | | | | | | | | | | | | | | | | | | | | | 35.4 | | | | | | | | | | | | | | | | | |  | | | | | | | | | | | |
|  | | | | | | | | | | | | | | | | | | | | | | | | | | | | | | | | | | | Rarely | | | | | | | | | | | | | | | | | | | | | | | | | | | | | | | | 38 | | | | | | | | | | | | | | | | | | | | | | | | | | 4.7 | | | | | | | | | | | | | | | | | | | | 12 | | | | | | | | | | | | | | | | | | | | | | | 6.1 | | | | | | | | | | | | | | | | | |  | | | | | | | | | | | |
|  | | | | | | | | | | | | | | | | | | | | | | | | | | | | | | | | | | | Never | | | | | | | | | | | | | | | | | | | | | | | | | | | | | | | | 319 | | | | | | | | | | | | | | | | | | | | | | | | | | 39.8 | | | | | | | | | | | | | | | | | | | | 80 | | | | | | | | | | | | | | | | | | | | | | | 40.4 | | | | | | | | | | | | | | | | | |  | | | | | | | | | | | |
| If yes, naps under a tree | | | | | | | | | | | | | | | | | | | | | | | | | | | | | | | | | | | Daily | | | | | | | | | | | | | | | | | | | | | | | | | | | | | | | | 16 | | | | | | | | | | | | | | | | | | | | | | | | | | 3.3 | | | | | | | | | | | | | | | | | | | | 5 | | | | | | | | | | | | | | | | | | | | | | | 4.2 | | | | | | | | | | | | | | | | | | 0.722 | | | | | | | | | | | |
|  | | | | | | | | | | | | | | | | | | | | | | | | | | | | | | | | | | | Frequently | | | | | | | | | | | | | | | | | | | | | | | | | | | | | | | | 10 | | | | | | | | | | | | | | | | | | | | | | | | | | 2.1 | | | | | | | | | | | | | | | | | | | | 4 | | | | | | | | | | | | | | | | | | | | | | | 3.4 | | | | | | | | | | | | | | | | | |  | | | | | | | | | | | |
|  | | | | | | | | | | | | | | | | | | | | | | | | | | | | | | | | | | | Sometimes | | | | | | | | | | | | | | | | | | | | | | | | | | | | | | | | 113 | | | | | | | | | | | | | | | | | | | | | | | | | | 23.4 | | | | | | | | | | | | | | | | | | | | 26 | | | | | | | | | | | | | | | | | | | | | | | 22.0 | | | | | | | | | | | | | | | | | |  | | | | | | | | | | | |
|  | | | | | | | | | | | | | | | | | | | | | | | | | | | | | | | | | | | Rarely | | | | | | | | | | | | | | | | | | | | | | | | | | | | | | | | 8 | | | | | | | | | | | | | | | | | | | | | | | | | | 1.7 | | | | | | | | | | | | | | | | | | | | 3 | | | | | | | | | | | | | | | | | | | | | | | 2.5 | | | | | | | | | | | | | | | | | |  | | | | | | | | | | | |
|  | | | | | | | | | | | | | | | | | | | | | | | | | | | | | | | | | | | Never | | | | | | | | | | | | | | | | | | | | | | | | | | | | | | | | 335 | | | | | | | | | | | | | | | | | | | | | | | | | | 69.5 | | | | | | | | | | | | | | | | | | | | 80 | | | | | | | | | | | | | | | | | | | | | | | 67.8 | | | | | | | | | | | | | | | | | |  | | | | | | | | | | | |
| If yes, type | | | | | | | Neem (*Azadirachta indica*) | | | | | | | | | | | | | | | | | | | | | | | | | | | | | | | | | | | | | | | | | | | | | | | | | | | | | | | | | | | | 85 | | | | | | | | | | | | | | | | | | | | | | | | | | 57.8 | | | | | | | | | | | | | | | | | | | | 20 | | | | | | | | | | | | | | | | | | | | | | | 52.6 | | | | | | | | | | | | | | | | | | 0.565 | | | | | | | | | | | |
| of tree | | | | | | | Lalob/ higleeg (*Balanites aegyptica*) | | | | | | | | | | | | | | | | | | | | | | | | | | | | | | | | | | | | | | | | | | | | | | | | | | | | | | | | | | | | 54 | | | | | | | | | | | | | | | | | | | | | | | | | | 36.7 | | | | | | | | | | | | | | | | | | | | 14 | | | | | | | | | | | | | | | | | | | | | | | 36.8 | | | | | | | | | | | | | | | | | | 0.990 | | | | | | | | | | | |
| (n=185) | | | | | | | Sidr (*Ziziphus spina-christi*) | | | | | | | | | | | | | | | | | | | | | | | | | | | | | | | | | | | | | | | | | | | | | | | | | | | | | | | | | | | | 26 | | | | | | | | | | | | | | | | | | | | | | | | | | 17.7 | | | | | | | | | | | | | | | | | | | | 9 | | | | | | | | | | | | | | | | | | | | | | | 23.7 | | | | | | | | | | | | | | | | | | 0.400 | | | | | | | | | | | |
|  | | | | | | | Hashab (*Acacia senegal*) | | | | | | | | | | | | | | | | | | | | | | | | | | | | | | | | | | | | | | | | | | | | | | | | | | | | | | | | | | | | 11 | | | | | | | | | | | | | | | | | | | | | | | | | | 7.5 | | | | | | | | | | | | | | | | | | | | 3 | | | | | | | | | | | | | | | | | | | | | | | 7.9 | | | | | | | | | | | | | | | | | | 1.000 | | | | | | | | | | | |
|  | | | | | | | Sonot/Garat (*Acacia nilotica*) | | | | | | | | | | | | | | | | | | | | | | | | | | | | | | | | | | | | | | | | | | | | | | | | | | | | | | | | | | | | 8 | | | | | | | | | | | | | | | | | | | | | | | | | | 5.4 | | | | | | | | | | | | | | | | | | | | 5 | | | | | | | | | | | | | | | | | | | | | | | 13.2 | | | | | | | | | | | | | | | | | | 0.146 | | | | | | | | | | | |
|  | | | | | | | Taleh (*Acacia seyal*) | | | | | | | | | | | | | | | | | | | | | | | | | | | | | | | | | | | | | | | | | | | | | | | | | | | | | | | | | | | | 6 | | | | | | | | | | | | | | | | | | | | | | | | | | 4.1 | | | | | | | | | | | | | | | | | | | | 2 | | | | | | | | | | | | | | | | | | | | | | | 5.3 | | | | | | | | | | | | | | | | | | 0.669 | | | | | | | | | | | |
|  | | | | | | | Kiter (*Acacia Mellifera*) | | | | | | | | | | | | | | | | | | | | | | | | | | | | | | | | | | | | | | | | | | | | | | | | | | | | | | | | | | | | 3 | | | | | | | | | | | | | | | | | | | | | | | | | | 2.0 | | | | | | | | | | | | | | | | | | | | 1 | | | | | | | | | | | | | | | | | | | | | | | 2.6 | | | | | | | | | | | | | | | | | | 1.000 | | | | | | | | | | | |
|  | | | | | | | Other tree | | | | | | | | | | | | | | | | | | | | | | | | | | | | | | | | | | | | | | | | | | | | | | | | | | | | | | | | | | | | 11 | | | | | | | | | | | | | | | | | | | | | | | | | | 7.5 | | | | | | | | | | | | | | | | | | | | 3 | | | | | | | | | | | | | | | | | | | | | | | 7.9 | | | | | | | | | | | | | | | | | | 1.000 | | | | | | | | | | | |
| **Time when goes to sleep** | | | | | | | | | | | | | | | | | | | | | | | | | | | | | | | | | | | | | Before sunset | | | | | | | | | | | | | | | | | | | | | | | | | | | | | | 5 | | | | | | | | | | | | | | | | | | | | | | | | | | 0.6 | | | | | | | | | | | | | | | | | | | | 0 | | | | | | | | | | | | | | | | | | | | | | | 0.0 | | | | | | | | | | | | | | | | | | 0.338 | | | | | | | | | | | |
|  | | | | | | | | | | | | | | | | | | | | | | | | | | | | | | | | | | | | | At sunset | | | | | | | | | | | | | | | | | | | | | | | | | | | | | | 34 | | | | | | | | | | | | | | | | | | | | | | | | | | 4.2 | | | | | | | | | | | | | | | | | | | | 11 | | | | | | | | | | | | | | | | | | | | | | | 5.6 | | | | | | | | | | | | | | | | | |  | | | | | | | | | | | |
|  | | | | | | | | | | | | | | | | | | | | | | | | | | | | | | | | | | | | | After sunset | | | | | | | | | | | | | | | | | | | | | | | | | | | | | | 731 | | | | | | | | | | | | | | | | | | | | | | | | | | 91.3 | | | | | | | | | | | | | | | | | | | | 184 | | | | | | | | | | | | | | | | | | | | | | | 92.9 | | | | | | | | | | | | | | | | | |  | | | | | | | | | | | |
|  | | | | | | | | | | | | | | | | | | | | | | | | | | | | | | | | | | | | | Very variable | | | | | | | | | | | | | | | | | | | | | | | | | | | | | | 16 | | | | | | | | | | | | | | | | | | | | | | | | | | 2.0 | | | | | | | | | | | | | | | | | | | | 3 | | | | | | | | | | | | | | | | | | | | | | | 1.5 | | | | | | | | | | | | | | | | | |  | | | | | | | | | | | |
|  | | | | | | | | | | | | | | | | | | | | | | | | | | | | | | | | | | | | | Not relevant | | | | | | | | | | | | | | | | | | | | | | | | | | | | | | 14 | | | | | | | | | | | | | | | | | | | | | | | | | | 1.8 | | | | | | | | | | | | | | | | | | | | 0 | | | | | | | | | | | | | | | | | | | | | | | 0.0 | | | | | | | | | | | | | | | | | |  | | | | | | | | | | | |
|  | | | | | | | | | | | | | | | | | | | | | | | | | | | | | | | | | | | | | Don’t know | | | | | | | | | | | | | | | | | | | | | | | | | | | | | | 1 | | | | | | | | | | | | | | | | | | | | | | | | | | 0.1 | | | | | | | | | | | | | | | | | | | | 0 | | | | | | | | | | | | | | | | | | | | | | | 0.0 | | | | | | | | | | | | | | | | | |  | | | | | | | | | | | |
| **Bedtime hour** | | | | | | | | | | | | | | | | | | | | | | | | | | | | | | | | | | | | | | | | | | | | | | 17 to 18h | | | | | | | | | | | | | | | | | | | | | 29 | | | | | | | | | | | | | | | | | | | | | | | | | | 3.7 | | | | | | | | | | | | | | | | | | | | 9 | | | | | | | | | | | | | | | | | | | | | | | 4.6 | | | | | | | | | | | | | | | | | | 0.003 | | | | | | | | | | | |
|  | | | | | | | | | | | | | | | | | | | | | | | | | | | | | | | | | | | | | | | | | | | | | | 19 to 20h | | | | | | | | | | | | | | | | | | | | | 308 | | | | | | | | | | | | | | | | | | | | | | | | | | 39.3 | | | | | | | | | | | | | | | | | | | | 103 | | | | | | | | | | | | | | | | | | | | | | | 52.5 | | | | | | | | | | | | | | | | | |  | | | | | | | | | | | |
|  | | | | | | | | | | | | | | | | | | | | | | | | | | | | | | | | | | | | | | | | | | | | | | 21 to 22h | | | | | | | | | | | | | | | | | | | | | 381 | | | | | | | | | | | | | | | | | | | | | | | | | | 48.7 | | | | | | | | | | | | | | | | | | | | 77 | | | | | | | | | | | | | | | | | | | | | | | 39.3 | | | | | | | | | | | | | | | | | |  | | | | | | | | | | | |
|  | | | | | | | | | | | | | | | | | | | | | | | | | | | | | | | | | | | | | | | | | | | | | | 23h to 2h | | | | | | | | | | | | | | | | | | | | | 65 | | | | | | | | | | | | | | | | | | | | | | | | | | 8.3 | | | | | | | | | | | | | | | | | | | | 7 | | | | | | | | | | | | | | | | | | | | | | | 3.6 | | | | | | | | | | | | | | | | | |  | | | | | | | | | | | |
| **Goes out before sunrise** | | | | | | | | | | | | | | | | | | | | | | | | | | | | | | | | | | | | | | | | | | | | | | Daily | | | | | | | | | | | | | | | | | | | | | 102 | | | | | | | | | | | | | | | | | | | | | | | | | | 13.3 | | | | | | | | | | | | | | | | | | | | 30 | | | | | | | | | | | | | | | | | | | | | | | 16.1 | | | | | | | | | | | | | | | | | | 0.667 | | | | | | | | | | | |
| (age ≥1 and sleeping inside) | | | | | | | | | | | | | | | | | | | | | | | | | | | | | | | | | | | | | | | | | | | | | | Frequently | | | | | | | | | | | | | | | | | | | | | 25 | | | | | | | | | | | | | | | | | | | | | | | | | | 3.3 | | | | | | | | | | | | | | | | | | | | 6 | | | | | | | | | | | | | | | | | | | | | | | 3.1 | | | | | | | | | | | | | | | | | |  | | | | | | | | | | | |
|  | | | | | | | | | | | | | | | | | | | | | | | | | | | | | | | | | | | | | | | | | | | | | | Sometimes | | | | | | | | | | | | | | | | | | | | | 281 | | | | | | | | | | | | | | | | | | | | | | | | | | 36.7 | | | | | | | | | | | | | | | | | | | | 73 | | | | | | | | | | | | | | | | | | | | | | | 37.8 | | | | | | | | | | | | | | | | | |  | | | | | | | | | | | |
|  | | | | | | | | | | | | | | | | | | | | | | | | | | | | | | | | | | | | | | | | | | | | | | Rarely | | | | | | | | | | | | | | | | | | | | | 19 | | | | | | | | | | | | | | | | | | | | | | | | | | 2.5 | | | | | | | | | | | | | | | | | | | | 2 | | | | | | | | | | | | | | | | | | | | | | | 1.0 | | | | | | | | | | | | | | | | | |  | | | | | | | | | | | |
|  | | | | | | | | | | | | | | | | | | | | | | | | | | | | | | | | | | | | | | | | | | | | | | Never | | | | | | | | | | | | | | | | | | | | | 339 | | | | | | | | | | | | | | | | | | | | | | | | | | 44.3 | | | | | | | | | | | | | | | | | | | | 80 | | | | | | | | | | | | | | | | | | | | | | | 42.0 | | | | | | | | | | | | | | | | | |  | | | | | | | | | | | |
| **Place of stay between sunset and sleep**  (50 going to bed before or at sunset excluded) | | | | | | | | | | | | | | | | | | | | | | | | | | | | | | | | | | | | | | | | | | | | | | | | | | | | | | | | | | | | | | | | | | |  | | | | | | | | | | | | | | | | | | | | | | | | | |  | | | | | | | | | | | | | | | | | | | |  | | | | | | | | | | | | | | | | | | | | | | |  | | | | | | | | | | | | | | | | | | 0.037 | | | | | | | | | | | |
|  | | | | | | | | | Indoor only | | | | | | | | | | | | | | | | | | | | | | | | | | | | | | | | | | | | | | | | | | | | | | | | | | | | | | | | | | 213 | | | | | | | | | | | | | | | | | | | | | | | | | | 27.9 | | | | | | | | | | | | | | | | | | | | 44 | | | | | | | | | | | | | | | | | | | | | | | 23.5 | | | | | | | | | | | | | | | | | |  | | | | | | | | | | | |
|  | | | | | | | | | Outdoor | | | | | | | | | | | | | | | | | | | | | | | | | | | | | | | | | | | | | | | | | | | | | | | | | | | | | | | | | | 314 | | | | | | | | | | | | | | | | | | | | | | | | | | 41.2 | | | | | | | | | | | | | | | | | | | | 67 | | | | | | | | | | | | | | | | | | | | | | | 35.8 | | | | | | | | | | | | | | | | | |  | | | | | | | | | | | |
|  | | | | | | | | | Both indoor and outdoor | | | | | | | | | | | | | | | | | | | | | | | | | | | | | | | | | | | | | | | | | | | | | | | | | | | | | | | | | | 235 | | | | | | | | | | | | | | | | | | | | | | | | | | 30.8 | | | | | | | | | | | | | | | | | | | | 76 | | | | | | | | | | | | | | | | | | | | | | | 40.6 | | | | | | | | | | | | | | | | | |  | | | | | | | | | | | |
| **Main outdoor activities after sunset** (1 missing) | | | | | | | | | | | | | | | | | | | | | | | | | | | | | | | | | | | | | | | | | | | | | | | | | | | | | | | | | | | | | | | | | | |  | | | | | | | | | | | | | | | | | | | | | | | | | |  | | | | | | | | | | | | | | | | | | | |  | | | | | | | | | | | | | | | | | | | | | | |  | | | | | | | | | | | | | | | | | | <0.001 | | | | | | | | | | | |
|  | | | | | | | | | Stay indoor only | | | | | | | | | | | | | | | | | | | | | | | | | | | | | | | | | | | | | | | | | | | | | | | | | | | | | | | | | | 243 | | | | | | | | | | | | | | | | | | | | | | | | | | 30.4 | | | | | | | | | | | | | | | | | | | | 53 | | | | | | | | | | | | | | | | | | | | | | | 26.8 | | | | | | | | | | | | | | | | | |  | | | | | | | | | | | |
|  | | | | | | | | | Playing | | | | | | | | | | | | | | | | | | | | | | | | | | | | | | | | | | | | | | | | | | | | | | | | | | | | | | | | | | 154 | | | | | | | | | | | | | | | | | | | | | | | | | | 19.3 | | | | | | | | | | | | | | | | | | | | 65 | | | | | | | | | | | | | | | | | | | | | | | 32.8 | | | | | | | | | | | | | | | | | |  | | | | | | | | | | | |
|  | | | | | | | | | TV / radio | | | | | | | | | | | | | | | | | | | | | | | | | | | | | | | | | | | | | | | | | | | | | | | | | | | | | | | | | | 52 | | | | | | | | | | | | | | | | | | | | | | | | | | 6.5 | | | | | | | | | | | | | | | | | | | | 27 | | | | | | | | | | | | | | | | | | | | | | | 13.6 | | | | | | | | | | | | | | | | | |  | | | | | | | | | | | |
|  | | | | | | | | | Discussing-relaxing | | | | | | | | | | | | | | | | | | | | | | | | | | | | | | | | | | | | | | | | | | | | | | | | | | | | | | | | | | 265 | | | | | | | | | | | | | | | | | | | | | | | | | | 33.1 | | | | | | | | | | | | | | | | | | | | 47 | | | | | | | | | | | | | | | | | | | | | | | 23.7 | | | | | | | | | | | | | | | | | |  | | | | | | | | | | | |
|  | | | | | | | | | Cooking/house activities | | | | | | | | | | | | | | | | | | | | | | | | | | | | | | | | | | | | | | | | | | | | | | | | | | | | | | | | | | 47 | | | | | | | | | | | | | | | | | | | | | | | | | | 5.9 | | | | | | | | | | | | | | | | | | | | 4 | | | | | | | | | | | | | | | | | | | | | | | 2.0 | | | | | | | | | | | | | | | | | |  | | | | | | | | | | | |
|  | | | | | | | | | Selling at the market | | | | | | | | | | | | | | | | | | | | | | | | | | | | | | | | | | | | | | | | | | | | | | | | | | | | | | | | | | 11 | | | | | | | | | | | | | | | | | | | | | | | | | | 1.4 | | | | | | | | | | | | | | | | | | | | 1 | | | | | | | | | | | | | | | | | | | | | | | 0.5 | | | | | | | | | | | | | | | | | |  | | | | | | | | | | | |
|  | | | | | | | | | Farming / herding animals | | | | | | | | | | | | | | | | | | | | | | | | | | | | | | | | | | | | | | | | | | | | | | | | | | | | | | | | | | 6 | | | | | | | | | | | | | | | | | | | | | | | | | | 0.8 | | | | | | | | | | | | | | | | | | | | 0 | | | | | | | | | | | | | | | | | | | | | | | 0.0 | | | | | | | | | | | | | | | | | |  | | | | | | | | | | | |
|  | | | | | | | | | Reading/lessons | | | | | | | | | | | | | | | | | | | | | | | | | | | | | | | | | | | | | | | | | | | | | | | | | | | | | | | | | | 22 | | | | | | | | | | | | | | | | | | | | | | | | | | 2.8 | | | | | | | | | | | | | | | | | | | | 1 | | | | | | | | | | | | | | | | | | | | | | | 0.5 | | | | | | | | | | | | | | | | | |  | | | | | | | | | | | |
| **Location outside** (among participants reporting outdoor after sunset) | | | | | | | | | | | | | | | | | | | | | | | | | | | | | | | | | | | | | | | | | | | | | | | | | | | | | | | | | | | | | | | | | | | | | | | | | | | | | | | | | | | | | | | | | | | | | | | | | | | | | | | | | | | | | | | | | | | | | | | | | | | | | | | | | | | | | | | | | | | | | | | | | | | | | | | | | | 0.289 | | | | | | | | | | | |
|  | | | | | House yard | | | | | | | | | | | | | | | | | | | | | | | | | | | | | | | | | | | | | | | | | | | | | | | | | | | | | | | | | | | | | | 430 | | | | | | | | | | | | | | | | | | | | | | | | | | 78.3 | | | | | | | | | | | | | | | | | | | | 113 | | | | | | | | | | | | | | | | | | | | | | | 79.0 | | | | | | | | | | | | | | | | | |  | | | | | | | | | | | |
|  | | | | | Market | | | | | | | | | | | | | | | | | | | | | | | | | | | | | | | | | | | | | | | | | | | | | | | | | | | | | | | | | | | | | | 35 | | | | | | | | | | | | | | | | | | | | | | | | | | 6.4 | | | | | | | | | | | | | | | | | | | | 5 | | | | | | | | | | | | | | | | | | | | | | | 3.5 | | | | | | | | | | | | | | | | | |  | | | | | | | | | | | |
|  | | | | | Fields | | | | | | | | | | | | | | | | | | | | | | | | | | | | | | | | | | | | | | | | | | | | | | | | | | | | | | | | | | | | | | 5 | | | | | | | | | | | | | | | | | | | | | | | | | | 0.9 | | | | | | | | | | | | | | | | | | | | 0 | | | | | | | | | | | | | | | | | | | | | | | 0.0 | | | | | | | | | | | | | | | | | |  | | | | | | | | | | | |
|  | | | | | Next to house yard | | | | | | | | | | | | | | | | | | | | | | | | | | | | | | | | | | | | | | | | | | | | | | | | | | | | | | | | | | | | | | 57 | | | | | | | | | | | | | | | | | | | | | | | | | | 10.4 | | | | | | | | | | | | | | | | | | | | 18 | | | | | | | | | | | | | | | | | | | | | | | 12.6 | | | | | | | | | | | | | | | | | |  | | | | | | | | | | | |
|  | | | | | In the village, other | | | | | | | | | | | | | | | | | | | | | | | | | | | | | | | | | | | | | | | | | | | | | | | | | | | | | | | | | | | | | | 22 | | | | | | | | | | | | | | | | | | | | | | | | | | 4.0 | | | | | | | | | | | | | | | | | | | | 6 | | | | | | | | | | | | | | | | | | | | | | | 4.2 | | | | | | | | | | | | | | | | | |  | | | | | | | | | | | |
|  | | | | | In a neighbouring village | | | | | | | | | | | | | | | | | | | | | | | | | | | | | | | | | | | | | | | | | | | | | | | | | | | | | | | | | | | | | | 0 | | | | | | | | | | | | | | | | | | | | | | | | | | 0.0 | | | | | | | | | | | | | | | | | | | | 1 | | | | | | | | | | | | | | | | | | | | | | | 0.7 | | | | | | | | | | | | | | | | | |  | | | | | | | | | | | |
|  | | | | | | | | | | | | | | | | | | | | | | | | | | | | | | | | | | | | | | | | | | | | | |  | | | | | | | | | | | | | | | | | | | | |  | | | | | | | | | | | | | | | | | | | | | | | | | |  | | | | | | | | | | | | | | | | | | | |  | | | | | | | | | | | | | | | | | | | | | | |  | | | | | | | | | | | | | | | | | |  | | | | | | | | | | | |
| **Goes out early morning (sunrise)** | | | | | | | | | | | | | | | | | | | | | | | | | | | | | | | | | | | | | | | | | | | | | | Daily | | | | | | | | | | | | | | | | | | | | | 258 | | | | | | | | | | | | | | | | | | | | | | | | | | 33.7 | | | | | | | | | | | | | | | | | | | | 57 | | | | | | | | | | | | | | | | | | | | | | | 29.5 | | | | | | | | | | | | | | | | | | 0.420 | | | | | | | | | | | |
| (age ≥1 and sleeping inside) | | | | | | | | | | | | | | | | | | | | | | | | | | | | | | | | | | | | | | | | | | | | | | Frequently | | | | | | | | | | | | | | | | | | | | | 25 | | | | | | | | | | | | | | | | | | | | | | | | | | 3.3 | | | | | | | | | | | | | | | | | | | | 3 | | | | | | | | | | | | | | | | | | | | | | | 1.6 | | | | | | | | | | | | | | | | | |  | | | | | | | | | | | |
|  | | | | | | | | | | | | | | | | | | | | | | | | | | | | | | | | | | | | | | | | | | | | | | Sometimes | | | | | | | | | | | | | | | | | | | | | 148 | | | | | | | | | | | | | | | | | | | | | | | | | | 19.3 | | | | | | | | | | | | | | | | | | | | 36 | | | | | | | | | | | | | | | | | | | | | | | 18.7 | | | | | | | | | | | | | | | | | |  | | | | | | | | | | | |
|  | | | | | | | | | | | | | | | | | | | | | | | | | | | | | | | | | | | | | | | | | | | | | | Rarely | | | | | | | | | | | | | | | | | | | | | 12 | | | | | | | | | | | | | | | | | | | | | | | | | | 1.6 | | | | | | | | | | | | | | | | | | | | 4 | | | | | | | | | | | | | | | | | | | | | | | 2.1 | | | | | | | | | | | | | | | | | |  | | | | | | | | | | | |
|  | | | | | | | | | | | | | | | | | | | | | | | | | | | | | | | | | | | | | | | | | | | | | | Never | | | | | | | | | | | | | | | | | | | | | 323 | | | | | | | | | | | | | | | | | | | | | | | | | | 42.2 | | | | | | | | | | | | | | | | | | | | 93 | | | | | | | | | | | | | | | | | | | | | | | 48.2 | | | | | | | | | | | | | | | | | |  | | | | | | | | | | | |
| **Sleep location** | | | | | | | | | | | | | | | | | In the house yard | | | | | | | | | | | | | | | | | | | | | | | | | | | | | | | | | | | | | | | | | | | | | | | | | | 778 | | | | | | | | | | | | | | | | | | | | | | | | | | 97.1 | | | | | | | | | | | | | | | | | | | | 183 | | | | | | | | | | | | | | | | | | | | | | | 92.4 | | | | | | | | | | | | | | | | | | 0.004 | | | | | | | | | | | |
|  | | | | | | | | | | | | | | | | | In the village but not in the yard | | | | | | | | | | | | | | | | | | | | | | | | | | | | | | | | | | | | | | | | | | | | | | | | | | 19 | | | | | | | | | | | | | | | | | | | | | | | | | | 2.4 | | | | | | | | | | | | | | | | | | | | 15 | | | | | | | | | | | | | | | | | | | | | | | 7.6 | | | | | | | | | | | | | | | | | |  | | | | | | | | | | | |
|  | | | | | | | | | | | | | | | | | In a neighbouring village | | | | | | | | | | | | | | | | | | | | | | | | | | | | | | | | | | | | | | | | | | | | | | | | | | 1 | | | | | | | | | | | | | | | | | | | | | | | | | | 0.1 | | | | | | | | | | | | | | | | | | | | 0 | | | | | | | | | | | | | | | | | | | | | | | 0.0 | | | | | | | | | | | | | | | | | |  | | | | | | | | | | | |
|  | | | | | | | | | | | | | | | | | Farm/field | | | | | | | | | | | | | | | | | | | | | | | | | | | | | | | | | | | | | | | | | | | | | | | | | | 3 | | | | | | | | | | | | | | | | | | | | | | | | | | 0.4 | | | | | | | | | | | | | | | | | | | | 0 | | | | | | | | | | | | | | | | | | | | | | | 0.0 | | | | | | | | | | | | | | | | | |  | | | | | | | | | | | |
| **Usually sleeping on** | | | | | | | | | | | | | | | | | | | | | | | | | | | | | | | | | a bed | | | | | | | | | | | | | | | | | | | | | | | | | | | | | | | | | | 793 | | | | | | | | | | | | | | | | | | | | | | | | | | 99.1 | | | | | | | | | | | | | | | | | | | | 195 | | | | | | | | | | | | | | | | | | | | | | | 98.4 | | | | | | | | | | | | | | | | | | 0.378 | | | | | | | | | | | |
| (1 missing) | | | | | | | | | | | | | | | | | | | | | | | | | | | | | | | | | the floor | | | | | | | | | | | | | | | | | | | | | | | | | | | | | | | | | | 6 | | | | | | | | | | | | | | | | | | | | | | | | | | 0.7 | | | | | | | | | | | | | | | | | | | | 2 | | | | | | | | | | | | | | | | | | | | | | | 1.0 | | | | | | | | | | | | | | | | | |  | | | | | | | | | | | |
|  | | | | | | | | | | | | | | | | | | | | | | | | | | | | | | | | | a mat or mattress | | | | | | | | | | | | | | | | | | | | | | | | | | | | | | | | | | 1 | | | | | | | | | | | | | | | | | | | | | | | | | | 0.1 | | | | | | | | | | | | | | | | | | | | 1 | | | | | | | | | | | | | | | | | | | | | | | 0.5 | | | | | | | | | | | | | | | | | |  | | | | | | | | | | | |
| **Place of sleep** | | | | | | | | | | | | | | | | | | | | | | | | | | | | | | In a brick/cement room | | | | | | | | | | | | | | | | | | | | | | | | | | | | | | | | | | | | | 10 | | | | | | | | | | | | | | | | | | | | | | | | | | 1.3 | | | | | | | | | | | | | | | | | | | | 5 | | | | | | | | | | | | | | | | | | | | | | | 2.5 | | | | | | | | | | | | | | | | | | 0.020 | | | | | | | | | | | |
|  | | | | | | | | | | | | | | | | | | | | | | | | | | | | | | In a tukul | | | | | | | | | | | | | | | | | | | | | | | | | | | | | | | | | | | | | 736 | | | | | | | | | | | | | | | | | | | | | | | | | | 91.9 | | | | | | | | | | | | | | | | | | | | 188 | | | | | | | | | | | | | | | | | | | | | | | 95.0 | | | | | | | | | | | | | | | | | |  | | | | | | | | | | | |
|  | | | | | | | | | | | | | | | | | | | | | | | | | | | | | | In a “local” room | | | | | | | | | | | | | | | | | | | | | | | | | | | | | | | | | | | | | 38 | | | | | | | | | | | | | | | | | | | | | | | | | | 4.7 | | | | | | | | | | | | | | | | | | | | 1 | | | | | | | | | | | | | | | | | | | | | | | 0.5 | | | | | | | | | | | | | | | | | |  | | | | | | | | | | | |
|  | | | | | | | | | | | | | | | | | | | | | | | | | | | | | | Under a shelter | | | | | | | | | | | | | | | | | | | | | | | | | | | | | | | | | | | | | 4 | | | | | | | | | | | | | | | | | | | | | | | | | | 0.5 | | | | | | | | | | | | | | | | | | | | 0 | | | | | | | | | | | | | | | | | | | | | | | 0.0 | | | | | | | | | | | | | | | | | |  | | | | | | | | | | | |
|  | | | | | | | | | | | | | | | | | | | | | | | | | | | | | | In an open space | | | | | | | | | | | | | | | | | | | | | | | | | | | | | | | | | | | | | 5 | | | | | | | | | | | | | | | | | | | | | | | | | | 0.6 | | | | | | | | | | | | | | | | | | | | 1 | | | | | | | | | | | | | | | | | | | | | | | 0.5 | | | | | | | | | | | | | | | | | |  | | | | | | | | | | | |
|  | | | | | | | | | | | | | | | | | | | | | | | | | | | | | | Variable | | | | | | | | | | | | | | | | | | | | | | | | | | | | | | | | | | | | | 8 | | | | | | | | | | | | | | | | | | | | | | | | | | 1.0 | | | | | | | | | | | | | | | | | | | | 3 | | | | | | | | | | | | | | | | | | | | | | | 1.5 | | | | | | | | | | | | | | | | | |  | | | | | | | | | | | |
| **Sleeping covered** | | | | | | | | | | | | | | | | | | | | | | | | | | | | | | Covered | | | | | | | | | | | | | | | | | | | | | | | | | | | | | | | | | | | | | 580 | | | | | | | | | | | | | | | | | | | | | | | | | | 72.4 | | | | | | | | | | | | | | | | | | | | 126 | | | | | | | | | | | | | | | | | | | | | | | 63.6 | | | | | | | | | | | | | | | | | | 0.050 | | | | | | | | | | | |
|  | | | | | | | | | | | | | | | | | | | | | | | | | | | | | | Not covered | | | | | | | | | | | | | | | | | | | | | | | | | | | | | | | | | | | | | 193 | | | | | | | | | | | | | | | | | | | | | | | | | | 24.1 | | | | | | | | | | | | | | | | | | | | 62 | | | | | | | | | | | | | | | | | | | | | | | 31.3 | | | | | | | | | | | | | | | | | |  | | | | | | | | | | | |
|  | | | | | | | | | | | | | | | | | | | | | | | | | | | | | | Variable | | | | | | | | | | | | | | | | | | | | | | | | | | | | | | | | | | | | | 28 | | | | | | | | | | | | | | | | | | | | | | | | | | 3.5 | | | | | | | | | | | | | | | | | | | | 10 | | | | | | | | | | | | | | | | | | | | | | | 5.1 | | | | | | | | | | | | | | | | | |  | | | | | | | | | | | |
| ***If covered, with*** | | | | | | | | | | | | | | | | | | | | | | | | | | | | | | | | | | | | | | | *Sheet* | | | | | | | | | | | | | | | | | | | | | | | | | | | | *528* | | | | | | | | | | | | | | | | | | | | | | | | | | *65.9* | | | | | | | | | | | | | | | | | | | | *120* | | | | | | | | | | | | | | | | | | | | | | | *60.6* | | | | | | | | | | | | | | | | | |  | | | | | | | | | | | |
|  | | | | | | | | | | | | | | | | | | | | | | | | | | | | | | | | | | | | | | | *Blanket* | | | | | | | | | | | | | | | | | | | | | | | | | | | | *79* | | | | | | | | | | | | | | | | | | | | | | | | | | *9.9* | | | | | | | | | | | | | | | | | | | | *15* | | | | | | | | | | | | | | | | | | | | | | | *7.6* | | | | | | | | | | | | | | | | | |  | | | | | | | | | | | |
|  | | | | | | | | | | | | | | | | | | | | | | | | | | | | | | | | | | | | | | | *Sheet or blanket* | | | | | | | | | | | | | | | | | | | | | | | | | | | | *1* | | | | | | | | | | | | | | | | | | | | | | | | | | *0.1* | | | | | | | | | | | | | | | | | | | | *1* | | | | | | | | | | | | | | | | | | | | | | | *0.5* | | | | | | | | | | | | | | | | | |  | | | | | | | | | | | |
| **Sleep under a mosquito-net** | | | | | | | | | | | | | | | | | | | | | | | | | | | | | | | | | | | | | | | | | | | | | | Never | | | | | | | | | | | | | | | | | | | | | 293 | | | | | | | | | | | | | | | | | | | | | | | | | | 36.6 | | | | | | | | | | | | | | | | | | | | 65 | | | | | | | | | | | | | | | | | | | | | | | 32.8 | | | | | | | | | | | | | | | | | | 0.324 | | | | | | | | | | | |
| **Frequency of mosquito-net use** | | | | | | | | | | | | | | | | | | | | | | | | | | | | | | | | | | | | | | | | | | | | | | Daily | | | | | | | | | | | | | | | | | | | | | 468 | | | | | | | | | | | | | | | | | | | | | | | | | | 58.4 | | | | | | | | | | | | | | | | | | | | 117 | | | | | | | | | | | | | | | | | | | | | | | 59.1 | | | | | | | | | | | | | | | | | | 0.218 | | | | | | | | | | | |
|  | | | | | | | | | | | | | | | | | | | | | | | | | | | | | | | | | | | | | | | | | | | | | | Frequently | | | | | | | | | | | | | | | | | | | | | 3 | | | | | | | | | | | | | | | | | | | | | | | | | | 0.4 | | | | | | | | | | | | | | | | | | | | 2 | | | | | | | | | | | | | | | | | | | | | | | 1.0 | | | | | | | | | | | | | | | | | |  | | | | | | | | | | | |
|  | | | | | | | | | | | | | | | | | | | | | | | | | | | | | | | | | | | | | | | | | | | | | | Sometimes | | | | | | | | | | | | | | | | | | | | | 36 | | | | | | | | | | | | | | | | | | | | | | | | | | 4.5 | | | | | | | | | | | | | | | | | | | | 13 | | | | | | | | | | | | | | | | | | | | | | | 6.6 | | | | | | | | | | | | | | | | | |  | | | | | | | | | | | |
|  | | | | | | | | | | | | | | | | | | | | | | | | | | | | | | | | | | | | | | | | | | | | | | Rarely | | | | | | | | | | | | | | | | | | | | | 1 | | | | | | | | | | | | | | | | | | | | | | | | | | 0.1 | | | | | | | | | | | | | | | | | | | | 1 | | | | | | | | | | | | | | | | | | | | | | | 0.5 | | | | | | | | | | | | | | | | | |  | | | | | | | | | | | |
|  | | | | | | | | | | | | | | | | | | | | | | | | | | | | | | | | | | | | | | | | | | | | | | Never | | | | | | | | | | | | | | | | | | | | | 293 | | | | | | | | | | | | | | | | | | | | | | | | | | 36.6 | | | | | | | | | | | | | | | | | | | | 65 | | | | | | | | | | | | | | | | | | | | | | | 32.8 | | | | | | | | | | | | | | | | | |  | | | | | | | | | | | |
| **Sleeping outside the village due to farming and herding activities – in the rainy season** | | | | | | | | | | | | | | | | | | | | | | | | | | | | | | | | | | | | | | | | | | | | | | | | | | | | | | | | | | | | | | | | | | | | | | | | | | | | | | | | | | | | | | | | | | | | | | | | | | | | | | | | | | | | | | | | | | | | | | | | | | | | | | | | | | | | | | | | | | | | | | | | | | | | | | | | | | | | | | | | | | | | | |
| **Sleeping in the field/farm** | | | | | | | | | | | | | | | | | | | | | | | | | | | | | | | | | | | | | | | | | | | | | | Yes | | | | | | | | | | | | | | | | | | | | | 124 | | | | | | | | | | | | | | | | | | | | | | | | | | 15.5 | | | | | | | | | | | | | | | | | | | | 17 | | | | | | | | | | | | | | | | | | | | | | | 8.6 | | | | | | | | | | | | | | | | | | 0.013 | | | | | | | | | | | |
|  | | | | | | | | | | | | | | | | | | | | | | | | | | | | | | | | | | | | | | | | | | | | | | Never | | | | | | | | | | | | | | | | | | | | | 676 | | | | | | | | | | | | | | | | | | | | | | | | | | 84.5 | | | | | | | | | | | | | | | | | | | | 181 | | | | | | | | | | | | | | | | | | | | | | | 91.4 | | | | | | | | | | | | | | | | | |  | | | | | | | | | | | |
| If yes, sleep location | | | | | | | | | | | | | | | | | | | | | | | | | | | | | | | | | | | | | | | Inside a tukul | | | | | | | | | | | | | | | | | | | | | | | | | | | | 38 | | | | | | | | | | | | | | | | | | | | | | | | | | 31.4 | | | | | | | | | | | | | | | | | | | | 5 | | | | | | | | | | | | | | | | | | | | | | | 29.4 | | | | | | | | | | | | | | | | | | 1.000 | | | | | | | | | | | |
| (1 don't know, 2 “variable”) | | | | | | | | | | | | | | | | | | | | | | | | | | | | | | | | | | | | | | | Under a shelter | | | | | | | | | | | | | | | | | | | | | | | | | | | | 1 | | | | | | | | | | | | | | | | | | | | | | | | | | 0.8 | | | | | | | | | | | | | | | | | | | | 0 | | | | | | | | | | | | | | | | | | | | | | | 0.0 | | | | | | | | | | | | | | | | | |  | | | | | | | | | | | |
|  | | | | | | | | | | | | | | | | | | | | | | | | | | | | | | | | | | | | | | | In an open space | | | | | | | | | | | | | | | | | | | | | | | | | | | | 79 | | | | | | | | | | | | | | | | | | | | | | | | | | 65.3 | | | | | | | | | | | | | | | | | | | | 12 | | | | | | | | | | | | | | | | | | | | | | | 70.6 | | | | | | | | | | | | | | | | | |  | | | | | | | | | | | |
|  | | | | | | | | | | | | | | | | | | | | | | | | | | | | | | | | | | | | | | | Under a tree | | | | | | | | | | | | | | | | | | | | | | | | | | | | 3 | | | | | | | | | | | | | | | | | | | | | | | | | | 2.5 | | | | | | | | | | | | | | | | | | | | 0 | | | | | | | | | | | | | | | | | | | | | | | 0.0 | | | | | | | | | | | | | | | | | |  | | | | | | | | | | | |
| When sleeping in the field/farm, bed-net use | | | | | | | | | | | | | | | | | | | | | | | | | | | | | | | | | | | | | | | | | | | | | | | | | | | | | | | | | | |  | | | | | | | |  | | | | | | | | | | | | | | | | | | | | | | | | | |  | | | | | | | | | | | | | | | | | | | |  | | | | | | | | | | | | | | | | | | | | | | |  | | | | | | | | | | | | | | | | | | 0.029 | | | | | | | | | | | |
| (1 don't know) | | | | | | | | | | | | | | | | | | | | | Daily/sometimes/frequently | | | | | | | | | | | | | | | | | | | | | | | | | | | | | | | | | | | | | | | | | | | | | | 67 | | | | | | | | | | | | | | | | | | | | | | | | | | 54.5 | | | | | | | | | | | | | | | | | | | | 14 | | | | | | | | | | | | | | | | | | | | | | | 82.3 | | | | | | | | | | | | | | | | | |  | | | | | | | | | | | |
|  | | | | | | | | | | | | | | | | | | | | | Rarely/never | | | | | | | | | | | | | | | | | | | | | | | | | | | | | | | | | | | | | | | | | | | | | | 56 | | | | | | | | | | | | | | | | | | | | | | | | | | 45.5 | | | | | | | | | | | | | | | | | | | | 3 | | | | | | | | | | | | | | | | | | | | | | | 17.6 | | | | | | | | | | | | | | | | | |  | | | | | | | | | | | |
| Nb of nights in the farm over the season | | | | | | | | | | | | | | | | | | | | | | | | | | | | | | | | | | | | | | | | | | | | | | | | | | | | | | | Median (IQR) | | | | | | | | | | | | | | | | | | | | | | | | | | | 7 | | | | | | | | | | | | | | | | | | | | | | (5,14) | | | | | | | | | | | | | | | | | | | | | 7 | | | | | | | | | | | (5,15) | | | | | | | | | | | | | | | | | | 0.758 | | | | | | | | | | | |
| **Herding animals** | | | | | | | | | | | | | | | | | | | | | | | | | | | | | | | | | | | | | | | | | | | | | | Yes | | | | | | | | | | | | | | | | | | | | | 254 | | | | | | | | | | | | | | | | | | | | | | | | | | 31.7 | | | | | | | | | | | | | | | | | | | | 59 | | | | | | | | | | | | | | | | | | | | | | | 29.8 | | | | | | | | | | | | | | | | | | 0.603 | | | | | | | | | | | |
|  | | | | | | | | | | | | | | | | | | | | | | | | | | | | | | | | | | | | | | | | | | | | | | Never | | | | | | | | | | | | | | | | | | | | | 547 | | | | | | | | | | | | | | | | | | | | | | | | | | 68.3 | | | | | | | | | | | | | | | | | | | | 139 | | | | | | | | | | | | | | | | | | | | | | | 70.2 | | | | | | | | | | | | | | | | | |  | | | | | | | | | | | |
| If yes, sleep outside village for herding | | | | | | | | | | | | | | | | | | | | | | | | | | | | | | | | | | | | | | | | | | | | | | | | | | | | Yes | | | | | | | | | | | | | | | 12 | | | | | | | | | | | | | | | | | | | | | | | | | | 4.7 | | | | | | | | | | | | | | | | | | | | 1 | | | | | | | | | | | | | | | | | | | | | | | 1.7 | | | | | | | | | | | | | | | | | | 0.475 | | | | | | | | | | | |
|  | | | | | | | | | | | | | | | | | | | | | | | | | | | | | | | | | | | | | | | | | | | | | | | | | | | | Never | | | | | | | | | | | | | | | 242 | | | | | | | | | | | | | | | | | | | | | | | | | | 95.3 | | | | | | | | | | | | | | | | | | | | 58 | | | | | | | | | | | | | | | | | | | | | | | 98.3 | | | | | | | | | | | | | | | | | |  | | | | | | | | | | | |
| If yes, location | | | | | | | | | | | | | | | | | | | | | | | | | | | | | | | | | | | In a tukul | | | | | | | | | | | | | | | | | | | | | | | | | | | | | | | | 0 | | | | | | | | | | | | | | | | | | | | | | | | | | 0.0 | | | | | | | | | | | | | | | | | | | | 0 | | | | | | | | | | | | | | | | | | | | | | | 0.0 | | | | | | | | | | | | | | | | | | NA | | | | | | | | | | | |
|  | | | | | | | | | | | | | | | | | | | | | | | | | | | | | | | | | | | In an open space | | | | | | | | | | | | | | | | | | | | | | | | | | | | | | | | 12 | | | | | | | | | | | | | | | | | | | | | | | | | | 100.0 | | | | | | | | | | | | | | | | | | | | 1 | | | | | | | | | | | | | | | | | | | | | | | 100.0 | | | | | | | | | | | | | | | | | |  | | | | | | | | | | | |
| If yes, bed-net use | | | | | | | | | | | | | | | | | | | | | | | | | | | | | | | | | | | | | | | | | | | | | Daily | | | | | | | | | | | | | | | | | | | | | | 1 | | | | | | | | | | | | | | | | | | | | | | | | | | 8.3 | | | | | | | | | | | | | | | | | | | | 0 | | | | | | | | | | | | | | | | | | | | | | | 0.0 | | | | | | | | | | | | | | | | | | 0.764 | | | | | | | | | | | |
|  | | | | | | | | | | | | | | | | | | | | | | | | | | | | | | | | | | | | | | | | | | | | | Never | | | | | | | | | | | | | | | | | | | | | | 11 | | | | | | | | | | | | | | | | | | | | | | | | | | 91.7 | | | | | | | | | | | | | | | | | | | | 1 | | | | | | | | | | | | | | | | | | | | | | | 100.0 | | | | | | | | | | | | | | | | | |  | | | | | | | | | | | |
| Nb of nights outside village for herding | | | | | | | | | | | | | | | | | | | | | | | | | | | | | | | | | | | | | | | | | | | | | | | | | | | | | Median (IQR) | | | | | | | | | | | | | | | | | | | | | | | | | | | | 7.5 | | | | | | | | | | | | | | | (2,25) | | | | | | | | | | | 2 | | | | | | | | | | | (2,2) | | | | | | | | | | | | | | | | | | 0.420 | | | | | | | | | | | | | | | | | | | | | | | | | | | | | |
|  | | | | | | | | | | | | | | | | | | | | | | | | | | | | | | | | | | | | | | | | | | | | | | | | | | | | | | | | | | | | | | | | | | | | | | | | | | | | | | | | | | | | | | | | | | | | | | | | | | | | | | | | | | | | | | | | | | | | | | | | | | | | | | | | | | | | | | | | | | | | | | | | | | | | | | | | | | | | | | | | | | | | | |
|  | | | | | | | | | | | | | | | | | | | | | | | | | | | | | | | | | | | | | | | | | | | | | | | | | | | | | | | | | | | | | | | | | | | | | | | | | | | | | | | | | | | | | | | | | | | | | | | | | | | | | | | | | | | | | | | | | | | | | | | | | | | | | | | | | | | | | | | | | | | | | | | | | | | | | | | | | | | | | | | | | | | | | |
|  | | | | | | | | | | | | | | | | | | | | | | | | | | | | | | | | | | | | | | | | | | | | | | | | | | | | | | | | | | | | | | | | | | | | | | | | | | | | | | | | | | | | | | | | | | | | | | | | | | | | | | | | | | | | | | | | | | | | | | | | | | | | | | | | | | | | | | | | | | | | | | | | | | | | | | | | | | | | | | | | | | | | | |
| **Use of repellents for the participant in the rainy season** | | | | | | | | | | | | | | | | | | | | | | | | | | | | | | | | | | | | | | | | | | | | | | | | | | | | | | | | | | | | | | | | | | | | | | | | | | | | | | | | | | | | | | | | | | | | | | | | | | | | | | | | | | | | | | | | | | | | | | | | | | | | | | | | | | | | | | | | | | | | | | | | | | | | | | | | | | | | | | | | | | | | | |
| Chemical insect repellent | | | | | | | | | | | | | | | | | | | | | | | | | | | | | | | | | | | | | | Daily | | | | | | | | | | | | | | | | | | | | | | | | | | | | | 7 | | | | | | | | | | | | | | | | | | | | | | | | | | 0.9 | | | | | | | | | | | | | | | | | | | | 2 | | | | | | | | | | | | | | | | | | | | | | | 1.0 | | | | | | | | | | | | | | | | | | 0.960 | | | | | | | | | | | |
| (2 missing) | | | | | | | | | | | | | | | | | | | | | | | | | | | | | | | | | | | | | | Frequently | | | | | | | | | | | | | | | | | | | | | | | | | | | | | 7 | | | | | | | | | | | | | | | | | | | | | | | | | | 0.9 | | | | | | | | | | | | | | | | | | | | 2 | | | | | | | | | | | | | | | | | | | | | | | 1.0 | | | | | | | | | | | | | | | | | |  | | | | | | | | | | | |
|  | | | | | | | | | | | | | | | | | | | | | | | | | | | | | | | | | | | | | | Sometimes | | | | | | | | | | | | | | | | | | | | | | | | | | | | | 11 | | | | | | | | | | | | | | | | | | | | | | | | | | 1.4 | | | | | | | | | | | | | | | | | | | | 2 | | | | | | | | | | | | | | | | | | | | | | | 1.0 | | | | | | | | | | | | | | | | | |  | | | | | | | | | | | |
|  | | | | | | | | | | | | | | | | | | | | | | | | | | | | | | | | | | | | | | Rarely | | | | | | | | | | | | | | | | | | | | | | | | | | | | | 1 | | | | | | | | | | | | | | | | | | | | | | | | | | 0.1 | | | | | | | | | | | | | | | | | | | | 0 | | | | | | | | | | | | | | | | | | | | | | | 0.0 | | | | | | | | | | | | | | | | | |  | | | | | | | | | | | |
|  | | | | | | | | | | | | | | | | | | | | | | | | | | | | | | | | | | | | | | Never | | | | | | | | | | | | | | | | | | | | | | | | | | | | | 773 | | | | | | | | | | | | | | | | | | | | | | | | | | 96.8 | | | | | | | | | | | | | | | | | | | | 192 | | | | | | | | | | | | | | | | | | | | | | | 97.0 | | | | | | | | | | | | | | | | | |  | | | | | | | | | | | |
| Natural oil or other body products | | | | | | | | | | | | | | | | | | | | | | | | | | | | | | | | | | | | | | | | | | | | | | Daily | | | | | | | | | | | | | | | | | | | | | 281 | | | | | | | | | | | | | | | | | | | | | | | | | | 35.2 | | | | | | | | | | | | | | | | | | | | 81 | | | | | | | | | | | | | | | | | | | | | | | 40.9 | | | | | | | | | | | | | | | | | | 0.007 | | | | | | | | | | | |
| (2 missing) | | | | | | | | | | | | | | | | | | | | | | | | | | | | | | | | | | | | | | | | | | | | | | Frequently | | | | | | | | | | | | | | | | | | | | | 26 | | | | | | | | | | | | | | | | | | | | | | | | | | 3.3 | | | | | | | | | | | | | | | | | | | | 14 | | | | | | | | | | | | | | | | | | | | | | | 7.1 | | | | | | | | | | | | | | | | | |  | | | | | | | | | | | |
|  | | | | | | | | | | | | | | | | | | | | | | | | | | | | | | | | | | | | | | | | | | | | | | Sometimes | | | | | | | | | | | | | | | | | | | | | 193 | | | | | | | | | | | | | | | | | | | | | | | | | | 24.2 | | | | | | | | | | | | | | | | | | | | 44 | | | | | | | | | | | | | | | | | | | | | | | 22.2 | | | | | | | | | | | | | | | | | |  | | | | | | | | | | | |
|  | | | | | | | | | | | | | | | | | | | | | | | | | | | | | | | | | | | | | | | | | | | | | | Rarely | | | | | | | | | | | | | | | | | | | | | 8 | | | | | | | | | | | | | | | | | | | | | | | | | | 1.0 | | | | | | | | | | | | | | | | | | | | 5 | | | | | | | | | | | | | | | | | | | | | | | 2.5 | | | | | | | | | | | | | | | | | |  | | | | | | | | | | | |
|  | | | | | | | | | | | | | | | | | | | | | | | | | | | | | | | | | | | | | | | | | | | | | | Never | | | | | | | | | | | | | | | | | | | | | 291 | | | | | | | | | | | | | | | | | | | | | | | | | | 36.4 | | | | | | | | | | | | | | | | | | | | 54 | | | | | | | | | | | | | | | | | | | | | | | 27.3 | | | | | | | | | | | | | | | | | |  | | | | | | | | | | | |
| Type of oil/product | | | | | | | | | | | | | | | | | | | | | | | | | | | | | | | Sesame oil | | | | | | | | | | | | | | | | | | | | | | | | | | | | | | | | | | | | 182 | | | | | | | | | | | | | | | | | | | | | | | | | | 22.7 | | | | | | | | | | | | | | | | | | | | 57 | | | | | | | | | | | | | | | | | | | | | | | 28.8 | | | | | | | | | | | | | | | | | | 0.073 | | | | | | | | | | | |
|  | | | | | | | | | | | | | | | | | | | | | | | | | | | | | | | Ground nut / beans oil | | | | | | | | | | | | | | | | | | | | | | | | | | | | | | | | | | | | 335 | | | | | | | | | | | | | | | | | | | | | | | | | | 41.8 | | | | | | | | | | | | | | | | | | | | 88 | | | | | | | | | | | | | | | | | | | | | | | 44.4 | | | | | | | | | | | | | | | | | | 0.504 | | | | | | | | | | | |
|  | | | | | | | | | | | | | | | | | | | | | | | | | | | | | | | Gasoline | | | | | | | | | | | | | | | | | | | | | | | | | | | | | | | | | | | | 6 | | | | | | | | | | | | | | | | | | | | | | | | | | 0.8 | | | | | | | | | | | | | | | | | | | | 4 | | | | | | | | | | | | | | | | | | | | | | | 2.0 | | | | | | | | | | | | | | | | | | 0.117 | | | | | | | | | | | |
|  | | | | | | | | | | | | | | | | | | | | | | | | | | | | | | | Other oil | | | | | | | | | | | | | | | | | | | | | | | | | | | | | | | | | | | | 4 | | | | | | | | | | | | | | | | | | | | | | | | | | 0.5 | | | | | | | | | | | | | | | | | | | | 6 | | | | | | | | | | | | | | | | | | | | | | | 3.0 | | | | | | | | | | | | | | | | | | 0.006 | | | | | | | | | | | |
| Thematic section (individual level): Activities and sleeping habits in the dry season | | | | | | | | | | | | | | | | | | | | | | | | | | | | | | | | | | | | | | | | | | | | | | | | | | | | | | | | | | | | | | | | | | | | | | | | | | | | | | | | | | | | | | | | | | | | | | | | | | | | | | | | | | | | | | | | | | | | | | | | | | | | | | | | | | | | | | | | | | | | | | | | | | | | | | | | | | | | | | | | | | | | | |
| **Fetch surface water** | | | | | | | | | | | | | | | | | | | | | | | | | | | | | | Daily | | | | | | | | | | | | | | | | | | | | | | | | | | | | | | | | | | | | | 108 | | | | | | | | | | | | | | | | | | | | | | | | | | 13.5 | | | | | | | | | | | | | | | | | | | | 18 | | | | | | | | | | | | | | | | | | | | | | | 9.1 | | | | | | | | | | 0.337 | | | | | | | | | | | | | | | | | | | |
|  | | | | | | | | | | | | | | | | | | | | | | | | | | | | | | Frequently | | | | | | | | | | | | | | | | | | | | | | | | | | | | | | | | | | | | | 49 | | | | | | | | | | | | | | | | | | | | | | | | | | 6.1 | | | | | | | | | | | | | | | | | | | | 10 | | | | | | | | | | | | | | | | | | | | | | | 5.1 | | | | | | | | | |  | | | | | | | | | | | | | | | | | | | |
|  | | | | | | | | | | | | | | | | | | | | | | | | | | | | | | Sometimes | | | | | | | | | | | | | | | | | | | | | | | | | | | | | | | | | | | | | 107 | | | | | | | | | | | | | | | | | | | | | | | | | | 13.4 | | | | | | | | | | | | | | | | | | | | 26 | | | | | | | | | | | | | | | | | | | | | | | 13.1 | | | | | | | | | |  | | | | | | | | | | | | | | | | | | | |
|  | | | | | | | | | | | | | | | | | | | | | | | | | | | | | | Rarely | | | | | | | | | | | | | | | | | | | | | | | | | | | | | | | | | | | | | 17 | | | | | | | | | | | | | | | | | | | | | | | | | | 2.1 | | | | | | | | | | | | | | | | | | | | 2 | | | | | | | | | | | | | | | | | | | | | | | 1.0 | | | | | | | | | |  | | | | | | | | | | | | | | | | | | | |
|  | | | | | | | | | | | | | | | | | | | | | | | | | | | | | | Never | | | | | | | | | | | | | | | | | | | | | | | | | | | | | | | | | | | | | 520 | | | | | | | | | | | | | | | | | | | | | | | | | | 64.9 | | | | | | | | | | | | | | | | | | | | 142 | | | | | | | | | | | | | | | | | | | | | | | 71.7 | | | | | | | | | |  | | | | | | | | | | | | | | | | | | | |
| **Activities in the forest** | | | | | | | | | | | | | | | | | | | | | | | | | | | | | | | | | | | Never | | | | | | | | | | | | | | | | | | | | | | | | | | | | | | | | 482 | | | | | | | | | | | | | | | | | | | | | | | | | | 60.3 | | | | | | | | | | | | | | | | | | | | 149 | | | | | | | | | | | | | | | | | | | | | | | 75.3 | | | | | | | | | | <0.001 | | | | | | | | | | | | | | | | | | | |
| **How frequently** | | | | | | | | | | | | | | | | | | | | | | | | | | | | | | | | | | Daily | | | | | | | | | | | | | | | | | | | | | | | | | | | | | | | | | | | 42 | | | | | | | | | | | | | | | | | | | | | | | | | 13.3 | | | | | | | | | | | | | | | | | | | | | 2 | | | | | | | | | | | | | | | | | | | | | | | | 4.1 | | | | | | | 0.286 | | | | | | | | | | | | | | | | | | | |
| (n=366) | | | | | | | | | | | | | | | | | | | | | | | | | | | | | | | | | | Frequently | | | | | | | | | | | | | | | | | | | | | | | | | | | | | | | | | | | 77 | | | | | | | | | | | | | | | | | | | | | | | | | 24.3 | | | | | | | | | | | | | | | | | | | | | 15 | | | | | | | | | | | | | | | | | | | | | | | | 30.6 | | | | | | |  | | | | | | | | | | | | | | | | | | | |
|  | | | | | | | | | | | | | | | | | | | | | | | | | | | | | | | | | | Sometimes | | | | | | | | | | | | | | | | | | | | | | | | | | | | | | | | | | | 165 | | | | | | | | | | | | | | | | | | | | | | | | | 52.1 | | | | | | | | | | | | | | | | | | | | | 26 | | | | | | | | | | | | | | | | | | | | | | | | 53.1 | | | | | | |  | | | | | | | | | | | | | | | | | | | |
|  | | | | | | | | | | | | | | | | | | | | | | | | | | | | | | | | | | Rarely | | | | | | | | | | | | | | | | | | | | | | | | | | | | | | | | | | | 33 | | | | | | | | | | | | | | | | | | | | | | | | | 10.4 | | | | | | | | | | | | | | | | | | | | | 6 | | | | | | | | | | | | | | | | | | | | | | | | 12.2 | | | | | | |  | | | | | | | | | | | | | | | | | | | |
| **Naps during the day** | | | | | | | | | | | | | | | | | | | | | | | | | | | | | | | | | | | Daily | | | | | | | | | | | | | | | | | | | | | | | | | | | | | | | | 132 | | | | | | | | | | | | | | | | | | | | | | | | | | 16.5 | | | | | | | | | | | | | | | | | | | | 32 | | | | | | | | | | | | | | | | | | | | | | | 16.2 | | | | | | | | | | 0.885 | | | | | | | | | | | | | | | | | | | |
|  | | | | | | | | | | | | | | | | | | | | | | | | | | | | | | | | | | | Frequently | | | | | | | | | | | | | | | | | | | | | | | | | | | | | | | | 38 | | | | | | | | | | | | | | | | | | | | | | | | | | 4.7 | | | | | | | | | | | | | | | | | | | | 8 | | | | | | | | | | | | | | | | | | | | | | | 4.0 | | | | | | | | | |  | | | | | | | | | | | | | | | | | | | |
|  | | | | | | | | | | | | | | | | | | | | | | | | | | | | | | | | | | | Sometimes | | | | | | | | | | | | | | | | | | | | | | | | | | | | | | | | 343 | | | | | | | | | | | | | | | | | | | | | | | | | | 42.8 | | | | | | | | | | | | | | | | | | | | 82 | | | | | | | | | | | | | | | | | | | | | | | 41.4 | | | | | | | | | |  | | | | | | | | | | | | | | | | | | | |
|  | | | | | | | | | | | | | | | | | | | | | | | | | | | | | | | | | | | Rarely | | | | | | | | | | | | | | | | | | | | | | | | | | | | | | | | 39 | | | | | | | | | | | | | | | | | | | | | | | | | | 4.9 | | | | | | | | | | | | | | | | | | | | 13 | | | | | | | | | | | | | | | | | | | | | | | 6.6 | | | | | | | | | |  | | | | | | | | | | | | | | | | | | | |
|  | | | | | | | | | | | | | | | | | | | | | | | | | | | | | | | | | | | Never | | | | | | | | | | | | | | | | | | | | | | | | | | | | | | | | 249 | | | | | | | | | | | | | | | | | | | | | | | | | | 31.1 | | | | | | | | | | | | | | | | | | | | 63 | | | | | | | | | | | | | | | | | | | | | | | 31.8 | | | | | | | | | |  | | | | | | | | | | | | | | | | | | | |
| If yes, naps under a tree | | | | | | | | | | | | | | | | | | | | | | | | | | | | | | | | | | | Daily | | | | | | | | | | | | | | | | | | | | | | | | | | | | | | | | 16 | | | | | | | | | | | | | | | | | | | | | | | | | | 2.9 | | | | | | | | | | | | | | | | | | | | 4 | | | | | | | | | | | | | | | | | | | | | | | 3.0 | | | | | | | | | | 0.881 | | | | | | | | | | | | | | | | | | | |
|  | | | | | | | | | | | | | | | | | | | | | | | | | | | | | | | | | | | Frequently | | | | | | | | | | | | | | | | | | | | | | | | | | | | | | | | 11 | | | | | | | | | | | | | | | | | | | | | | | | | | 2.0 | | | | | | | | | | | | | | | | | | | | 4 | | | | | | | | | | | | | | | | | | | | | | | 3.0 | | | | | | | | | |  | | | | | | | | | | | | | | | | | | | |
|  | | | | | | | | | | | | | | | | | | | | | | | | | | | | | | | | | | | Sometimes | | | | | | | | | | | | | | | | | | | | | | | | | | | | | | | | 118 | | | | | | | | | | | | | | | | | | | | | | | | | | 21.4 | | | | | | | | | | | | | | | | | | | | 27 | | | | | | | | | | | | | | | | | | | | | | | 20.0 | | | | | | | | | |  | | | | | | | | | | | | | | | | | | | |
|  | | | | | | | | | | | | | | | | | | | | | | | | | | | | | | | | | | | Rarely | | | | | | | | | | | | | | | | | | | | | | | | | | | | | | | | 9 | | | | | | | | | | | | | | | | | | | | | | | | | | 1.6 | | | | | | | | | | | | | | | | | | | | 3 | | | | | | | | | | | | | | | | | | | | | | | 2.2 | | | | | | | | | |  | | | | | | | | | | | | | | | | | | | |
|  | | | | | | | | | | | | | | | | | | | | | | | | | | | | | | | | | | | Never | | | | | | | | | | | | | | | | | | | | | | | | | | | | | | | | 398 | | | | | | | | | | | | | | | | | | | | | | | | | | 72.1 | | | | | | | | | | | | | | | | | | | | 97 | | | | | | | | | | | | | | | | | | | | | | | 71.9 | | | | | | | | | |  | | | | | | | | | | | | | | | | | | | |
| If yes, type | | | | | | | Neem (*Azadirachta indica*) | | | | | | | | | | | | | | | | | | | | | | | | | | | | | | | | | | | | | | | | | | | | | | | | | | | | | | | | | | | | 102 | | | | | | | | | | | | | | | | | | | | | | | | | | 66.2 | | | | | | | | | | | | | | | | | | | | 21 | | | | | | | | | | | | | | | | | | | | | | | 55.3 | | | | | | | | | | 0.207 | | | | | | | | | | | | | | | | | | | |
| of tree | | | | | | | Lalob/ higleeg (*Balanites aegyptica*) | | | | | | | | | | | | | | | | | | | | | | | | | | | | | | | | | | | | | | | | | | | | | | | | | | | | | | | | | | | | 40 | | | | | | | | | | | | | | | | | | | | | | | | | | 26.0 | | | | | | | | | | | | | | | | | | | | 11 | | | | | | | | | | | | | | | | | | | | | | | 29.0 | | | | | | | | | | 0.710 | | | | | | | | | | | | | | | | | | | |
| (n=192) | | | | | | | Sidr (*Ziziphus spina-christi*) | | | | | | | | | | | | | | | | | | | | | | | | | | | | | | | | | | | | | | | | | | | | | | | | | | | | | | | | | | | | 25 | | | | | | | | | | | | | | | | | | | | | | | | | | 16.2 | | | | | | | | | | | | | | | | | | | | 9 | | | | | | | | | | | | | | | | | | | | | | | 23.7 | | | | | | | | | | 0.281 | | | | | | | | | | | | | | | | | | | |
|  | | | | | | | Hashab (*Acacia senegal*) | | | | | | | | | | | | | | | | | | | | | | | | | | | | | | | | | | | | | | | | | | | | | | | | | | | | | | | | | | | | 5 | | | | | | | | | | | | | | | | | | | | | | | | | | 3.3 | | | | | | | | | | | | | | | | | | | | 4 | | | | | | | | | | | | | | | | | | | | | | | 10.5 | | | | | | | | | | 0.078 | | | | | | | | | | | | | | | | | | | |
|  | | | | | | | Sonot/Garat (*Acacia nilotica*) | | | | | | | | | | | | | | | | | | | | | | | | | | | | | | | | | | | | | | | | | | | | | | | | | | | | | | | | | | | | 11 | | | | | | | | | | | | | | | | | | | | | | | | | | 7.1 | | | | | | | | | | | | | | | | | | | | 4 | | | | | | | | | | | | | | | | | | | | | | | 10.5 | | | | | | | | | | 0.502 | | | | | | | | | | | | | | | | | | | |
|  | | | | | | | Taleh (*Acacia seyal*) | | | | | | | | | | | | | | | | | | | | | | | | | | | | | | | | | | | | | | | | | | | | | | | | | | | | | | | | | | | | 4 | | | | | | | | | | | | | | | | | | | | | | | | | | 2.6 | | | | | | | | | | | | | | | | | | | | 2 | | | | | | | | | | | | | | | | | | | | | | | 5.3 | | | | | | | | | | 0.339 | | | | | | | | | | | | | | | | | | | |
|  | | | | | | | Kiter (*Acacia Mellifera*) | | | | | | | | | | | | | | | | | | | | | | | | | | | | | | | | | | | | | | | | | | | | | | | | | | | | | | | | | | | | 0 | | | | | | | | | | | | | | | | | | | | | | | | | | 0.0 | | | | | | | | | | | | | | | | | | | | 1 | | | | | | | | | | | | | | | | | | | | | | | 2.6 | | | | | | | | | | 0.198 | | | | | | | | | | | | | | | | | | | |
| **Time when goes to sleep** | | | | | | | | | | | | | | | | | | | | | | | | | | | | | | | | | | | | | Before sunset | | | | | | | | | | | | | | | | | | | | | | | | | | | | | | 5 | | | | | | | | | | | | | | | | | | | | | | | | | | 0.6 | | | | | | | | | | | | | | | | | | | | 0 | | | | | | | | | | | | | | | | | | | | | | | 0.0 | | | | | | | | | | 0.365 | | | | | | | | | | | | | | | | | | | |
| (1 don’t know) | | | | | | | | | | | | | | | | | | | | | | | | | | | | | | | | | | | | | At sunset | | | | | | | | | | | | | | | | | | | | | | | | | | | | | | 23 | | | | | | | | | | | | | | | | | | | | | | | | | | 2.9 | | | | | | | | | | | | | | | | | | | | 7 | | | | | | | | | | | | | | | | | | | | | | | 3.5 | | | | | | | | | |  | | | | | | | | | | | | | | | | | | | |
|  | | | | | | | | | | | | | | | | | | | | | | | | | | | | | | | | | | | | | After sunset | | | | | | | | | | | | | | | | | | | | | | | | | | | | | | 748 | | | | | | | | | | | | | | | | | | | | | | | | | | 93.4 | | | | | | | | | | | | | | | | | | | | 188 | | | | | | | | | | | | | | | | | | | | | | | 95.0 | | | | | | | | | |  | | | | | | | | | | | | | | | | | | | |
|  | | | | | | | | | | | | | | | | | | | | | | | | | | | | | | | | | | | | | Very variable | | | | | | | | | | | | | | | | | | | | | | | | | | | | | | 10 | | | | | | | | | | | | | | | | | | | | | | | | | | 1.3 | | | | | | | | | | | | | | | | | | | | 3 | | | | | | | | | | | | | | | | | | | | | | | 1.5 | | | | | | | | | |  | | | | | | | | | | | | | | | | | | | |
|  | | | | | | | | | | | | | | | | | | | | | | | | | | | | | | | | | | | | | Not relevant | | | | | | | | | | | | | | | | | | | | | | | | | | | | | | 14 | | | | | | | | | | | | | | | | | | | | | | | | | | 1.8 | | | | | | | | | | | | | | | | | | | | 0 | | | | | | | | | | | | | | | | | | | | | | | 0.0 | | | | | | | | | |  | | | | | | | | | | | | | | | | | | | |
| **Bedtime hour** | | | | | | | | | | | | | | | | | | | | | | | | | | | | | | | | | | | | | | | | | | | | | | 17 to 18h | | | | | | | | | | | | | | | | | | | | | 23 | | | | | | | | | | | | | | | | | | | | | | | | | | 2.9 | | | | | | | | | | | | | | | | | | | | 7 | | | | | | | | | | | | | | | | | | | | | | | 3.6 | | | | | | | | | | 0.003 | | | | | | | | | | | | | | | | | | | |
|  | | | | | | | | | | | | | | | | | | | | | | | | | | | | | | | | | | | | | | | | | | | | | | 19 to 20h | | | | | | | | | | | | | | | | | | | | | 137 | | | | | | | | | | | | | | | | | | | | | | | | | | 17.5 | | | | | | | | | | | | | | | | | | | | 40 | | | | | | | | | | | | | | | | | | | | | | | 20.6 | | | | | | | | | |  | | | | | | | | | | | | | | | | | | | |
|  | | | | | | | | | | | | | | | | | | | | | | | | | | | | | | | | | | | | | | | | | | | | | | 21 to 22h | | | | | | | | | | | | | | | | | | | | | 368 | | | | | | | | | | | | | | | | | | | | | | | | | | 46.9 | | | | | | | | | | | | | | | | | | | | 112 | | | | | | | | | | | | | | | | | | | | | | | 57.7 | | | | | | | | | |  | | | | | | | | | | | | | | | | | | | |
|  | | | | | | | | | | | | | | | | | | | | | | | | | | | | | | | | | | | | | | | | | | | | | | 23h to 2h | | | | | | | | | | | | | | | | | | | | | 256 | | | | | | | | | | | | | | | | | | | | | | | | | | 32.6 | | | | | | | | | | | | | | | | | | | | 35 | | | | | | | | | | | | | | | | | | | | | | | 18.0 | | | | | | | | | |  | | | | | | | | | | | | | | | | | | | |
| **Goes out before sunrise** | | | | | | | | | | | | | | | | | | | | | | | | | | | | | | | | | | | | | | | | | | | | | | Daily | | | | | | | | | | | | | | | | | | | | | 29 | | | | | | | | | | | | | | | | | | | | | | | | | | 13.1 | | | | | | | | | | | | | | | | | | | | 5 | | | | | | | | | | | | | | | | | | | | | | | 11.1 | | | | | | | | | | 0.935 | | | | | | | | | | | | | | | | | | | |
| (age ≥1 and sleeping inside) | | | | | | | | | | | | | | | | | | | | | | | | | | | | | | | | | | | | | | | | | | | | | | Frequently | | | | | | | | | | | | | | | | | | | | | 10 | | | | | | | | | | | | | | | | | | | | | | | | | | 4.5 | | | | | | | | | | | | | | | | | | | | 1 | | | | | | | | | | | | | | | | | | | | | | | 2.2 | | | | | | | | | |  | | | | | | | | | | | | | | | | | | | |
|  | | | | | | | | | | | | | | | | | | | | | | | | | | | | | | | | | | | | | | | | | | | | | | Sometimes | | | | | | | | | | | | | | | | | | | | | 70 | | | | | | | | | | | | | | | | | | | | | | | | | | 31.5 | | | | | | | | | | | | | | | | | | | | 17 | | | | | | | | | | | | | | | | | | | | | | | 37.8 | | | | | | | | | |  | | | | | | | | | | | | | | | | | | | |
|  | | | | | | | | | | | | | | | | | | | | | | | | | | | | | | | | | | | | | | | | | | | | | | Rarely | | | | | | | | | | | | | | | | | | | | | 5 | | | | | | | | | | | | | | | | | | | | | | | | | | 2.3 | | | | | | | | | | | | | | | | | | | | 1 | | | | | | | | | | | | | | | | | | | | | | | 2.2 | | | | | | | | | |  | | | | | | | | | | | | | | | | | | | |
|  | | | | | | | | | | | | | | | | | | | | | | | | | | | | | | | | | | | | | | | | | | | | | | Never | | | | | | | | | | | | | | | | | | | | | 108 | | | | | | | | | | | | | | | | | | | | | | | | | | 48.7 | | | | | | | | | | | | | | | | | | | | 21 | | | | | | | | | | | | | | | | | | | | | | | 46.7 | | | | | | | | | |  | | | | | | | | | | | | | | | | | | | |
| **Place of stay between sunset and sleep** (35 going to bed before or at sunset excluded) | | | | | | | | | | | | | | | | | | | | | | | | | | | | | | | | | | | | | | | | | | | | | | | | | | | | | | | | | | | | | | | | | | | | | | | | | | | | | | | | | | | | | | | | | | | | | | | | | | | | | | | | | | | | | | | | | | | | | | | | | | | | | | | | | | | | | | | | | | | | | | | | | | 0.571 | | | | | | | | | | | | | | | | | | | |
|  | | | | | | | | | Indoor only | | | | | | | | | | | | | | | | | | | | | | | | | | | | | | | | | | | | | | | | | | | | | | | | | | | | | | | | | | 19 | | | | | | | | | | | | | | | | | | | | | | | | | | 2.5 | | | | | | | | | | | | | | | | | | | | 7 | | | | | | | | | | | | | | | | | | | | | | | 3.7 | | | | | | | | | |  | | | | | | | | | | | | | | | | | | | |
|  | | | | | | | | | Outdoor | | | | | | | | | | | | | | | | | | | | | | | | | | | | | | | | | | | | | | | | | | | | | | | | | | | | | | | | | | 731 | | | | | | | | | | | | | | | | | | | | | | | | | | 94.5 | | | | | | | | | | | | | | | | | | | | 177 | | | | | | | | | | | | | | | | | | | | | | | 92.7 | | | | | | | | | |  | | | | | | | | | | | | | | | | | | | |
|  | | | | | | | | | Both indoor and outdoor | | | | | | | | | | | | | | | | | | | | | | | | | | | | | | | | | | | | | | | | | | | | | | | | | | | | | | | | | | 23 | | | | | | | | | | | | | | | | | | | | | | | | | | 3.0 | | | | | | | | | | | | | | | | | | | | 7 | | | | | | | | | | | | | | | | | | | | | | | 3.7 | | | | | | | | | |  | | | | | | | | | | | | | | | | | | | |
| **Main outdoor activities after sunset** (among participants staying outdoor after sunset) | | | | | | | | | | | | | | | | | | | | | | | | | | | | | | | | | | | | | | | | | | | | | | | | | | | | | | | | | | | | | | | | | | | | | | | | | | | | | | | | | | | | | | | | | | | | | | | | | | | | | | | | | | | | | | | | | | | | | | | | | | | | | | | | | | | | | | | | | | | | | | | | | | <0.001 | | | | | | | | | | | | | | | | | | | |
|  | | | | | | | | | Farming / herding animals | | | | | | | | | | | | | | | | | | | | | | | | | | | | | | | | | | | | | | | | | | | | | | | | | | | | | | | | | | 3 | | | | | | | | | | | | | | | | | | | | | | | | | | 0.4 | | | | | | | | | | | | | | | | | | | | 1 | | | | | | | | | | | | | | | | | | | | | | | 0.5 | | | | | | | | | |  | | | | | | | | | | | | | | | | | | | |
|  | | | | | | | | | Playing | | | | | | | | | | | | | | | | | | | | | | | | | | | | | | | | | | | | | | | | | | | | | | | | | | | | | | | | | | 219 | | | | | | | | | | | | | | | | | | | | | | | | | | 29.0 | | | | | | | | | | | | | | | | | | | | 81 | | | | | | | | | | | | | | | | | | | | | | | 44.0 | | | | | | | | | |  | | | | | | | | | | | | | | | | | | | |
|  | | | | | | | | | TV / radio | | | | | | | | | | | | | | | | | | | | | | | | | | | | | | | | | | | | | | | | | | | | | | | | | | | | | | | | | | 109 | | | | | | | | | | | | | | | | | | | | | | | | | | 14.5 | | | | | | | | | | | | | | | | | | | | 38 | | | | | | | | | | | | | | | | | | | | | | | 20.6 | | | | | | | | | |  | | | | | | | | | | | | | | | | | | | |
|  | | | | | | | | | Discussing-relaxing | | | | | | | | | | | | | | | | | | | | | | | | | | | | | | | | | | | | | | | | | | | | | | | | | | | | | | | | | | 318 | | | | | | | | | | | | | | | | | | | | | | | | | | 42.2 | | | | | | | | | | | | | | | | | | | | 53 | | | | | | | | | | | | | | | | | | | | | | | 28.8 | | | | | | | | | |  | | | | | | | | | | | | | | | | | | | |
|  | | | | | | | | | Selling at the market | | | | | | | | | | | | | | | | | | | | | | | | | | | | | | | | | | | | | | | | | | | | | | | | | | | | | | | | | | 13 | | | | | | | | | | | | | | | | | | | | | | | | | | 1.7 | | | | | | | | | | | | | | | | | | | | 3 | | | | | | | | | | | | | | | | | | | | | | | 1.6 | | | | | | | | | |  | | | | | | | | | | | | | | | | | | | |
|  | | | | | | | | | Cooking/house activities | | | | | | | | | | | | | | | | | | | | | | | | | | | | | | | | | | | | | | | | | | | | | | | | | | | | | | | | | | 62 | | | | | | | | | | | | | | | | | | | | | | | | | | 8.2 | | | | | | | | | | | | | | | | | | | | 4 | | | | | | | | | | | | | | | | | | | | | | | 2.2 | | | | | | | | | |  | | | | | | | | | | | | | | | | | | | |
|  | | | | | | | | | Reading/lessons | | | | | | | | | | | | | | | | | | | | | | | | | | | | | | | | | | | | | | | | | | | | | | | | | | | | | | | | | | 30 | | | | | | | | | | | | | | | | | | | | | | | | | | 4.0 | | | | | | | | | | | | | | | | | | | | 4 | | | | | | | | | | | | | | | | | | | | | | | 2.2 | | | | | | | | | |  | | | | | | | | | | | | | | | | | | | |
| **Location outside** (among participants reporting outdoor after sunset) | | | | | | | | | | | | | | | | | | | | | | | | | | | | | | | | | | | | | | | | | | | | | | | | | | | | | | | | | | | | | | | | | | | | | | | | | | | | | | | | | | | | | | | | | | | | | | | | | | | | | | | | | | | | | | | | | | | | | | | | | | | | | | | | | | | | | | | | | | | | | | | | | | 0.454 | | | | | | | | | | | | | | | | | | | |
|  | | | | | House yard | | | | | | | | | | | | | | | | | | | | | | | | | | | | | | | | | | | | | | | | | | | | | | | | | | | | | | | | | | | | | | 555 | | | | | | | | | | | | | | | | | | | | | | | | | | 73.6 | | | | | | | | | | | | | | | | | | | | 130 | | | | | | | | | | | | | | | | | | | | | | | 70.6 | | | | | | | | | |  | | | | | | | | | | | | | | | | | | | |
|  | | | | | Market | | | | | | | | | | | | | | | | | | | | | | | | | | | | | | | | | | | | | | | | | | | | | | | | | | | | | | | | | | | | | | 53 | | | | | | | | | | | | | | | | | | | | | | | | | | 7.0 | | | | | | | | | | | | | | | | | | | | 15 | | | | | | | | | | | | | | | | | | | | | | | 8.1 | | | | | | | | | |  | | | | | | | | | | | | | | | | | | | |
|  | | | | | Fields | | | | | | | | | | | | | | | | | | | | | | | | | | | | | | | | | | | | | | | | | | | | | | | | | | | | | | | | | | | | | | 1 | | | | | | | | | | | | | | | | | | | | | | | | | | 0.1 | | | | | | | | | | | | | | | | | | | | 0 | | | | | | | | | | | | | | | | | | | | | | | 0.0 | | | | | | | | | |  | | | | | | | | | | | | | | | | | | | |
|  | | | | | Next to house yard | | | | | | | | | | | | | | | | | | | | | | | | | | | | | | | | | | | | | | | | | | | | | | | | | | | | | | | | | | | | | | 109 | | | | | | | | | | | | | | | | | | | | | | | | | | 14.5 | | | | | | | | | | | | | | | | | | | | 26 | | | | | | | | | | | | | | | | | | | | | | | 14.1 | | | | | | | | | |  | | | | | | | | | | | | | | | | | | | |
|  | | | | | In the village, other | | | | | | | | | | | | | | | | | | | | | | | | | | | | | | | | | | | | | | | | | | | | | | | | | | | | | | | | | | | | | | 35 | | | | | | | | | | | | | | | | | | | | | | | | | | 4.6 | | | | | | | | | | | | | | | | | | | | 12 | | | | | | | | | | | | | | | | | | | | | | | 6.5 | | | | | | | | | |  | | | | | | | | | | | | | | | | | | | |
|  | | | | | In a neighbouring village | | | | | | | | | | | | | | | | | | | | | | | | | | | | | | | | | | | | | | | | | | | | | | | | | | | | | | | | | | | | | | 0 | | | | | | | | | | | | | | | | | | | | | | | | | | 0.0 | | | | | | | | | | | | | | | | | | | | 1 | | | | | | | | | | | | | | | | | | | | | | | 0.5 | | | | | | | | | |  | | | | | | | | | | | | | | | | | | | |
| **Goes out early morning (sunrise)** | | | | | | | | | | | | | | | | | | | | | | | | | | | | | | | | | | | | | | | | | | | | | | Daily | | | | | | | | | | | | | | | | | | | | | 79 | | | | | | | | | | | | | | | | | | | | | | | | | | 35.6 | | | | | | | | | | | | | | | | | | | | 8 | | | | | | | | | | | | | | | | | | | | | | | 17.8 | | | | | | | | | | 0.055 | | | | | | | | | | | | | | | | | | | |
| (age ≥1 and sleeping inside) | | | | | | | | | | | | | | | | | | | | | | | | | | | | | | | | | | | | | | | | | | | | | | Frequently | | | | | | | | | | | | | | | | | | | | | 3 | | | | | | | | | | | | | | | | | | | | | | | | | | 1.4 | | | | | | | | | | | | | | | | | | | | 0 | | | | | | | | | | | | | | | | | | | | | | | 0.0 | | | | | | | | | |  | | | | | | | | | | | | | | | | | | | |
|  | | | | | | | | | | | | | | | | | | | | | | | | | | | | | | | | | | | | | | | | | | | | | | Sometimes | | | | | | | | | | | | | | | | | | | | | 47 | | | | | | | | | | | | | | | | | | | | | | | | | | 21.2 | | | | | | | | | | | | | | | | | | | | 9 | | | | | | | | | | | | | | | | | | | | | | | 20.0 | | | | | | | | | |  | | | | | | | | | | | | | | | | | | | |
|  | | | | | | | | | | | | | | | | | | | | | | | | | | | | | | | | | | | | | | | | | | | | | | Rarely | | | | | | | | | | | | | | | | | | | | | 5 | | | | | | | | | | | | | | | | | | | | | | | | | | 2.3 | | | | | | | | | | | | | | | | | | | | 0 | | | | | | | | | | | | | | | | | | | | | | | 0.0 | | | | | | | | | |  | | | | | | | | | | | | | | | | | | | |
|  | | | | | | | | | | | | | | | | | | | | | | | | | | | | | | | | | | | | | | | | | | | | | | Never | | | | | | | | | | | | | | | | | | | | | 88 | | | | | | | | | | | | | | | | | | | | | | | | | | 39.6 | | | | | | | | | | | | | | | | | | | | 28 | | | | | | | | | | | | | | | | | | | | | | | 62.2 | | | | | | | | | |  | | | | | | | | | | | | | | | | | | | |
| **Sleep location** | | | | | | | | | | | | In the house yard | | | | | | | | | | | | | | | | | | | | | | | | | | | | | | | | | | | | | | | | | | | | | | | | | | | | | | | 781 | | | | | | | | | | | | | | | | | | | | | | | | | | 97.5 | | | | | | | | | | | | | | | | | | | | 183 | | | | | | | | | | | | | | | | | | | | | | | 92.4 | | | | | | | | | | | | | | | | | | 0.002 | | | | | | | | | | | |
|  |  |  |  |  |  |  |  |  |  |  |  | In the village but not in the yard | | | | | | | | | | | | | | | | | | | | | | | | | | | | | | | | | | | | | | | | | | | | | | | | | | | | | | | 19 | | | | | | | | | | | | | | | | | | | | | | | | | | 2.4 | | | | | | | | | | | | | | | | | | | | 15 | | | | | | | | | | | | | | | | | | | | | | | 7.6 | | | | | | | | | | | | | | | | | |  | | | | | | | | | | | |
|  |  |  |  |  |  |  |  |  |  |  |  | In a neighbouring village | | | | | | | | | | | | | | | | | | | | | | | | | | | | | | | | | | | | | | | | | | | | | | | | | | | | | | | 1 | | | | | | | | | | | | | | | | | | | | | | | | | | 0.1 | | | | | | | | | | | | | | | | | | | | 0 | | | | | | | | | | | | | | | | | | | | | | | 0.0 | | | | | | | | | | | | | | | | | |  | | | | | | | | | | | |
|  |  |  |  |  |  |  |  |  |  |  |  | Farm/field | | | | | | | | | | | | | | | | | | | | | | | | | | | | | | | | | | | | | | | | | | | | | | | | | | | | | | | 0 | | | | | | | | | | | | | | | | | | | | | | | | | | 0.0 | | | | | | | | | | | | | | | | | | | | 0 | | | | | | | | | | | | | | | | | | | | | | | 0.0 | | | | | | | | | | | | | | | | | |  | | | | | | | | | | | |
| **Usually sleeping on** | | | | | | | | | | | | | | | | | | | | | | | | | | | | | | | | | a bed | | | | | | | | | | | | | | | | | | | | | | | | | | | | | | | | | | 793 | | | | | | | | | | | | | | | | | | | | | | | | | | 99.1 | | | | | | | | | | | | | | | | | | | | 195 | | | | | | | | | | | | | | | | | | | | | | | 98.4 | | | | | | | | | | | | | | | | | | 0.378 | | | | | | | | | | | |
| (1 missing) | | | | | | | | | | | | | | | | | | | | | | | | | | | | | | | | | the floor | | | | | | | | | | | | | | | | | | | | | | | | | | | | | | | | | | 6 | | | | | | | | | | | | | | | | | | | | | | | | | | 0.7 | | | | | | | | | | | | | | | | | | | | 2 | | | | | | | | | | | | | | | | | | | | | | | 1.0 | | | | | | | | | | | | | | | | | |  | | | | | | | | | | | |
|  | | | | | | | | | | | | | | | | | | | | | | | | | | | | | | | | | a mat or mattress | | | | | | | | | | | | | | | | | | | | | | | | | | | | | | | | | | 1 | | | | | | | | | | | | | | | | | | | | | | | | | | 0.1 | | | | | | | | | | | | | | | | | | | | 1 | | | | | | | | | | | | | | | | | | | | | | | 0.5 | | | | | | | | | | | | | | | | | |  | | | | | | | | | | | |
| **Place of sleep** | | | | | | | | | | | | | | | | | | | | | | | | | | | | | | In a brick/cement room | | | | | | | | | | | | | | | | | | | | | | | | | | | | | | | | | | | | | 2 | | | | | | | | | | | | | | | | | | | | | | | | | | 0.3 | | | | | | | | | | | | | | | | | | | | 1 | | | | | | | | | | | | | | | | | | | | | | | 0.5 | | | | | | | | | | | | | | | | | | 0.451 | | | | | | | | | | | |
|  | | | | | | | | | | | | | | | | | | | | | | | | | | | | | | In a tukul | | | | | | | | | | | | | | | | | | | | | | | | | | | | | | | | | | | | | 216 | | | | | | | | | | | | | | | | | | | | | | | | | | 27.0 | | | | | | | | | | | | | | | | | | | | 43 | | | | | | | | | | | | | | | | | | | | | | | 21.7 | | | | | | | | | | | | | | | | | |  | | | | | | | | | | | |
|  | | | | | | | | | | | | | | | | | | | | | | | | | | | | | | In a “local” room | | | | | | | | | | | | | | | | | | | | | | | | | | | | | | | | | | | | | 8 | | | | | | | | | | | | | | | | | | | | | | | | | | 1.0 | | | | | | | | | | | | | | | | | | | | 1 | | | | | | | | | | | | | | | | | | | | | | | 0.5 | | | | | | | | | | | | | | | | | |  | | | | | | | | | | | |
|  | | | | | | | | | | | | | | | | | | | | | | | | | | | | | | Under a shelter | | | | | | | | | | | | | | | | | | | | | | | | | | | | | | | | | | | | | 31 | | | | | | | | | | | | | | | | | | | | | | | | | | 3.9 | | | | | | | | | | | | | | | | | | | | 9 | | | | | | | | | | | | | | | | | | | | | | | 4.6 | | | | | | | | | | | | | | | | | |  | | | | | | | | | | | |
|  | | | | | | | | | | | | | | | | | | | | | | | | | | | | | | In an open space | | | | | | | | | | | | | | | | | | | | | | | | | | | | | | | | | | | | | 385 | | | | | | | | | | | | | | | | | | | | | | | | | | 48.1 | | | | | | | | | | | | | | | | | | | | 108 | | | | | | | | | | | | | | | | | | | | | | | 54.6 | | | | | | | | | | | | | | | | | |  | | | | | | | | | | | |
|  | | | | | | | | | | | | | | | | | | | | | | | | | | | | | | Variable | | | | | | | | | | | | | | | | | | | | | | | | | | | | | | | | | | | | | 159 | | | | | | | | | | | | | | | | | | | | | | | | | | 19.9 | | | | | | | | | | | | | | | | | | | | 36 | | | | | | | | | | | | | | | | | | | | | | | 18.2 | | | | | | | | | | | | | | | | | |  | | | | | | | | | | | |
| **Sleeping covered** | | | | | | | | | | | | | | | | | | | | | | | | | | | | | | Covered | | | | | | | | | | | | | | | | | | | | | | | | | | | | | | | | | | | | | 374 | | | | | | | | | | | | | | | | | | | | | | | | | | 46.7 | | | | | | | | | | | | | | | | | | | | 81 | | | | | | | | | | | | | | | | | | | | | | | 40.9 | | | | | | | | | | | | | | | | | | 0.279 | | | | | | | | | | | |
|  | | | | | | | | | | | | | | | | | | | | | | | | | | | | | | Not covered | | | | | | | | | | | | | | | | | | | | | | | | | | | | | | | | | | | | | 324 | | | | | | | | | | | | | | | | | | | | | | | | | | 40.5 | | | | | | | | | | | | | | | | | | | | 92 | | | | | | | | | | | | | | | | | | | | | | | 46.5 | | | | | | | | | | | | | | | | | |  | | | | | | | | | | | |
|  | | | | | | | | | | | | | | | | | | | | | | | | | | | | | | Variable | | | | | | | | | | | | | | | | | | | | | | | | | | | | | | | | | | | | | 103 | | | | | | | | | | | | | | | | | | | | | | | | | | 12.9 | | | | | | | | | | | | | | | | | | | | 25 | | | | | | | | | | | | | | | | | | | | | | | 12.6 | | | | | | | | | | | | | | | | | |  | | | | | | | | | | | |
| ***If covered, with*** | | | | | | | | | | | | | | | | | | | | | | | | | | | | | | | | | | | | | | | *Sheet* | | | | | | | | | | | | | | | | | | | | | | | | | | | | *441* | | | | | | | | | | | | | | | | | | | | | | | | | | *55.1* | | | | | | | | | | | | | | | | | | | | *94* | | | | | | | | | | | | | | | | | | | | | | | *47.5* | | | | | | | | | | | | | | | | | |  | | | | | | | | | | | |
|  | | | | | | | | | | | | | | | | | | | | | | | | | | | | | | | | | | | | | | | *Blanket* | | | | | | | | | | | | | | | | | | | | | | | | | | | | *23* | | | | | | | | | | | | | | | | | | | | | | | | | | *2.9* | | | | | | | | | | | | | | | | | | | | *10* | | | | | | | | | | | | | | | | | | | | | | | *5.1* | | | | | | | | | | | | | | | | | |  | | | | | | | | | | | |
|  | | | | | | | | | | | | | | | | | | | | | | | | | | | | | | | | | | | | | | | *Sheet or blanket* | | | | | | | | | | | | | | | | | | | | | | | | | | | | *13* | | | | | | | | | | | | | | | | | | | | | | | | | | *1.6* | | | | | | | | | | | | | | | | | | | | *2* | | | | | | | | | | | | | | | | | | | | | | | *1.0* | | | | | | | | | | | | | | | | | |  | | | | | | | | | | | |
| **Sleep under a mosquito-net** | | | | | | | | | | | | | | | | | | | | | | | | | | | | | | | | | | | | | | | | | | | | | | Never | | | | | | | | | | | | | | | | | | | | | 576 | | | | | | | | | | | | | | | | | | | | | | | | | | 71.9 | | | | | | | | | | | | | | | | | | | | 143 | | | | | | | | | | | | | | | | | | | | | | | 72.2 | | | | | | | | | | | | | | | | | | 0.930 | | | | | | | | | | | |
| **Frequency of mosquito-net use** | | | | | | | | | | | | | | | | | | | | | | | | | | | | | | | | | | | | | | | | | | | | | | Daily | | | | | | | | | | | | | | | | | | | | | 193 | | | | | | | | | | | | | | | | | | | | | | | | | | 24.1 | | | | | | | | | | | | | | | | | | | | 46 | | | | | | | | | | | | | | | | | | | | | | | 23.2 | | | | | | | | | | | | | | | | | | 0.737 | | | | | | | | | | | |
|  | | | | | | | | | | | | | | | | | | | | | | | | | | | | | | | | | | | | | | | | | | | | | | Frequently | | | | | | | | | | | | | | | | | | | | | 1 | | | | | | | | | | | | | | | | | | | | | | | | | | 0.1 | | | | | | | | | | | | | | | | | | | | 1 | | | | | | | | | | | | | | | | | | | | | | | 0.5 | | | | | | | | | | | | | | | | | |  | | | | | | | | | | | |
|  | | | | | | | | | | | | | | | | | | | | | | | | | | | | | | | | | | | | | | | | | | | | | | Sometimes | | | | | | | | | | | | | | | | | | | | | 29 | | | | | | | | | | | | | | | | | | | | | | | | | | 3.6 | | | | | | | | | | | | | | | | | | | | 8 | | | | | | | | | | | | | | | | | | | | | | | 4.0 | | | | | | | | | | | | | | | | | |  | | | | | | | | | | | |
|  | | | | | | | | | | | | | | | | | | | | | | | | | | | | | | | | | | | | | | | | | | | | | | Rarely | | | | | | | | | | | | | | | | | | | | | 2 | | | | | | | | | | | | | | | | | | | | | | | | | | 0.3 | | | | | | | | | | | | | | | | | | | | 0 | | | | | | | | | | | | | | | | | | | | | | | 0.0 | | | | | | | | | | | | | | | | | |  | | | | | | | | | | | |
|  | | | | | | | | | | | | | | | | | | | | | | | | | | | | | | | | | | | | | | | | | | | | | | Never | | | | | | | | | | | | | | | | | | | | | 576 | | | | | | | | | | | | | | | | | | | | | | | | | | 71.9 | | | | | | | | | | | | | | | | | | | | 143 | | | | | | | | | | | | | | | | | | | | | | | 72.2 | | | | | | | | | | | | | | | | | |  | | | | | | | | | | | |
|  | | | | | | | | | | | | | | | | | | | | | | | | | | | | | | | | | | | | | | | | | | | | | | | | | | | | | | | | | | | | | | | | | | | | | | | | | | | | | | | | | | | | | | | | | | | | | | | | | | | | | | | | | | | | | | | | | | | | | | | | | | | | | | | | | | | | | | | | | | | | | | | | | | | | | | | | | | | | | | | | | | | | | |
|  | | | | | | | | | | | | | | | | | | | | | | | | | | | | | | | | | | | | | | | | | | | | | | | | | | | | | | | | | | | | | | | | | | | | | | | | | | | | | | | | | | | | | | | | | | | | | | | | | | | | | | | | | | | | | | | | | | | | | | | | | | | | | | | | | | | | | | | | | | | | | | | | | | | | | | | | | | | | | | | | | | | | | |
| **Sleeping outside the village due to farming and herding activities – in the dry season** | | | | | | | | | | | | | | | | | | | | | | | | | | | | | | | | | | | | | | | | | | | | | | | | | | | | | | | | | | | | | | | | | | | | | | | | | | | | | | | | | | | | | | | | | | | | | | | | | | | | | | | | | | | | | | | | | | | | | | | | | | | | | | | | | | | | | | | | | | | | | | | | | | | | | | | | | | | | | | | | | | | | | |
| **Sleeping in the field/farm** | | | | | | | | | | | | | | | | | | | | | | | | | | | | | | | | | | | | | | | | | | | | | Yes | | | | | | | | | | | | | | | | | | | | | | 12 | | | | | | | | | | | | | | | | | | | | | | | | | | 1.5 | | | | | | | | | | | | | | | | | | | | 2 | | | | | | | | | | | | | | | | | | | | | | | 1.0 | | | | | | | | | | | | | | | | | | 1.000 | | | | | | | | | | | |
|  | | | | | | | | | | | | | | | | | | | | | | | | | | | | | | | | | | | | | | | | | | | | | Never | | | | | | | | | | | | | | | | | | | | | | 789 | | | | | | | | | | | | | | | | | | | | | | | | | | 98.5 | | | | | | | | | | | | | | | | | | | | 196 | | | | | | | | | | | | | | | | | | | | | | | 99.0 | | | | | | | | | | | | | | | | | |  | | | | | | | | | | | |
| If yes, sleep location | | | | | | | | | | | | | | | | | | | | | | | | | | | | | | | | | | | | | | | Inside a tukul | | | | | | | | | | | | | | | | | | | | | | | | | | | | 2 | | | | | | | | | | | | | | | | | | | | | | | | | | 16.7 | | | | | | | | | | | | | | | | | | | | 1 | | | | | | | | | | | | | | | | | | | | | | | 50.0 | | | | | | | | | | | | | | | | | | 0.396 | | | | | | | | | | | |
| (1 don't know, 2 “variable”) | | | | | | | | | | | | | | | | | | | | | | | | | | | | | | | | | | | | | | | Under a shelter | | | | | | | | | | | | | | | | | | | | | | | | | | | | 0 | | | | | | | | | | | | | | | | | | | | | | | | | |  | | | | | | | | | | | | | | | | | | | | 0 | | | | | | | | | | | | | | | | | | | | | | |  | | | | | | | | | | | | | | | | | |  | | | | | | | | | | | |
|  | | | | | | | | | | | | | | | | | | | | | | | | | | | | | | | | | | | | | | | In an open space | | | | | | | | | | | | | | | | | | | | | | | | | | | | 10 | | | | | | | | | | | | | | | | | | | | | | | | | | 83.3 | | | | | | | | | | | | | | | | | | | | 1 | | | | | | | | | | | | | | | | | | | | | | | 50.0 | | | | | | | | | | | | | | | | | |  | | | | | | | | | | | |
|  | | | | | | | | | | | | | | | | | | | | | | | | | | | | | | | | | | | | | | | Under a tree | | | | | | | | | | | | | | | | | | | | | | | | | | | | 0 | | | | | | | | | | | | | | | | | | | | | | | | | |  | | | | | | | | | | | | | | | | | | | | 0 | | | | | | | | | | | | | | | | | | | | | | |  | | | | | | | | | | | | | | | | | |  | | | | | | | | | | | |
| When sleeping in the field/farm, bed-net use | | | | | | | | | | | | | | | | | | | | | | | | | | | | | | | | | | | | | | | | | | | | | | | | | | | | | | | | | | |  | | | | | | | |  | | | | | | | | | | | | | | | | | | | | | | | | | |  | | | | | | | | | | | | | | | | | | | |  | | | | | | | | | | | | | | | | | | | | | | |  | | | | | | | | | | | | | | | | | | 0.473 | | | | | | | | | | | |
| (1 don't know) | | | | | | | | | | | | | | | | | | | | | Daily/sometimes/frequently | | | | | | | | | | | | | | | | | | | | | | | | | | | | | | | | | | | | | | | | | | | | | | 6 | | | | | | | | | | | | | | | | | | | | | | | | | | 50.0 | | | | | | | | | | | | | | | | | | | | 0 | | | | | | | | | | | | | | | | | | | | | | | 0.0 | | | | | | | | | | | | | | | | | |  | | | | | | | | | | | |
|  | | | | | | | | | | | | | | | | | | | | | Rarely/never | | | | | | | | | | | | | | | | | | | | | | | | | | | | | | | | | | | | | | | | | | | | | | 6 | | | | | | | | | | | | | | | | | | | | | | | | | | 50.0 | | | | | | | | | | | | | | | | | | | | 2 | | | | | | | | | | | | | | | | | | | | | | | 100.0 | | | | | | | | | | | | | | | | | |  | | | | | | | | | | | |
| Nb of nights in the farm over the season | | | | | | | | | | | | | | | | | | | | | | | | | | | | | | | | | | | | | | | | | | Median (IQR) | | | | | | | | | | | | | | 7 | | | | | | | | | | | | | | (5,45) | | | | | | | | | | | | | | | | | | | | 57 | | | | | | | | | | | (15,99) | | | | | | | | | | | | | | | | | | | | | | | | | | | | | | | | | | | | | | | | | | | | | | | | | | | | | 0.228 | | | | | | | | | | | |
| **Herding animals** | | | | | | | | | | | | | | | | | | | | | | | | | | | | | | | | | | | | | | | | | | | | | | Yes | | | | | | | | | | | | | | | | | | | | | 268 | | | | | | | | | | | | | | | | | | | | | | | | | | 33.5 | | | | | | | | | | | | | | | | | | | | 67 | | | | | | | | | | | | | | | | | | | | | | | 33.8 | | | | | | | | | | | | | | | | | | 0.919 | | | | | | | | | | | |
|  | | | | | | | | | | | | | | | | | | | | | | | | | | | | | | | | | | | | | | | | | | | | | | Never | | | | | | | | | | | | | | | | | | | | | 533 | | | | | | | | | | | | | | | | | | | | | | | | | | 66.5 | | | | | | | | | | | | | | | | | | | | 131 | | | | | | | | | | | | | | | | | | | | | | | 66.2 | | | | | | | | | | | | | | | | | |  | | | | | | | | | | | |
| If yes, sleep outside village for herding | | | | | | | | | | | | | | | | | | | | | | | | | | | | | | | | | | | | | | | | | | | | | | | | | | | | Yes | | | | | | | | | | | | | | | 11 | | | | | | | | | | | | | | | | | | | | | | | | | | 4.1 | | | | | | | | | | | | | | | | | | | | 2 | | | | | | | | | | | | | | | | | | | | | | | 3.0 | | | | | | | | | | | | | | | | | | 0.671 | | | | | | | | | | | |
|  | | | | | | | | | | | | | | | | | | | | | | | | | | | | | | | | | | | | | | | | | | | | | | | | | | | | Never | | | | | | | | | | | | | | | 257 | | | | | | | | | | | | | | | | | | | | | | | | | | 95.9 | | | | | | | | | | | | | | | | | | | | 65 | | | | | | | | | | | | | | | | | | | | | | | 97.0 | | | | | | | | | | | | | | | | | |  | | | | | | | | | | | |
| If yes, location | | | | | | | | | | | | | | | | | | | | | | | | | | | | | | | | | | | In a tukul | | | | | | | | | | | | | | | | | | | | | | | | | | | | | | | | 0 | | | | | | | | | | | | | | | | | | | | | | | | | | 0.0 | | | | | | | | | | | | | | | | | | | | 1 | | | | | | | | | | | | | | | | | | | | | | | 50.0 | | | | | | | | | | | | | | | | | | 0.154 | | | | | | | | | | | |
|  | | | | | | | | | | | | | | | | | | | | | | | | | | | | | | | | | | | In an open space | | | | | | | | | | | | | | | | | | | | | | | | | | | | | | | | 11 | | | | | | | | | | | | | | | | | | | | | | | | | | 100.0 | | | | | | | | | | | | | | | | | | | | 1 | | | | | | | | | | | | | | | | | | | | | | | 50.0 | | | | | | | | | | | | | | | | | |  | | | | | | | | | | | |
| If yes, bed-net use | | | | | | | | | | | | | | | | | | | | | | | | | | | | | | | | | | | | | | | | | | | | | | Daily | | | | | | | | | | | | | | | | | | | | | 0 | | | | | | | | | | | | | | | | | | | | | | | | | | 0.0 | | | | | | | | | | | | | | | | | | | | 1 | | | | | | | | | | | | | | | | | | | | | | | 50.0 | | | | | | | | | | | | | | | | | | 0.154 | | | | | | | | | | | |
|  | | | | | | | | | | | | | | | | | | | | | | | | | | | | | | | | | | | | | | | | | | | | | | Never | | | | | | | | | | | | | | | | | | | | | 11 | | | | | | | | | | | | | | | | | | | | | | | | | | 100.0 | | | | | | | | | | | | | | | | | | | | 1 | | | | | | | | | | | | | | | | | | | | | | | 50.0 | | | | | | | | | | | | | | | | | |  | | | | | | | | | | | |
| Nb of nights outside village for herding | | | | | | | | | | | | | | | | | | | | | | | | | | | | | | | | | | | | | | | | | | | | | | | | | | | | | | Median (IQR) | | | | | | | | | | | | | | | | | | | | | | | | | | | 4 | | | | | | (2,30) | | | | | | | | | | | | | | | | | | | 31 | | | | | | | | | | | | | | (2,60) | | | | | | | | | | | | | | | | | | | | | | | | | | | | | | | 0.550 | | | | | | | | | | | | | | |
| **Use of repellents for the participant in the dry season** | | | | | | | | | | | | | | | | | | | | | | | | | | | | | | | | | | | | | | | | | | | | | | | | | | | | | | | | | | | | | | | | | | | | | | | | | | | | | | | | | | | | | | | | | | | | | | | | | | | | | | | | | | | | | | | | | | | | | | | | | | | | | | | | | | | | | | | | | | | | | | | | | | | | | | | | | | | | | | | | | | | | | |
| Chemical insect repellent | | | | | | | | | | | | | | | | | | | | | | | | | | | | | | | | | | | | | | Daily | | | | | | | | | | | | | | | | | | | | | | | | | | | | | 1 | | | | | | | | | | | | | | | | | | | | | | | | | | 0.1 | | | | | | | | | | | | | | | | | | | | 1 | | | | | | | | | | | | | | | | | | | | | | | 0.5 | | | | | | | | | | | | | | | | | | 0.357 | | | | | | | | | | | |
| (2 missing) | | | | | | | | | | | | | | | | | | | | | | | | | | | | | | | | | | | | | | Frequently | | | | | | | | | | | | | | | | | | | | | | | | | | | | | 7 | | | | | | | | | | | | | | | | | | | | | | | | | | 0.9 | | | | | | | | | | | | | | | | | | | | 1 | | | | | | | | | | | | | | | | | | | | | | | 0.5 | | | | | | | | | | | | | | | | | |  | | | | | | | | | | | |
|  | | | | | | | | | | | | | | | | | | | | | | | | | | | | | | | | | | | | | | Sometimes | | | | | | | | | | | | | | | | | | | | | | | | | | | | | 3 | | | | | | | | | | | | | | | | | | | | | | | | | | 0.4 | | | | | | | | | | | | | | | | | | | | 1 | | | | | | | | | | | | | | | | | | | | | | | 0.5 | | | | | | | | | | | | | | | | | |  | | | | | | | | | | | |
|  | | | | | | | | | | | | | | | | | | | | | | | | | | | | | | | | | | | | | | Rarely | | | | | | | | | | | | | | | | | | | | | | | | | | | | | 1 | | | | | | | | | | | | | | | | | | | | | | | | | | 0.1 | | | | | | | | | | | | | | | | | | | | 1 | | | | | | | | | | | | | | | | | | | | | | | 0.5 | | | | | | | | | | | | | | | | | |  | | | | | | | | | | | |
|  | | | | | | | | | | | | | | | | | | | | | | | | | | | | | | | | | | | | | | Never | | | | | | | | | | | | | | | | | | | | | | | | | | | | | 787 | | | | | | | | | | | | | | | | | | | | | | | | | | 98.5 | | | | | | | | | | | | | | | | | | | | 194 | | | | | | | | | | | | | | | | | | | | | | | 99.0 | | | | | | | | | | | | | | | | | |  | | | | | | | | | | | |
| Natural oil or other body products | | | | | | | | | | | | | | | | | | | | | | | | | | | | | | | | | | | | | | | | | | | | | | Daily | | | | | | | | | | | | | | | | | | | | | 212 | | | | | | | | | | | | | | | | | | | | | | | | | | 26.5 | | | | | | | | | | | | | | | | | | | | 59 | | | | | | | | | | | | | | | | | | | | | | | 30.0 | | | | | | | | | | | | | | | | | | 0.098 | | | | | | | | | | | |
| (3 missing) | | | | | | | | | | | | | | | | | | | | | | | | | | | | | | | | | | | | | | | | | | | | | | Frequently | | | | | | | | | | | | | | | | | | | | | 21 | | | | | | | | | | | | | | | | | | | | | | | | | | 2.6 | | | | | | | | | | | | | | | | | | | | 10 | | | | | | | | | | | | | | | | | | | | | | | 5.1 | | | | | | | | | | | | | | | | | |  | | | | | | | | | | | |
|  | | | | | | | | | | | | | | | | | | | | | | | | | | | | | | | | | | | | | | | | | | | | | | Sometimes | | | | | | | | | | | | | | | | | | | | | 187 | | | | | | | | | | | | | | | | | | | | | | | | | | 23.4 | | | | | | | | | | | | | | | | | | | | 47 | | | | | | | | | | | | | | | | | | | | | | | 23.9 | | | | | | | | | | | | | | | | | |  | | | | | | | | | | | |
|  | | | | | | | | | | | | | | | | | | | | | | | | | | | | | | | | | | | | | | | | | | | | | | Rarely | | | | | | | | | | | | | | | | | | | | | 13 | | | | | | | | | | | | | | | | | | | | | | | | | | 1.6 | | | | | | | | | | | | | | | | | | | | 6 | | | | | | | | | | | | | | | | | | | | | | | 3.1 | | | | | | | | | | | | | | | | | |  | | | | | | | | | | | |
|  | | | | | | | | | | | | | | | | | | | | | | | | | | | | | | | | | | | | | | | | | | | | | | Never | | | | | | | | | | | | | | | | | | | | | 366 | | | | | | | | | | | | | | | | | | | | | | | | | | 45.8 | | | | | | | | | | | | | | | | | | | | 75 | | | | | | | | | | | | | | | | | | | | | | | 38.1 | | | | | | | | | | | | | | | | | |  | | | | | | | | | | | |
| Type of oil/product | | | | | | | | | | | | | | | | | | | | | | | | | | | | | | | Sesame oil | | | | | | | | | | | | | | | | | | | | | | | | | | | | | | | | | | | | 145 | | | | | | | | | | | | | | | | | | | | | | | | | | 18.1 | | | | | | | | | | | | | | | | | | | | 42 | | | | | | | | | | | | | | | | | | | | | | | 21.2 | | | | | | | | | | | | | | | | | | 0.315 | | | | | | | | | | | |
|  | | | | | | | | | | | | | | | | | | | | | | | | | | | | | | | Ground nut / beans oil | | | | | | | | | | | | | | | | | | | | | | | | | | | | | | | | | | | | 291 | | | | | | | | | | | | | | | | | | | | | | | | | | 36.3 | | | | | | | | | | | | | | | | | | | | 79 | | | | | | | | | | | | | | | | | | | | | | | 39.9 | | | | | | | | | | | | | | | | | | 0.352 | | | | | | | | | | | |
|  | | | | | | | | | | | | | | | | | | | | | | | | | | | | | | | Gasoline | | | | | | | | | | | | | | | | | | | | | | | | | | | | | | | | | | | | 4 | | | | | | | | | | | | | | | | | | | | | | | | | | 0.5 | | | | | | | | | | | | | | | | | | | | 1 | | | | | | | | | | | | | | | | | | | | | | | 0.5 | | | | | | | | | | | | | | | | | | 1.000 | | | | | | | | | | | |
|  | | | | | | | | | | | | | | | | | | | | | | | | | | | | | | | Other oil | | | | | | | | | | | | | | | | | | | | | | | | | | | | | | | | | | | | 5 | | | | | | | | | | | | | | | | | | | | | | | | | | 0.6 | | | | | | | | | | | | | | | | | | | | 1 | | | | | | | | | | | | | | | | | | | | | | | 0.5 | | | | | | | | | | | | | | | | | | 1.000 | | | | | | | | | | | |
| Thematic section (household level): Characteristics of the household | | | | | | | | | | | | | | | | | | | | | | | | | | | | | | | | | | | | | | | | | | | | | | | | | | | | | | | | | | | | | | | | | | | | | | | | | | | | | | | | | | | | | | | | | | | | | | | | | | | | | | | | | | | | | | | | | | | | | | | | | | | | | | | | | | | | | | | | | | | | | | | | | | | | | | | | | | | | | | | | | | | | | |
| Mean household size (Standard Deviation) | | | | | | | | | | | | | | | | | | | | | | | | | | | | | | | | | | | | | | | | | | | | | | | | | | | | | | | | | | | | | 7.7 | | | | | | | | | | | | | | | | | | | | | | | | (3.3) | | | | | | | | | | | | | | | | | | | | | | | | | 8.4 | | | | | | | | | | | | | | | | | | | | | (4.0) | | | | | | | | | | | | | | | | | | 0.029 | | | | | | | | | | | | | | | | |
| **Characteristics of the head of the household** | | | | | | | | | | | | | | | | | | | | | | | | | | | | | | | | | | | | | | | | | | | | | | | | | | | | | | | | | | | | | | | | | | | | | | | | | | | | | | | | | | | | | | | | | | | | | | | | | | | | | | | | | | | | | | | | | | | | | | | | | | | | | | | | | | | | | | | | | | | | | | | | | | | | | | | | | | | | | | | | | | | | | |
| Female | | | | | | | | | | | | |  | | | | | | | | | | | | | | | | | | | | | | | | | | | | | | | | | | | | | | | | | | | | | | | 38 | | | | | | | | | | | | | | | | | | | | | | | 4.7 | | | | | | | | | | | | | | | | | | | | | | | | | 5 | | | | | | | | | | | | | | | | | | | | | | 2.5 | | | | | | | | | | | | | | | | | | 0.168 | | | | | | | | | | | | | | | | | |
| Education | | | | | | | | | | | | | Illiterate | | | | | | | | | | | | | | | | | | | | | | | | | | | | | | | | | | | | | | | | | | | | | | | 95 | | | | | | | | | | | | | | | | | | | | | | | 12.1 | | | | | | | | | | | | | | | | | | | | | | | | | 18 | | | | | | | | | | | | | | | | | | | | | | 9.1 | | | | | | | | | | | | | | | | | | 0.038 | | | | | | | | | | | | | | | | | |
| (1 missing) | | | | | | | | | | | | | Uncompleted 1ary school | | | | | | | | | | | | | | | | | | | | | | | | | | | | | | | | | | | | | | | | | | | | | | | 153 | | | | | | | | | | | | | | | | | | | | | | | 19.4 | | | | | | | | | | | | | | | | | | | | | | | | | 56 | | | | | | | | | | | | | | | | | | | | | | 28.3 | | | | | | | | | | | | | | | | | |  | | | | | | | | | | | | | | | | | |
| (13 don't know) | | | | | | | | | | | | | Completed 1ary school | | | | | | | | | | | | | | | | | | | | | | | | | | | | | | | | | | | | | | | | | | | | | | | 37 | | | | | | | | | | | | | | | | | | | | | | | 4.7 | | | | | | | | | | | | | | | | | | | | | | | | | 7 | | | | | | | | | | | | | | | | | | | | | | 3.5 | | | | | | | | | | | | | | | | | |  | | | | | | | | | | | | | | | | | |
|  | | | | | | | | | | | | | Uncompleted 2ary school | | | | | | | | | | | | | | | | | | | | | | | | | | | | | | | | | | | | | | | | | | | | | | | 33 | | | | | | | | | | | | | | | | | | | | | | | 4.2 | | | | | | | | | | | | | | | | | | | | | | | | | 14 | | | | | | | | | | | | | | | | | | | | | | 7.1 | | | | | | | | | | | | | | | | | |  | | | | | | | | | | | | | | | | | |
|  | | | | | | | | | | | | | Completed 2ary school | | | | | | | | | | | | | | | | | | | | | | | | | | | | | | | | | | | | | | | | | | | | | | | 26 | | | | | | | | | | | | | | | | | | | | | | | 3.3 | | | | | | | | | | | | | | | | | | | | | | | | | 7 | | | | | | | | | | | | | | | | | | | | | | 3.5 | | | | | | | | | | | | | | | | | |  | | | | | | | | | | | | | | | | | |
|  | | | | | | | | | | | | | Literate through Koranic | | | | | | | | | | | | | | | | | | | | | | | | | | | | | | | | | | | | | | | | | | | | | | | 443 | | | | | | | | | | | | | | | | | | | | | | | 56.3 | | | | | | | | | | | | | | | | | | | | | | | | | 96 | | | | | | | | | | | | | | | | | | | | | | 48.5 | | | | | | | | | | | | | | | | | |  | | | | | | | | | | | | | | | | | |
| Occupation | | | | | | | | | | | | | Agriculture | | | | | | | | | | | | | | | | | | | | | | | | | | | | | | | | | | | | | | | | | | | | | | | 481 | | | | | | | | | | | | | | | | | | | | | | | 60.1 | | | | | | | | | | | | | | | | | | | | | | | | | 118 | | | | | | | | | | | | | | | | | | | | | | 59.6 | | | | | | | | | | | | | | | | | | 0.866 | | | | | | | | | | | | | | | | | |
|  | | | | | | | | | | | | | Skilled labour / salaried work | | | | | | | | | | | | | | | | | | | | | | | | | | | | | | | | | | | | | | | | | | | | | | | 198 | | | | | | | | | | | | | | | | | | | | | | | 24.7 | | | | | | | | | | | | | | | | | | | | | | | | | 49 | | | | | | | | | | | | | | | | | | | | | | 24.8 | | | | | | | | | | | | | | | | | |  | | | | | | | | | | | | | | | | | |
|  | | | | | | | | | | | | | Trade | | | | | | | | | | | | | | | | | | | | | | | | | | | | | | | | | | | | | | | | | | | | | | | 102 | | | | | | | | | | | | | | | | | | | | | | | 12.7 | | | | | | | | | | | | | | | | | | | | | | | | | 28 | | | | | | | | | | | | | | | | | | | | | | 14.1 | | | | | | | | | | | | | | | | | |  | | | | | | | | | | | | | | | | | |
|  | | | | | | | | | | | | | Unemployed | | | | | | | | | | | | | | | | | | | | | | | | | | | | | | | | | | | | | | | | | | | | | | | 20 | | | | | | | | | | | | | | | | | | | | | | | 2.5 | | | | | | | | | | | | | | | | | | | | | | | | | 3 | | | | | | | | | | | | | | | | | | | | | | 1.5 | | | | | | | | | | | | | | | | | |  | | | | | | | | | | | | | | | | | |
|  | | | | | | | | | | | | | | | | | | | | | | | | | | | | | | | | | | | | | | | | | | | | | | | | | | | | | | | | | | | | | | | | | | | | | | | | | | | | | | | | | | | | | | | | | | | | | | | | | | | | | | | | | | | | | | | | | | | | | | | | | | | | | | | | | | | | | | | | | | | | | | | | | | | | | | | | | | | | | | | | | | | | | |
|  | | | | | | | | | | | | | | | | | | | | | | | | | | | | | | | | | | | | | | | | | | | | | | | | | | | | | | | | | | | | | | | | | | | | | | | | | | | | | | | | | | | | | | | | | | | | | | | | | | | | | | | | | | | | | | | | | | | | | | | | | | | | | | | | | | | | | | | | | | | | | | | | | | | | | | | | | | | | | | | | | | | | | |
| **Household ownership and socioeconomic index** | | | | | | | | | | | | | | | | | | | | | | | | | | | | | | | | | | | | | | | | | | | | | | | | | | | | | | | | | | | | | | | | | | | | | | | | | | | | | | | | | | | | | | | | | | | | | | | | | | | | | | | | | | | | | | | | | | | | | | | | | | | | | | | | | | | | | | | | | | | | | | | | | | | | | | | | | | | | | | | | | | | | | |
| **Ownership** | | | | | | | | | | | | | | | | | | | | | | | | | | | | | | | | | | | | | | | | | | | | | | | | | | | | | | | | | | | |  | | | | | | | | | | | | | | | | | | | | | | | | | | | | | | |  | | | | | | | | | | | | | | | | |  | | | | | | | | | | | | | | | | | | | | | | | | | |  | | | | | | | | | | | | | | | | | |  | | | | | | | | | | | | | |
| Mobile phone | | | | | | | | | | | | | | | | | | | | | | | | | | | | | | | | | | | | | | | | | | | | | | | | | | | | | | | | |  | | | 721 | | | | | | | | | | | | | | | | | | | | | | | | | | | | | | | 90.0 | | | | | | | | | | | | | | | | | 187 | | | | | | | | | | | | | | | | | | | | | | | | | | 94.4 | | | | | | | | | | | | | | | | | | 0.052 | | | | | | | | | | | | | |
| Radio | | | | | | | | | | | | | | | | | | | | | | | | | | | | | | | | | | | | | | | | | | | | | | | | | | | | | | | | |  | | | 471 | | | | | | | | | | | | | | | | | | | | | | | | | | | | | | | 58.8 | | | | | | | | | | | | | | | | | 120 | | | | | | | | | | | | | | | | | | | | | | | | | | 60.6 | | | | | | | | | | | | | | | | | | 0.644 | | | | | | | | | | | | | |
| Bicycle | | | | | | | | | | | | | | | | | | | | | | | | | | | | | | | | | | | | | | | | | | | | | | | | | | | | | | | | |  | | | 215 | | | | | | | | | | | | | | | | | | | | | | | | | | | | | | | 26.8 | | | | | | | | | | | | | | | | | 56 | | | | | | | | | | | | | | | | | | | | | | | | | | 28.3 | | | | | | | | | | | | | | | | | | 0.683 | | | | | | | | | | | | | |
| Donkey cart | | | | | | | | | | | | | | | | | | | | | | | | | | | | | | | | | | | | | | | | | | | | | | | | | | | | | | | | |  | | | 193 | | | | | | | | | | | | | | | | | | | | | | | | | | | | | | | 24.1 | | | | | | | | | | | | | | | | | 72 | | | | | | | | | | | | | | | | | | | | | | | | | | 36.4 | | | | | | | | | | | | | | | | | | <0.001 | | | | | | | | | | | | | |
| Generator | | | | | | | | | | | | | | | | | | | | | | | | | | | | | | | | | | | | | | | | | | | | | | | | | | | | | | | | |  | | | 79 | | | | | | | | | | | | | | | | | | | | | | | | | | | | | | | 9.9 | | | | | | | | | | | | | | | | | 19 | | | | | | | | | | | | | | | | | | | | | | | | | | 9.6 | | | | | | | | | | | | | | | | | | 0.910 | | | | | | | | | | | | | |
| Car, truck or tractor | | | | | | | | | | | | | | | | | | | | | | | | | | | | | | | | | | | | | | | | | | | | | | | | | | | | | | | | |  | | | 74 | | | | | | | | | | | | | | | | | | | | | | | | | | | | | | | 9.2 | | | | | | | | | | | | | | | | | 13 | | | | | | | | | | | | | | | | | | | | | | | | | | 6.6 | | | | | | | | | | | | | | | | | | 0.232 | | | | | | | | | | | | | |
| Motorcycle | | | | | | | | | | | | | | | | | | | | | | | | | | | | | | | | | | | | | | | | | | | | | | | | | | | | | | | | |  | | | 68 | | | | | | | | | | | | | | | | | | | | | | | | | | | | | | | 8.5 | | | | | | | | | | | | | | | | | 12 | | | | | | | | | | | | | | | | | | | | | | | | | | 6.1 | | | | | | | | | | | | | | | | | | 0.260 | | | | | | | | | | | | | |
| TV | | | | | | | | | | | | | | | | | | | | | | | | | | | | | | | | | | | | | | | | | | | | | | | | | | | | | | | | |  | | | 52 | | | | | | | | | | | | | | | | | | | | | | | | | | | | | | | 6.5 | | | | | | | | | | | | | | | | | 18 | | | | | | | | | | | | | | | | | | | | | | | | | | 9.1 | | | | | | | | | | | | | | | | | | 0.200 | | | | | | | | | | | | | |
| House (“tukul”) in this house yard | | | | | | | | | | | | | | | | | | | | | | | | | | | | | | | | | | | | | | | | | | | | | | | | | | | | | | | | | | | | | | | 795 | | | | | | | | | | | | | | | | | | | | | | | | | | | | | 99.3 | | | | | | | | | | | | | | | | | 197 | | | | | | | | | | | | | | | | | | | | | | | | | | 99.5 | | | | | | | | | | | | | | | | | | 1.00 | | | | | | | | | | | | |
| House (“tukul”) outside this house yard | | | | | | | | | | | | | | | | | | | | | | | | | | | | | | | | | | | | | | | | | | | | | | | | | | | | | | | | | | | | | | | 132 | | | | | | | | | | | | | | | | | | | | | | | | | | | | | 16.5 | | | | | | | | | | | | | | | | | 26 | | | | | | | | | | | | | | | | | | | | | | | | | | 13.1 | | | | | | | | | | | | | | | | | | 0.248 | | | | | | | | | | | | |
| Fields | | | | | | | | | | | | | | | | | | | | | | | | | | | |  | | | | | | | | | | | | | | | | | | | | | | | | | | | | | | | | | | | 550 | | | | | | | | | | | | | | | | | | | | | | | | | | | | | 68.7 | | | | | | | | | | | | | | | | | 136 | | | | | | | | | | | | | | | | | | | | | | | | | | 68.7 | | | | | | | | | | | | | | | | | | 0.995 | | | | | | | | | | | | |
| Animals for trade | | | | | | | | | | | | | | | | | | | | | | | | | | | |  | | | | | | | | | | | | | | | | | | | | | | | | | | | | | | | | | | | 75 | | | | | | | | | | | | | | | | | | | | | | | | | | | | | 9.4 | | | | | | | | | | | | | | | | | 14 | | | | | | | | | | | | | | | | | | | | | | | | | | 7.1 | | | | | | | | | | | | | | | | | | 0.311 | | | | | | | | | | | | |
| **Cereal stock** | | | | | | | | | | | | | | | | | | | In the house yard | | | | | | | | | | | | | | | | | | | | | | | | | | | | | | | | | | | | | | | | | | | | 272 | | | | | | | | | | | | | | | | | | | | | | | | | | | | | 34.0 | | | | | | | | | | | | | | | | | 63 | | | | | | | | | | | | | | | | | | | | | | | | | | 31.8 | | | | | | | | | | | | | | | | | | 0.568 | | | | | | | | | | | | |
|  | | | | | | | | | | | | | | | | | | | In the house (“tukul”) | | | | | | | | | | | | | | | | | | | | | | | | | | | | | | | | | | | | | | | | | | | | 222 | | | | | | | | | | | | | | | | | | | | | | | | | | | | | 27.7 | | | | | | | | | | | | | | | | | 59 | | | | | | | | | | | | | | | | | | | | | | | | | | 29.8 | | | | | | | | | | | | | | | | | | 0.559 | | | | | | | | | | | | |
|  | | | | | | | | | | | | | | | | | | | Outside the house yard | | | | | | | | | | | | | | | | | | | | | | | | | | | | | | | | | | | | | | | | | | | | 89 | | | | | | | | | | | | | | | | | | | | | | | | | | | | | 11.1 | | | | | | | | | | | | | | | | | 19 | | | | | | | | | | | | | | | | | | | | | | | | | | 9.6 | | | | | | | | | | | | | | | | | | 0.539 | | | | | | | | | | | | |
| Quality of the main house (“tukul”) | | | | | | | | | | | | | | | | | | | | | | | | | | | | | | | | | | | | | | | | | | | | Very good | | | | | | | | | | | | | | | | | | | 53 | | | | | | | | | | | | | | | | | | | | | | | | | | | | | 6.6 | | | | | | | | | | | | | | | | | 10 | | | | | | | | | | | | | | | | | | | | | | | | | | 5.0 | | | | | | | | | | | | | | | | | | 0.564 | | | | | | | | | | | | |
| (1 Missing) | | | | | | | | | | | | | | | | | | | | | | | | | | | | | | | | | | | | | | | | | | | | Good | | | | | | | | | | | | | | | | | | | 510 | | | | | | | | | | | | | | | | | | | | | | | | | | | | | 63.7 | | | | | | | | | | | | | | | | | 120 | | | | | | | | | | | | | | | | | | | | | | | | | | 60.6 | | | | | | | | | | | | | | | | | |  | | | | | | | | | | | | |
|  | | | | | | | | | | | | | | | | | | | | | | | | | | | | | | | | | | | | | | | | | | | | Poor | | | | | | | | | | | | | | | | | | | 208 | | | | | | | | | | | | | | | | | | | | | | | | | | | | | 26.0 | | | | | | | | | | | | | | | | | 60 | | | | | | | | | | | | | | | | | | | | | | | | | | 30.3 | | | | | | | | | | | | | | | | | |  | | | | | | | | | | | | |
|  | | | | | | | | | | | | | | | | | | | | | | | | | | | | | | | | | | | | | | | | | | | | Very poor | | | | | | | | | | | | | | | | | | | 29 | | | | | | | | | | | | | | | | | | | | | | | | | | | | | 3.6 | | | | | | | | | | | | | | | | | 8 | | | | | | | | | | | | | | | | | | | | | | | | | | 4.0 | | | | | | | | | | | | | | | | | |  | | | | | | | | | | | | |
| Socioeconomic index | | | | | | | | | | | | | | | | | | | | | | | | | | | Lowest | | | | | | | | | | | | | | | | | | | | | | | | | | | | | | | | | | | | 204 | | | | | | | | | | | | | | | | | | | | | | | | | | | | | 25.47 | | | | | | | | | | | | | | | | | | | | 46 | | | | | | | | | | | | | | | | | | | | | | | 23.23 | | | | | | | | | | | | | | | | | | 0.475 | | | | | | | | | | | | |
|  | | | | | | | | | | | | | | | | | | | | | | | | | | | 2nd lowest | | | | | | | | | | | | | | | | | | | | | | | | | | | | | | | | | | | | 255 | | | | | | | | | | | | | | | | | | | | | | | | | | | | | 31.84 | | | | | | | | | | | | | | | | | | | | 54 | | | | | | | | | | | | | | | | | | | | | | | 27.27 | | | | | | | | | | | | | | | | | |  | | | | | | | | | | | | |
|  | | | | | | | | | | | | | | | | | | | | | | | | | | | Middle | | | | | | | | | | | | | | | | | | | | | | | | | | | | | | | | | | | | 34 | | | | | | | | | | | | | | | | | | | | | | | | | | | | | 4.24 | | | | | | | | | | | | | | | | | | | | 8 | | | | | | | | | | | | | | | | | | | | | | | 4.04 | | | | | | | | | | | | | | | | | |  | | | | | | | | | | | | |
|  | | | | | | | | | | | | | | | | | | | | | | | | | | | 2nd highest | | | | | | | | | | | | | | | | | | | | | | | | | | | | | | | | | | | | 185 | | | | | | | | | | | | | | | | | | | | | | | | | | | | | 23.10 | | | | | | | | | | | | | | | | | | | | 52 | | | | | | | | | | | | | | | | | | | | | | | 26.26 | | | | | | | | | | | | | | | | | |  | | | | | | | | | | | | |
|  | | | | | | | | | | | | | | | | | | | | | | | | | | | Highest | | | | | | | | | | | | | | | | | | | | | | | | | | | | | | | | | | | | 123 | | | | | | | | | | | | | | | | | | | | | | | | | | | | | 15.36 | | | | | | | | | | | | | | | | | | | | 38 | | | | | | | | | | | | | | | | | | | | | | | 19.19 | | | | | | | | | | | | | | | | | |  | | | | | | | | | | | | |
| **Source of drinking water and toilet location in the dry or the rainy season** | | | | | | | | | | | | | | | | | | | | | | | | | | | | | | | | | | | | | | | | | | | | | | | | | | | | | | | | | | | | | | | | | | | | | | | | | | | | | | | | | | | | | | | | | | | | | | | | | | | | | | | | | | | | | | | | | | | | | | | | | | | | | | | | | | | | | | | | | | | | | | | | | | | | | | | | | | | | | | | | | | | | | |
| Source of drinking water in the dry season | | | | | | | | | | | | | | | | | | | | | | | | | | | | | | | | | | | | | | | | | | | | | | | | | | | | | | | | | | | | | | |  | | | | | | | | | | | | | | | | | | | | | | | | | | | | |  | | | | | | | | | | | | | | | | | | | |  | | | | | | | | | | | | | | | | | | | | | | |  | | | | | | | | | | | | | | | | | | <0.001 | | | | | | | | | | | | |
|  | Village water tank | | | | | | | | | | | | | | | | | | | | | | | | | | | | | | | | | | | | | | | | | | | | | | | | | | | | | | | | | | | | | | 162 | | | | | | | | | | | | | | | | | | | | | | | | | | | | | 20.2 | | | | | | | | | | | | | | | | | | | | 73 | | | | | | | | | | | | | | | | | | | | | | | 36.9 | | | | | | | | | | | | | | | | | |  | | | | | | | | | | | | |
|  | River or surface water | | | | | | | | | | | | | | | | | | | | | | | | | | | | | | | | | | | | | | | | | | | | | | | | | | | | | | | | | | | | | | 342 | | | | | | | | | | | | | | | | | | | | | | | | | | | | | 42.7 | | | | | | | | | | | | | | | | | | | | 41 | | | | | | | | | | | | | | | | | | | | | | | 20.7 | | | | | | | | | | | | | | | | | |  | | | | | | | | | | | | |
|  | Pump, well, water sellers | | | | | | | | | | | | | | | | | | | | | | | | | | | | | | | | | | | | | | | | | | | | | | | | | | | | | | | | | | | | | | 297 | | | | | | | | | | | | | | | | | | | | | | | | | | | | | 37.1 | | | | | | | | | | | | | | | | | | | | 84 | | | | | | | | | | | | | | | | | | | | | | | 42.4 | | | | | | | | | | | | | | | | | |  | | | | | | | | | | | | |
|  | | | ***Details*** | | | | | | | | | | | | | | | | | | | | | | | | | | | | | | | | | | | | | | | | | | | | | | | | | | | | | | | | | | | |  | | | | | | | | | | | | | | | | | | | | | | | | | | | | |  | | | | | | | | | | | | | | | | | | | |  | | | | | | | | | | | | | | | | | | | | | | |  | | | | | | | | | | | | | | | | | |  | | | | | | | | | | | | |
|  | | | *Village water tank* | | | | | | | | | | | | | | | | | | | | | | | | | | | | | | | | | | | | | | | | | | | | | | | | | | | | | | | | | | | | *162* | | | | | | | | | | | | | | | | | | | | | | | | | | | | | *20.2* | | | | | | | | | | | | | | | | | | | | *73* | | | | | | | | | | | | | | | | | | | | | | | *36.9* | | | | | | | | | | | | | | | | | |  | | | | | | | | | | | | |
|  | | | *Stagnant surface water* | | | | | | | | | | | | | | | | | | | | | | | | | | | | | | | | | | | | | | | | | | | | | | | | | | | | | | | | | | | | *330* | | | | | | | | | | | | | | | | | | | | | | | | | | | | | *41.2* | | | | | | | | | | | | | | | | | | | | *39* | | | | | | | | | | | | | | | | | | | | | | | *19.7* | | | | | | | | | | | | | | | | | |  | | | | | | | | | | | | |
|  | | | *River or stream* | | | | | | | | | | | | | | | | | | | | | | | | | | | | | | | | | | | | | | | | | | | | | | | | | | | | | | | | | | | | *11* | | | | | | | | | | | | | | | | | | | | | | | | | | | | | *1.4* | | | | | | | | | | | | | | | | | | | | *2* | | | | | | | | | | | | | | | | | | | | | | | *1.0* | | | | | | | | | | | | | | | | | |  | | | | | | | | | | | | |
|  | | | *Surface water and hand pump* | | | | | | | | | | | | | | | | | | | | | | | | | | | | | | | | | | | | | | | | | | | | | | | | | | | | | | | | | | | | *1* | | | | | | | | | | | | | | | | | | | | | | | | | | | | | *0.1* | | | | | | | | | | | | | | | | | | | | *0* | | | | | | | | | | | | | | | | | | | | | | | *0* | | | | | | | | | | | | | | | | | |  | | | | | | | | | | | | |
|  | | | *Hand water pump* | | | | | | | | | | | | | | | | | | | | | | | | | | | | | | | | | | | | | | | | | | | | | | | | | | | | | | | | | | | | *65* | | | | | | | | | | | | | | | | | | | | | | | | | | | | | *8.1* | | | | | | | | | | | | | | | | | | | | *7* | | | | | | | | | | | | | | | | | | | | | | | *3.5* | | | | | | | | | | | | | | | | | |  | | | | | | | | | | | | |
|  | | | *Well* | | | | | | | | | | | | | | | | | | | | | | | | | | | | | | | | | | | | | | | | | | | | | | | | | | | | | | | | | | | | *199* | | | | | | | | | | | | | | | | | | | | | | | | | | | | | *24.8* | | | | | | | | | | | | | | | | | | | | *61* | | | | | | | | | | | | | | | | | | | | | | | *30.8* | | | | | | | | | | | | | | | | | |  | | | | | | | | | | | | |
|  | | | *Water sellers* | | | | | | | | | | | | | | | | | | | | | | | | | | | | | | | | | | | | | | | | | | | | | | | | | | | | | | | | | | | | *33* | | | | | | | | | | | | | | | | | | | | | | | | | | | | | *4.1* | | | | | | | | | | | | | | | | | | | | *15* | | | | | | | | | | | | | | | | | | | | | | | *7.6* | | | | | | | | | | | | | | | | | |  | | | | | | | | | | | | |
|  | | | *Mineral water* | | | | | | | | | | | | | | | | | | | | | | | | | | | | | | | | | | | | | | | | | | | | | | | | | | | | | | | | | | | | *0* | | | | | | | | | | | | | | | | | | | | | | | | | | | | | *0* | | | | | | | | | | | | | | | | | | | | *1* | | | | | | | | | | | | | | | | | | | | | | | *0.5* | | | | | | | | | | | | | | | | | |  | | | | | | | | | | | | |
| Source of drinking water in the rainy season | | | | | | | | | | | | | | | | | | | | | | | | | | | | | | | | | | | | | | | | | | | | | | | | | | | | | | | | | | | | | | | | | | | | | | | | | | | | | | | | | | | | | | | | | | | | | | | | | | | | | | | | | | | | | | | | | | | | | | | | | | | | | | | | | | | | | | | | | | | | | | | | | | | | | | | | | <0.001 | | | | | | | | | | | | |
|  | Village water tank | | | | | | | | | | | | | | | | | | | | | | | | | | | | | | | | | | | | | | | | | | | | | | | | | | | | | | | | | | | | | | 112 | | | | | | | | | | | | | | | | | | | | | | | | | | | | | 14.0 | | | | | | | | | | | | | | | | | | | | 52 | | | | | | | | | | | | | | | | | | | | | | | 26.3 | | | | | | | | | | | | | | | | | |  | | | | | | | | | | | | |
|  | River, surface water, rain water | | | | | | | | | | | | | | | | | | | | | | | | | | | | | | | | | | | | | | | | | | | | | | | | | | | | | | | | | | | | | | 373 | | | | | | | | | | | | | | | | | | | | | | | | | | | | | 46.6 | | | | | | | | | | | | | | | | | | | | 48 | | | | | | | | | | | | | | | | | | | | | | | 24.2 | | | | | | | | | | | | | | | | | |  | | | | | | | | | | | | |
|  | Pump, well, water sellers | | | | | | | | | | | | | | | | | | | | | | | | | | | | | | | | | | | | | | | | | | | | | | | | | | | | | | | | | | | | | | 316 | | | | | | | | | | | | | | | | | | | | | | | | | | | | | 39.4 | | | | | | | | | | | | | | | | | | | | 98 | | | | | | | | | | | | | | | | | | | | | | | 49.5 | | | | | | | | | | | | | | | | | |  | | | | | | | | | | | | |
|  | | | ***Details*** | | | | | | | | | | | | | | | | | | | | | | | | | | | | | | | | | | | | | | | | | | | | | | | | | | | | | | | | | | | | | | | | | | | | | | | | | | | | | | | | | | | | | | | | | | | | | | | | | | | | | | | | | | | | | | | | | | | | | | | | | | | | | | | | | | | | | | | | | | | | | | | | | | | | | |  | | | | | | | | | | | | |
|  | | | *Village water tank* | | | | | | | | | | | | | | | | | | | | | | | | | | | | | | | | | | | | | | | | | | | | | | | | | | | | | | | | | | | | *112* | | | | | | | | | | | | | | | | | | | | | | | | | | | | | *14.0* | | | | | | | | | | | | | | | | | | | | *52* | | | | | | | | | | | | | | | | | | | | | | | *26.3* | | | | | | | | | | | | | | | | | |  | | | | | | | | | | | | |
|  | | | *River or stream* | | | | | | | | | | | | | | | | | | | | | | | | | | | | | | | | | | | | | | | | | | | | | | | | | | | | | | | | | | | | *316* | | | | | | | | | | | | | | | | | | | | | | | | | | | | | *39.5* | | | | | | | | | | | | | | | | | | | | *39* | | | | | | | | | | | | | | | | | | | | | | | *19.7* | | | | | | | | | | | | | | | | | |  | | | | | | | | | | | | |
|  | | | *Stagnant surface water* | | | | | | | | | | | | | | | | | | | | | | | | | | | | | | | | | | | | | | | | | | | | | | | | | | | | | | | | | | | | *47* | | | | | | | | | | | | | | | | | | | | | | | | | | | | | *5.9* | | | | | | | | | | | | | | | | | | | | *7* | | | | | | | | | | | | | | | | | | | | | | | *3.5* | | | | | | | | | | | | | | | | | |  | | | | | | | | | | | | |
|  | | | *Collected rain water* | | | | | | | | | | | | | | | | | | | | | | | | | | | | | | | | | | | | | | | | | | | | | | | | | | | | | | | | | | | | *10* | | | | | | | | | | | | | | | | | | | | | | | | | | | | | *1.3* | | | | | | | | | | | | | | | | | | | | *2* | | | | | | | | | | | | | | | | | | | | | | | *1.0* | | | | | | | | | | | | | | | | | |  | | | | | | | | | | | | |
|  | | | *Hand water pump* | | | | | | | | | | | | | | | | | | | | | | | | | | | | | | | | | | | | | | | | | | | | | | | | | | | | | | | | | | | | *63* | | | | | | | | | | | | | | | | | | | | | | | | | | | | | *7.9* | | | | | | | | | | | | | | | | | | | | *6* | | | | | | | | | | | | | | | | | | | | | | | *3.0* | | | | | | | | | | | | | | | | | |  | | | | | | | | | | | | |
|  | | | *Well* | | | | | | | | | | | | | | | | | | | | | | | | | | | | | | | | | | | | | | | | | | | | | | | | | | | | | | | | | | | | *226* | | | | | | | | | | | | | | | | | | | | | | | | | | | | | *28.2* | | | | | | | | | | | | | | | | | | | | *76* | | | | | | | | | | | | | | | | | | | | | | | *38.4* | | | | | | | | | | | | | | | | | |  | | | | | | | | | | | | |
|  | | | *Water sellers* | | | | | | | | | | | | | | | | | | | | | | | | | | | | | | | | | | | | | | | | | | | | | | | | | | | | | | | | | | | | *27* | | | | | | | | | | | | | | | | | | | | | | | | | | | | | *3.4* | | | | | | | | | | | | | | | | | | | | *15* | | | | | | | | | | | | | | | | | | | | | | | *7.6* | | | | | | | | | | | | | | | | | |  | | | | | | | | | | | | |
|  | | | | | | | | | | | | | | | | | | | | | | | | | | | | | | | | | | | | | | | | | | | | | | | | | | | | | | | | | | | | | | |  | | | | | | | | | | | | | | | | | | | | | | | | | | | | |  | | | | | | | | | | | | | | | | | | | |  | | | | | | | | | | | | | | | | | | | | | | |  | | | | | | | | | | | | | | | | | |  | | | | | | | | | | | | |
| Toilet (at night) in the dry season (3 missing) | | | | | | | | | | | | | | | | | | | | | | | | | | | | | | | | | | | | | | | | | | | | | | | | | | | | | | | | | | | | | | |  | | | | | | | | | | | | | | | | | | | | | | | | | | | | |  | | | | | | | | | | | | | | | | | | | |  | | | | | | | | | | | | | | | | | | | | | | |  | | | | | | | | | | | | | | | | | | 0.144 | | | | | | | | | | | | |
|  | Outside the house yard, no latrines | | | | | | | | | | | | | | | | | | | | | | | | | | | | | | | | | | | | | | | | | | | | | | | | | | | | | | | | | | | | | | 515 | | | | | | | | | | | | | | | | | | | | | | | | | | | | | 64.5 | | | | | | | | | | | | | | | | | | | | 134 | | | | | | | | | | | | | | | | | | | | | | | 67.7 | | | | | | | | | | | | | | | | | |  | | | | | | | | | | | | |
|  | Latrine in house yard | | | | | | | | | | | | | | | | | | | | | | | | | | | | | | | | | | | | | | | | | | | | | | | | | | | | | | | | | | | | | | 174 | | | | | | | | | | | | | | | | | | | | | | | | | | | | | 21.8 | | | | | | | | | | | | | | | | | | | | 34 | | | | | | | | | | | | | | | | | | | | | | | 17.2 | | | | | | | | | | | | | | | | | |  | | | | | | | | | | | | |
|  | In the house yard, no latrines | | | | | | | | | | | | | | | | | | | | | | | | | | | | | | | | | | | | | | | | | | | | | | | | | | | | | | | | | | | | | | 68 | | | | | | | | | | | | | | | | | | | | | | | | | | | | | 8.5 | | | | | | | | | | | | | | | | | | | | 25 | | | | | | | | | | | | | | | | | | | | | | | 12.6 | | | | | | | | | | | | | | | | | |  | | | | | | | | | | | | |
|  | Latrines outside house yard | | | | | | | | | | | | | | | | | | | | | | | | | | | | | | | | | | | | | | | | | | | | | | | | | | | | | | | | | | | | | | 8 | | | | | | | | | | | | | | | | | | | | | | | | | | | | | 1.0 | | | | | | | | | | | | | | | | | | | | 1 | | | | | | | | | | | | | | | | | | | | | | | 0.5 | | | | | | | | | | | | | | | | | |  | | | | | | | | | | | | |
|  | Not relevant, young child | | | | | | | | | | | | | | | | | | | | | | | | | | | | | | | | | | | | | | | | | | | | | | | | | | | | | | | | | | | | | | 33 | | | | | | | | | | | | | | | | | | | | | | | | | | | | | 4.1 | | | | | | | | | | | | | | | | | | | | 4 | | | | | | | | | | | | | | | | | | | | | | | 2.0 | | | | | | | | | | | | | | | | | |  | | | | | | | | | | | | |
| Toilet (at night) in the rainy season (3 missing) | | | | | | | | | | | | | | | | | | | | | | | | | | | | | | | | | | | | | | | | | | | | | | | | | | | | | | | | | | | | | | |  | | | | | | | | | | | | | | | | | | | | | | | | | | | | |  | | | | | | | | | | | | | | | | | | | |  | | | | | | | | | | | | | | | | | | | | | | |  | | | | | | | | | | | | | | | | | | 0.155 | | | | | | | | | | | | |
|  | Outside the house yard, no latrines | | | | | | | | | | | | | | | | | | | | | | | | | | | | | | | | | | | | | | | | | | | | | | | | | | | | | | | | | | | | | | 514 | | | | | | | | | | | | | | | | | | | | | | | | | | | | | 64.4 | | | | | | | | | | | | | | | | | | | | 134 | | | | | | | | | | | | | | | | | | | | | | | 67.7 | | | | | | | | | | | | | | | | | |  | | | | | | | | | | | | |
|  | Latrine in house yard | | | | | | | | | | | | | | | | | | | | | | | | | | | | | | | | | | | | | | | | | | | | | | | | | | | | | | | | | | | | | | 174 | | | | | | | | | | | | | | | | | | | | | | | | | | | | | 21.8 | | | | | | | | | | | | | | | | | | | | 34 | | | | | | | | | | | | | | | | | | | | | | | 17.2 | | | | | | | | | | | | | | | | | |  | | | | | | | | | | | | |
|  | In the house yard, no latrines | | | | | | | | | | | | | | | | | | | | | | | | | | | | | | | | | | | | | | | | | | | | | | | | | | | | | | | | | | | | | | 69 | | | | | | | | | | | | | | | | | | | | | | | | | | | | | 8.6 | | | | | | | | | | | | | | | | | | | | 25 | | | | | | | | | | | | | | | | | | | | | | | 12.6 | | | | | | | | | | | | | | | | | |  | | | | | | | | | | | | |
|  | Latrines outside house yard | | | | | | | | | | | | | | | | | | | | | | | | | | | | | | | | | | | | | | | | | | | | | | | | | | | | | | | | | | | | | | 8 | | | | | | | | | | | | | | | | | | | | | | | | | | | | | 1.0 | | | | | | | | | | | | | | | | | | | | 1 | | | | | | | | | | | | | | | | | | | | | | | 0.5 | | | | | | | | | | | | | | | | | |  | | | | | | | | | | | | |
|  | Not relevant, young child | | | | | | | | | | | | | | | | | | | | | | | | | | | | | | | | | | | | | | | | | | | | | | | | | | | | | | | | | | | | | | 33 | | | | | | | | | | | | | | | | | | | | | | | | | | | | | 4.1 | | | | | | | | | | | | | | | | | | | | 4 | | | | | | | | | | | | | | | | | | | | | | | 2.0 | | | | | | | | | | | | | | | | | |  | | | | | | | | | | | | |
| Thematic section (household level): House/sleeping room characteristics | | | | | | | | | | | | | | | | | | | | | | | | | | | | | | | | | | | | | | | | | | | | | | | | | | | | | | | | | | | | | | | | | | | | | | | | | | | | | | | | | | | | | | | | | | | | | | | | | | | | | | | | | | | | | | | | | | | | | | | | | | | | | | | | | | | | | | | | | | | | | | | | | | | | | | | | | | | | | | | | | | | | | |
| **Median (IQR) of people sleeping in room** (individuals sleeping in open space excluded) Rainy season (n=993) | | | | | | | | | | | | | | | | | | | | | | | | | | | | | | | | | | | | | | | | | | | | | | | | | | | | | | | | | | | | | | | | | | | | | | | | | | | | | | | | 4 | | | | (3,6) | | | | | | | | | | | | | | | | | | | | | | | | | | | | | | | | | 5 | | | | | | | | | | | (3,6) | | | | | | | | | | | | | | | | | | | | | | | | | | | <0.001 | | | | | | | | | | |
| Dry season (n=506) | | | | | | | | | | | | | | | | | | | | | | | | | | | | | | | | | | | | | | | | | | | | | | | | | | | | | | | | | | | | | | | | | | | | | | | | | | | | | | | | 4 | | | | (3,5) | | | | | | | | | | | | | | | | | | | | | | | | | | | | | | | | | 5 | | | | | | | | | | | (4,6) | | | | | | | | | | | | | | | | | | | | | | | | | | | <0.001 | | | | | | | | | | |
| **Walls** | | | | Cane plastered with mud (1 missing) | | | | | | | | | | | | | | | | | | | | | | | | | | | | | | | | | | | | | | | | | | | | | | | | | | | | | | | | | | | | | | | | | | | 337 | | | | | | | | | | | | | | | | | | 42.1 | | | | | | | | | | | | | | | | | | | 111 | | | | | | | | | | | | | | | | | | | | | | | | | 56.1 | | | | | | | | | | | | | | | | | | | <0.001 | | | | | | | | | | | | | |
|  | | | | Strong mud only | | | | | | | | | | | | | | | | | | | | | | | | | | | | | | | | | | | | | | | | | | | | | | | | | | | | | | | | | | | | | | | | | | | 212 | | | | | | | | | | | | | | | | | | 26.5 | | | | | | | | | | | | | | | | | | | 32 | | | | | | | | | | | | | | | | | | | | | | | | | 16.2 | | | | | | | | | | | | | | | | | | |  | | | | | | | | | | | | | |
|  | | | | Grass/cane/wood mostly unplastered | | | | | | | | | | | | | | | | | | | | | | | | | | | | | | | | | | | | | | | | | | | | | | | | | | | | | | | | | | | | | | | | | | | 201 | | | | | | | | | | | | | | | | | | 25.1 | | | | | | | | | | | | | | | | | | | 49 | | | | | | | | | | | | | | | | | | | | | | | | | 24.8 | | | | | | | | | | | | | | | | | | |  | | | | | | | | | | | | | |
|  | | | | Strong mud with grass or plastic sheeting | | | | | | | | | | | | | | | | | | | | | | | | | | | | | | | | | | | | | | | | | | | | | | | | | | | | | | | | | | | | | | | | | | | 50 | | | | | | | | | | | | | | | | | | 6.3 | | | | | | | | | | | | | | | | | | | 5 | | | | | | | | | | | | | | | | | | | | | | | | | 2.5 | | | | | | | | | | | | | | | | | | |  | | | | | | | | | | | | | |
|  | | | | Concrete-brick | | | | | | | | | | | | | | | | | | | | | | | | | | | | | | | | | | | | | | | | | | | | | | | | | | | | | | | | | | | | | | | | | | | 0 | | | | | | | | | | | | | | | | | | 0.0 | | | | | | | | | | | | | | | | | | | 1 | | | | | | | | | | | | | | | | | | | | | | | | | 0.5 | | | | | | | | | | | | | | | | | | |  | | | | | | | | | | | | | |
| **Walls cracked** (1missing wall type) | | | | | | | | | | | | | | | | | | | | | | | | | | | | | | | | | | | | | | | | | | | | | | | | | | | | | | | | | |  | | | | | | | |  | | | | | | | | | | | | | | | | | | | | | | | | | | | | | | |  | | | | | | | | | | | | | | | | | | |  | | | | | | | | | | | | | | | | | | | | | | |  | | | | | | | | | | | | | | | | | | 0.011 | | | | | | | | |
|  | | No (including walls not plastered at all) | | | | | | | | | | | | | | | | | | | | | | | | | | | | | | | | | | | | | | | | | | | | | | | | | | | | | | | | | | | | | | | | | | | | | | | 393 | | | | | | | | | | | | | | | 49.1 | | | | | | | | | | | | | | | | | | | | | | | | 82 | | | | | | | | | | | | | | | | | | | | | | | | | | 41.4 | | | | | | | | | | | | | | | | | | | |  | | | | | | | |
|  | | Yes, but not so many | | | | | | | | | | | | | | | | | | | | | | | | | | | | | | | | | | | | | | | | | | | | | | | | | | | | | | | | | | | | | | | | | | | | | | | 226 | | | | | | | | | | | | | | | 28.3 | | | | | | | | | | | | | | | | | | | | | | | | 51 | | | | | | | | | | | | | | | | | | | | | | | | | | 25.8 | | | | | | | | | | | | | | | | | | | |  | | | | | | | |
|  | | Yes, many | | | | | | | | | | | | | | | | | | | | | | | | | | | | | | | | | | | | | | | | | | | | | | | | | | | | | | | | | | | | | | | | | | | | | | | 181 | | | | | | | | | | | | | | | 22.6 | | | | | | | | | | | | | | | | | | | | | | | | 65 | | | | | | | | | | | | | | | | | | | | | | | | | | 32.8 | | | | | | | | | | | | | | | | | | | |  | | | | | | | |
| **Type of roof** | | | | | | | | | | Grass and wood | | | | | | | | | | | | | | | | | | | | | | | | | | | | | | | | | | | | | | | | | | | | | | | | | | | | | | | | | | | | | | | 778 | | | | | | | | | | | | | | | 97.3 | | | | | | | | | | | | | | | | | | | | | | | | 193 | | | | | | | | | | | | | | | | | | | | | | | | | | 97.5 | | | | | | | | | | | | | | | | | | | | 0.074 | | | | | | | |
| (1 missing) | | | | | | | | | | Cane/strong straw | | | | | | | | | | | | | | | | | | | | | | | | | | | | | | | | | | | | | | | | | | | | | | | | | | | | | | | | | | | | | | | 17 | | | | | | | | | | | | | | | 2.1 | | | | | | | | | | | | | | | | | | | | | | | | 1 | | | | | | | | | | | | | | | | | | | | | | | | | | 0.5 | | | | | | | | | | | | | | | | | | | |  | | | | | | | |
|  | | | | | | | | | | Metallic-zinc | | | | | | | | | | | | | | | | | | | | | | | | | | | | | | | | | | | | | | | | | | | | | | | | | | | | | | | | | | | | | | | 3 | | | | | | | | | | | | | | | 0.4 | | | | | | | | | | | | | | | | | | | | | | | | 3 | | | | | | | | | | | | | | | | | | | | | | | | | | 1.5 | | | | | | | | | | | | | | | | | | | |  | | | | | | | |
|  | | | | | | | | | | Other (plastic, strong mud) | | | | | | | | | | | | | | | | | | | | | | | | | | | | | | | | | | | | | | | | | | | | | | | | | | | | | | | | | | | | | | | 2 | | | | | | | | | | | | | | | 0.3 | | | | | | | | | | | | | | | | | | | | | | | | 1 | | | | | | | | | | | | | | | | | | | | | | | | | | 0.5 | | | | | | | | | | | | | | | | | | | |  | | | | | | | |
| **Type of floor** | | | | | | | | | | Earthen covered with gravel | | | | | | | | | | | | | | | | | | | | | | | | | | | | | | | | | | | | | | | | | | | | | | | | | | | | | | | | | | | | | | | 559 | | | | | | | | | | | | | | | 69.9 | | | | | | | | | | | | | | | | | | | | | | | | 155 | | | | | | | | | | | | | | | | | | | | | | | | | | 78.3 | | | | | | | | | | | | | | | | | | | | 0.013 | | | | | | | |
| (1 missing) | | | | | | | | | | Mixture based from animal dung | | | | | | | | | | | | | | | | | | | | | | | | | | | | | | | | | | | | | | | | | | | | | | | | | | | | | | | | | | | | | | | 94 | | | | | | | | | | | | | | | 11.8 | | | | | | | | | | | | | | | | | | | | | | | | 9 | | | | | | | | | | | | | | | | | | | | | | | | | | 4.6 | | | | | | | | | | | | | | | | | | | |  | | | | | | | |
|  | | | | | | | | | | Black cotton soil | | | | | | | | | | | | | | | | | | | | | | | | | | | | | | | | | | | | | | | | | | | | | | | | | | | | | | | | | | | | | | | 92 | | | | | | | | | | | | | | | 11.5 | | | | | | | | | | | | | | | | | | | | | | | | 23 | | | | | | | | | | | | | | | | | | | | | | | | | | 11.6 | | | | | | | | | | | | | | | | | | | |  | | | | | | | |
|  | | | | | | | | | | Sandy soil (azaza) | | | | | | | | | | | | | | | | | | | | | | | | | | | | | | | | | | | | | | | | | | | | | | | | | | | | | | | | | | | | | | | 42 | | | | | | | | | | | | | | | 5.3 | | | | | | | | | | | | | | | | | | | | | | | | 6 | | | | | | | | | | | | | | | | | | | | | | | | | | 3.0 | | | | | | | | | | | | | | | | | | | |  | | | | | | | |
|  | | | | | | | | | | Sand | | | | | | | | | | | | | | | | | | | | | | | | | | | | | | | | | | | | | | | | | | | | | | | | | | | | | | | | | | | | | | | 9 | | | | | | | | | | | | | | | 1.1 | | | | | | | | | | | | | | | | | | | | | | | | 5 | | | | | | | | | | | | | | | | | | | | | | | | | | 2.5 | | | | | | | | | | | | | | | | | | | |  | | | | | | | |
|  | | | | | | | | | | Carpet | | | | | | | | | | | | | | | | | | | | | | | | | | | | | | | | | | | | | | | | | | | | | | | | | | | | | | | | | | | | | | | 3 | | | | | | | | | | | | | | | 0.4 | | | | | | | | | | | | | | | | | | | | | | | | 0 | | | | | | | | | | | | | | | | | | | | | | | | | | 0.0 | | | | | | | | | | | | | | | | | | | |  | | | | | | | |
|  | | | | | | | | | | Rock, stone and gravel | | | | | | | | | | | | | | | | | | | | | | | | | | | | | | | | | | | | | | | | | | | | | | | | | | | | | | | | | | | | | | | 1 | | | | | | | | | | | | | | | 0.1 | | | | | | | | | | | | | | | | | | | | | | | | 0 | | | | | | | | | | | | | | | | | | | | | | | | | | 0.0 | | | | | | | | | | | | | | | | | | | |  | | | | | | | |
| **Type of windows** | | | | | | | | | | | | | | | | | | | | | | | | | No windows | | | | | | | | | | | | | | | | | | | | | | | | | | | | | | | | | | | | | | | | | | | | | | | | 540 | | | | | | | | | | | | | | | 67.5 | | | | | | | | | | | | | | | | | | | | | | | | 148 | | | | | | | | | | | | | | | | | | | | | | | | | | 74.8 | | | | | | | | | | | | | | | | | | | | 0.099 | | | | | | | |
| (1 missing) | | | | | | | | | | | | | | | | | | | | | | | | | Open, not protected window | | | | | | | | | | | | | | | | | | | | | | | | | | | | | | | | | | | | | | | | | | | | | | | | 157 | | | | | | | | | | | | | | | 19.6 | | | | | | | | | | | | | | | | | | | | | | | | 26 | | | | | | | | | | | | | | | | | | | | | | | | | | 13.1 | | | | | | | | | | | | | | | | | | | |  | | | | | | | |
|  | | | | | | | | | | | | | | | | | | | | | | | | | Windows with shutter | | | | | | | | | | | | | | | | | | | | | | | | | | | | | | | | | | | | | | | | | | | | | | | | 55 | | | | | | | | | | | | | | | 6.9 | | | | | | | | | | | | | | | | | | | | | | | | 16 | | | | | | | | | | | | | | | | | | | | | | | | | | 8.1 | | | | | | | | | | | | | | | | | | | |  | | | | | | | |
|  | | | | | | | | | | | | | | | | | | | | | | | | | Windows with screen/curtain | | | | | | | | | | | | | | | | | | | | | | | | | | | | | | | | | | | | | | | | | | | | | | | | 48 | | | | | | | | | | | | | | | 6.0 | | | | | | | | | | | | | | | | | | | | | | | | 8 | | | | | | | | | | | | | | | | | | | | | | | | | | 4.0 | | | | | | | | | | | | | | | | | | | |  | | | | | | | |
| **Termites in the room** | | | | | | | | | | | | | | | | | | | | | | | | | | | | | No | | | | | | | | | | | | | | | | | | | | | | | | | | | | | | | | | | | | | | | | | | | | 432 | | | | | | | | | | | | | | | 54.0 | | | | | | | | | | | | | | | | | | | | | | | | 118 | | | | | | | | | | | | | | | | | | | | | | | | | | 59.6 | | | | | | | | | | | | | | | | | | | | 0.080 | | | | | | | |
| (1 missing) | | | | | | | | | | | | | | | | | | | | | | | | | | | | | Yes, but not so many | | | | | | | | | | | | | | | | | | | | | | | | | | | | | | | | | | | | | | | | | | | | 203 | | | | | | | | | | | | | | | 25.4 | | | | | | | | | | | | | | | | | | | | | | | | 35 | | | | | | | | | | | | | | | | | | | | | | | | | | 17.7 | | | | | | | | | | | | | | | | | | | |  | | | | | | | |
|  | | | | | | | | | | | | | | | | | | | | | | | | | | | | | Yes, many | | | | | | | | | | | | | | | | | | | | | | | | | | | | | | | | | | | | | | | | | | | | 165 | | | | | | | | | | | | | | | 20.6 | | | | | | | | | | | | | | | | | | | | | | | | 45 | | | | | | | | | | | | | | | | | | | | | | | | | | 22.7 | | | | | | | | | | | | | | | | | | | |  | | | | | | | |
| **Floor made wet in the dry season** | | | | | | | | | | | | | | | | | | | | | | | | | | | | | | | | | | | | | | | | | | | | | | | | | | Daily | | | | | | | | | | | | | | | | | | | | | | | 34 | | | | | | | | | | | | | | | 4.3 | | | | | | | | | | | | | | | | | | | | | | | | 12 | | | | | | | | | | | | | | | | | | | | | | | | | | 6.1 | | | | | | | | | | | | | | | | | | | | 0.672 | | | | | | | |
| (2 missing) | | | | | | | | | | | | | | | | | | | | | | | | | | | | | | | | | | | | | | | | | | | | | | | | | | Frequently | | | | | | | | | | | | | | | | | | | | | | | 25 | | | | | | | | | | | | | | | 3.1 | | | | | | | | | | | | | | | | | | | | | | | | 6 | | | | | | | | | | | | | | | | | | | | | | | | | | 3.0 | | | | | | | | | | | | | | | | | | | |  | | | | | | | |
|  | | | | | | | | | | | | | | | | | | | | | | | | | | | | | | | | | | | | | | | | | | | | | | | | | | Sometimes | | | | | | | | | | | | | | | | | | | | | | | 243 | | | | | | | | | | | | | | | 30.4 | | | | | | | | | | | | | | | | | | | | | | | | 65 | | | | | | | | | | | | | | | | | | | | | | | | | | 32.8 | | | | | | | | | | | | | | | | | | | |  | | | | | | | |
|  | | | | | | | | | | | | | | | | | | | | | | | | | | | | | | | | | | | | | | | | | | | | | | | | | | Rarely | | | | | | | | | | | | | | | | | | | | | | | 40 | | | | | | | | | | | | | | | 5.0 | | | | | | | | | | | | | | | | | | | | | | | | 7 | | | | | | | | | | | | | | | | | | | | | | | | | | 3.5 | | | | | | | | | | | | | | | | | | | |  | | | | | | | |
|  | | | | | | | | | | | | | | | | | | | | | | | | | | | | | | | | | | | | | | | | | | | | | | | | | | Never | | | | | | | | | | | | | | | | | | | | | | | 457 | | | | | | | | | | | | | | | 57.2 | | | | | | | | | | | | | | | | | | | | | | | | 108 | | | | | | | | | | | | | | | | | | | | | | | | | | 54.6 | | | | | | | | | | | | | | | | | | | |  | | | | | | | |
| **Sand or gravels on the floor in the** | | | | | | | | | | | | | | | | | | | | | | | | | | | | | | | | | | | | | | | | | | | | | | | | | | Daily | | | | | | | | | | | | | | | | | | | | | | | 6 | | | | | | | | | | | | | | | 0.8 | | | | | | | | | | | | | | | | | | | | | | | | 2 | | | | | | | | | | | | | | | | | | | | | | | | | | 1.0 | | | | | | | | | | | | | | | | | | | | 0.034 | | | | | | | |
| **rainy season** | | | | | | | | | | | | | | | | | | | | | | | | | | | | | | | | | | | | | | | | | | | | | | | | | | Frequently | | | | | | | | | | | | | | | | | | | | | | | 77 | | | | | | | | | | | | | | | 9.6 | | | | | | | | | | | | | | | | | | | | | | | | 25 | | | | | | | | | | | | | | | | | | | | | | | | | | 12.6 | | | | | | | | | | | | | | | | | | | |  | | | | | | | |
| (1 missing) | | | | | | | | | | | | | | | | | | | | | | | | | | | | | | | | | | | | | | | | | | | | | | | | | | Sometimes | | | | | | | | | | | | | | | | | | | | | | | 278 | | | | | | | | | | | | | | | 34.8 | | | | | | | | | | | | | | | | | | | | | | | | 81 | | | | | | | | | | | | | | | | | | | | | | | | | | 40.9 | | | | | | | | | | | | | | | | | | | |  | | | | | | | |
|  | | | | | | | | | | | | | | | | | | | | | | | | | | | | | | | | | | | | | | | | | | | | | | | | | | Rarely | | | | | | | | | | | | | | | | | | | | | | | 142 | | | | | | | | | | | | | | | 17.8 | | | | | | | | | | | | | | | | | | | | | | | | 39 | | | | | | | | | | | | | | | | | | | | | | | | | | 19.7 | | | | | | | | | | | | | | | | | | | |  | | | | | | | |
|  | | | | | | | | | | | | | | | | | | | | | | | | | | | | | | | | | | | | | | | | | | | | | | | | | | Never | | | | | | | | | | | | | | | | | | | | | | | 297 | | | | | | | | | | | | | | | 37.1 | | | | | | | | | | | | | | | | | | | | | | | | 51 | | | | | | | | | | | | | | | | | | | | | | | | | | 25.8 | | | | | | | | | | | | | | | | | | | |  | | | | | | | |
| House (“tukul”) sprayed last year[[1]](#footnote-2) | | | | | | | | | | | | | | | | | | | | | | | | | | | | | | | | | | | | | | | | | | | | | | | | | | | | | | | | | | | | | | | | | | | | | | | | |  | | | | | | | | | | | | | | |  | | | | | | | | | | | | | | | | | | | | | | | |  | | | | | | | | | | | | | | | | | | | | | | | | | |  | | | | | | | | | | | | | | | | | | | | 0.036 | | | | | | | |
| (1 missing) | | | | | | | | | | | No | | | | | | | | | | | | | | | | | | | | | | | | | | | | | | | | | | | | | | | | | | | | | | | | | | | | | | | | | | | | | | 278 | | | | | | | | | | | | | | | 34.8 | | | | | | | | | | | | | | | | | | | | | | | | 51 | | | | | | | | | | | | | | | | | | | | | | | | | | 25.8 | | | | | | | | | | | | | | | | | | | |  | | | | | | | |
|  | | | | | | | | | | | Yes, by the household | | | | | | | | | | | | | | | | | | | | | | | | | | | | | | | | | | | | | | | | | | | | | | | | | | | | | | | | | | | | | | 58 | | | | | | | | | | | | | | | 7.3 | | | | | | | | | | | | | | | | | | | | | | | | 15 | | | | | | | | | | | | | | | | | | | | | | | | | | 7.6 | | | | | | | | | | | | | | | | | | | |  | | | | | | | |
|  | | | | | | | | | | | Yes, by MoH or other organisation | | | | | | | | | | | | | | | | | | | | | | | | | | | | | | | | | | | | | | | | | | | | | | | | | | | | | | | | | | | | | | 462 | | | | | | | | | | | | | | | 57.8 | | | | | | | | | | | | | | | | | | | | | | | | 130 | | | | | | | | | | | | | | | | | | | | | | | | | | 65.7 | | | | | | | | | | | | | | | | | | | |  | | | | | | | |
|  | | | | | | | | | | | Don’t know | | | | | | | | | | | | | | | | | | | | | | | | | | | | | | | | | | | | | | | | | | | | | | | | | | | | | | | | | | | | | | 2 | | | | | | | | | | | | | | | 0.3 | | | | | | | | | | | | | | | | | | | | | | | | 2 | | | | | | | | | | | | | | | | | | | | | | | | | | 1.0 | | | | | | | | | | | | | | | | | | | |  | | | | | | | |
| Fire or smoke as repellent indoor | | | | | | | | | | | | | | | | | | | | | | | | | | | | | | | | | | | | | | | | | | | Daily | | | | | | | | | | | | | | | | | | | | | | | | | | | | | 295 | | | | | | | | | | | | | | | | | | | 36.9 | | | | | | | | | | | | | | | | | | | | 92 | | | | | | | | | | | | | | | | | | | | | | | 47.2 | | | | | | | | | | | | | | | | | | 0.026 | | | | | | | | | | | | | |
| in the rainy season | | | | | | | | | | | | | | | | | | | | | | | | | | | | | | | | | | | | | | | | | | | Frequently | | | | | | | | | | | | | | | | | | | | | | | | | | | | | 34 | | | | | | | | | | | | | | | | | | | 4.3 | | | | | | | | | | | | | | | | | | | | 13 | | | | | | | | | | | | | | | | | | | | | | | 6.7 | | | | | | | | | | | | | | | | | |  | | | | | | | | | | | | | |
| (3 missing, 2 don't know) | | | | | | | | | | | | | | | | | | | | | | | | | | | | | | | | | | | | | | | | | | | Sometimes | | | | | | | | | | | | | | | | | | | | | | | | | | | | | 159 | | | | | | | | | | | | | | | | | | | 19.9 | | | | | | | | | | | | | | | | | | | | 30 | | | | | | | | | | | | | | | | | | | | | | | 15.4 | | | | | | | | | | | | | | | | | |  | | | | | | | | | | | | | |
|  | | | | | | | | | | | | | | | | | | | | | | | | | | | | | | | | | | | | | | | | | | | Rarely | | | | | | | | | | | | | | | | | | | | | | | | | | | | | 12 | | | | | | | | | | | | | | | | | | | 1.5 | | | | | | | | | | | | | | | | | | | | 3 | | | | | | | | | | | | | | | | | | | | | | | 1.5 | | | | | | | | | | | | | | | | | |  | | | | | | | | | | | | | |
|  | | | | | | | | | | | | | | | | | | | | | | | | | | | | | | | | | | | | | | | | | | | Never | | | | | | | | | | | | | | | | | | | | | | | | | | | | | 299 | | | | | | | | | | | | | | | | | | | 37.4 | | | | | | | | | | | | | | | | | | | | 57 | | | | | | | | | | | | | | | | | | | | | | | 29.2 | | | | | | | | | | | | | | | | | |  | | | | | | | | | | | | | |
| If yes, type of wood/material | | | | | | | | | | | | | | | | | | | | | | | | | | | | | | | | | | | | | | | | | | | (n=638) | | | | | | | | | | | | | | | | | | | | | | | | | | | | |  | | | | | | | | | | | | | | | | | | |  | | | | | | | | | | | | | | | | | | | |  | | | | | | | | | | | | | | | | | | | | | | |  | | | | | | | | | | | | | | | | | |  | | | | | | | | | | | | | |
|  | | | | | | Taleh (*Acacia seyal*) | | | | | | | | | | | | | | | | | | | | | | | | | | | | | | | | | | | | | | | | | | | | | | | | | | | | | | | | | | | | | | | | | | 434 | | | | | | | | | | | | | | | | | | | 86.8 | | | | | | | | | | | | | | | | | | | | 132 | | | | | | | | | | | | | | | | | | | | | | | 95.7 | | | | | | | | | | | | | | | | | | 0.004 | | | | | | | | | | | | | |
|  | | | | | | Neem (*Azadirachta indica*) | | | | | | | | | | | | | | | | | | | | | | | | | | | | | | | | | | | | | | | | | | | | | | | | | | | | | | | | | | | | | | | | | | 8 | | | | | | | | | | | | | | | | | | | 1.6 | | | | | | | | | | | | | | | | | | | | 1 | | | | | | | | | | | | | | | | | | | | | | | 0.7 | | | | | | | | | | | | | | | | | | 0.692 | | | | | | | | | | | | | |
|  | | | | | | Lalob/ higleeg (*Balanites aegyptica*) | | | | | | | | | | | | | | | | | | | | | | | | | | | | | | | | | | | | | | | | | | | | | | | | | | | | | | | | | | | | | | | | | | 9 | | | | | | | | | | | | | | | | | | | 1.8 | | | | | | | | | | | | | | | | | | | | 2 | | | | | | | | | | | | | | | | | | | | | | | 1.5 | | | | | | | | | | | | | | | | | | 1.000 | | | | | | | | | | | | | |
|  | | | | | | Droute(*Terminalia brownil*) | | | | | | | | | | | | | | | | | | | | | | | | | | | | | | | | | | | | | | | | | | | | | | | | | | | | | | | | | | | | | | | | | | 24 | | | | | | | | | | | | | | | | | | | 4.8 | | | | | | | | | | | | | | | | | | | | 4 | | | | | | | | | | | | | | | | | | | | | | | 2.9 | | | | | | | | | | | | | | | | | | 0.334 | | | | | | | | | | | | | |
|  | | | | | | Habeel (*Combretum hartimaninum*) | | | | | | | | | | | | | | | | | | | | | | | | | | | | | | | | | | | | | | | | | | | | | | | | | | | | | | | | | | | | | | | | | | 13 | | | | | | | | | | | | | | | | | | | 2.6 | | | | | | | | | | | | | | | | | | | | 5 | | | | | | | | | | | | | | | | | | | | | | | 3.6 | | | | | | | | | | | | | | | | | | 0.561 | | | | | | | | | | | | | |
|  | | | | | | Other | | | | | | | | | | | | | | | | | | | | | | | | | | | | | | | | | | | | | | | | | | | | | | | | | | | | | | | | | | | | | | | | | | 27 | | | | | | | | | | | | | | | | | | | 5.4 | | | | | | | | | | | | | | | | | | | | 0 | | | | | | | | | | | | | | | | | | | | | | | 0.0 | | | | | | | | | | | | | | | | | | 0.005 | | | | | | | | | | | | | |
| Fire or smoke as repellent indoor | | | | | | | | | | | | | | | | | | | | | | | | | | | | | | | | | | | | | | | | | | | Daily | | | | | | | | | | | | | | | | | | | | | | | | | | | | | 42 | | | | | | | | | | | | | | | | | | | 5.3 | | | | | | | | | | | | | | | | | | | | 17 | | | | | | | | | | | | | | | | | | | | | | | 8.7 | | | | | | | | | | | | | | | | | | 0.014 | | | | | | | | | | | | | |
| In the dry season | | | | | | | | | | | | | | | | | | | | | | | | | | | | | | | | | | | | | | | | | | | Frequently | | | | | | | | | | | | | | | | | | | | | | | | | | | | | 7 | | | | | | | | | | | | | | | | | | | 0.9 | | | | | | | | | | | | | | | | | | | | 3 | | | | | | | | | | | | | | | | | | | | | | | 1.5 | | | | | | | | | | | | | | | | | |  | | | | | | | | | | | | | |
| (3 missing, 2 don't know) | | | | | | | | | | | | | | | | | | | | | | | | | | | | | | | | | | | | | | | | | | | Sometimes | | | | | | | | | | | | | | | | | | | | | | | | | | | | | 107 | | | | | | | | | | | | | | | | | | | 13.4 | | | | | | | | | | | | | | | | | | | | 17 | | | | | | | | | | | | | | | | | | | | | | | 8.7 | | | | | | | | | | | | | | | | | |  | | | | | | | | | | | | | |
|  | | | | | | | | | | | | | | | | | | | | | | | | | | | | | | | | | | | | | | | | | | | Rarely | | | | | | | | | | | | | | | | | | | | | | | | | | | | | 19 | | | | | | | | | | | | | | | | | | | 2.4 | | | | | | | | | | | | | | | | | | | | 11 | | | | | | | | | | | | | | | | | | | | | | | 5.6 | | | | | | | | | | | | | | | | | |  | | | | | | | | | | | | | |
|  | | | | | | | | | | | | | | | | | | | | | | | | | | | | | | | | | | | | | | | | | | | Never | | | | | | | | | | | | | | | | | | | | | | | | | | | | | 624 | | | | | | | | | | | | | | | | | | | 78.1 | | | | | | | | | | | | | | | | | | | | 147 | | | | | | | | | | | | | | | | | | | | | | | 75.4 | | | | | | | | | | | | | | | | | |  | | | | | | | | | | | | | |
| If yes, type of wood/material | | | | | | | | | | | | | | | | | | | | | | | | | | | | | | | | | | | | | | | | | | | (n=223) | | | | | | | | | | | | | | | | | | | | | | | | | | | | |  | | | | | | | | | | | | | | | | | | |  | | | | | | | | | | | | | | | | | | | |  | | | | | | | | | | | | | | | | | | | | | | |  | | | | | | | | | | | | | | | | | |  | | | | | | | | | | | | | |
|  | | | | | | Taleh (*Acacia seyal*) | | | | | | | | | | | | | | | | | | | | | | | | | | | | | | | | | | | | | | | | | | | | | | | | | | | | | | | | | | | | | | | | | | 149 | | | | | | | | | | | | | | | | | | | 85.1 | | | | | | | | | | | | | | | | | | | | 45 | | | | | | | | | | | | | | | | | | | | | | | 93.7 | | | | | | | | | | | | | | | | | | 0.116 | | | | | | | | | | | | | |
|  | | | | | | Neem (*Azadirachta indica*) | | | | | | | | | | | | | | | | | | | | | | | | | | | | | | | | | | | | | | | | | | | | | | | | | | | | | | | | | | | | | | | | | | 0 | | | | | | | | | | | | | | | | | | | 0.0 | | | | | | | | | | | | | | | | | | | | 0 | | | | | | | | | | | | | | | | | | | | | | | 0.0 | | | | | | | | | | | | | | | | | | NA | | | | | | | | | | | | | |
|  | | | | | | Lalob/ higleeg (*Balanites aegyptica*) | | | | | | | | | | | | | | | | | | | | | | | | | | | | | | | | | | | | | | | | | | | | | | | | | | | | | | | | | | | | | | | | | | 3 | | | | | | | | | | | | | | | | | | | 1.7 | | | | | | | | | | | | | | | | | | | | 0 | | | | | | | | | | | | | | | | | | | | | | | 0.0 | | | | | | | | | | | | | | | | | | 1.000 | | | | | | | | | | | | | |
|  | | | | | | Droute(*Terminalia brownil*) | | | | | | | | | | | | | | | | | | | | | | | | | | | | | | | | | | | | | | | | | | | | | | | | | | | | | | | | | | | | | | | | | | 19 | | | | | | | | | | | | | | | | | | | 10.9 | | | | | | | | | | | | | | | | | | | | 2 | | | | | | | | | | | | | | | | | | | | | | | 4.2 | | | | | | | | | | | | | | | | | | 0.262 | | | | | | | | | | | | | |
|  | | | | | | Habeel (*Combretum hartimaninum*) | | | | | | | | | | | | | | | | | | | | | | | | | | | | | | | | | | | | | | | | | | | | | | | | | | | | | | | | | | | | | | | | | | 3 | | | | | | | | | | | | | | | | | | | 1.7 | | | | | | | | | | | | | | | | | | | | 3 | | | | | | | | | | | | | | | | | | | | | | | 6.3 | | | | | | | | | | | | | | | | | | 0.116 | | | | | | | | | | | | | |
|  | | | | | | Other | | | | | | | | | | | | | | | | | | | | | | | | | | | | | | | | | | | | | | | | | | | | | | | | | | | | | | | | | | | | | | | | | | 8 | | | | | | | | | | | | | | | | | | | 4.6 | | | | | | | | | | | | | | | | | | | | 0 | | | | | | | | | | | | | | | | | | | | | | | 0.0 | | | | | | | | | | | | | | | | | | 0.207 | | | | | | | | | | | | | |
| Thematic section (household level): Characteristics of the house yard and immediate surroundings | | | | | | | | | | | | | | | | | | | | | | | | | | | | | | | | | | | | | | | | | | | | | | | | | | | | | | | | | | | | | | | | | | | | | | | | | | | | | | | | | | | | | | | | | | | | | | | | | | | | | | | | | | | | | | | | | | | | | | | | | | | | | | | | | | | | | | | | | | | | | | | | | | | | | | | | | | | | | | | | | | | | | |
| **Constructions in the house yard** (yes *versus* no) | | | | | | | | | | | | | | | | | | | | | | | | | | | | | | | | | | | | | | | | | | | | | | | | | | | | | | | | | | | | | | | | | | | | | | | | |  | | | | | | | | | | | | | | | | | | | | | | | | |  | | | | | | | | | | | | | | | | | | | | |  | | | | | | | | | | | | | | | | | | | | |  | | | | | | | | | | | | | | | | | | |  | | | | | | |
|  | | Brick or cement room | | | | | | | | | | | | | | | | | | | | | | | | | | | | | | | | | | | | | | | | | | | | | | | | | | | | | | | | | | | | | | | | | | | | | | | 4 | | | | | | | | | | | | | | | | | | | | | | | | | 0.5 | | | | | | | | | | | | | | | | | | | | | 1 | | | | | | | | | | | | | | | | | | | | | 0.5 | | | | | | | | | | | | | | | | | | | 1.000 | | | | | | |
|  | | “Tukul” | | | | | | | | | | | | | | | | | | | | | | | | | | | | | | | | | | | | | | | | | | | | | | | | | | | | | | | | | | | | | | | | | | | | | | | 759 | | | | | | | | | | | | | | | | | | | | | | | | | 94.8 | | | | | | | | | | | | | | | | | | | | | 195 | | | | | | | | | | | | | | | | | | | | | 98.5 | | | | | | | | | | | | | | | | | | | 0.024 | | | | | | |
|  | | Local room | | | | | | | | | | | | | | | | | | | | | | | | | | | | | | | | | | | | | | | | | | | | | | | | | | | | | | | | | | | | | | | | | | | | | | | 86 | | | | | | | | | | | | | | | | | | | | | | | | | 10.7 | | | | | | | | | | | | | | | | | | | | | 8 | | | | | | | | | | | | | | | | | | | | | 4.0 | | | | | | | | | | | | | | | | | | | 0.004 | | | | | | |
|  | | Shelter | | | | | | | | | | | | | | | | | | | | | | | | | | | | | | | | | | | | | | | | | | | | | | | | | | | | | | | | | | | | | | | | | | | | | | | 717 | | | | | | | | | | | | | | | | | | | | | | | | | 89.5 | | | | | | | | | | | | | | | | | | | | | 170 | | | | | | | | | | | | | | | | | | | | | 85.9 | | | | | | | | | | | | | | | | | | | 0.144 | | | | | | |
|  | | Animal's accommodation (other than poultry) | | | | | | | | | | | | | | | | | | | | | | | | | | | | | | | | | | | | | | | | | | | | | | | | | | | | | | | | | | | | | | | | | | | | | | | 558 | | | | | | | | | | | | | | | | | | | | | | | | | 69.7 | | | | | | | | | | | | | | | | | | | | | 128 | | | | | | | | | | | | | | | | | | | | | 64.7 | | | | | | | | | | | | | | | | | | | 0.173 | | | | | | |
|  | | Poultry accommodation | | | | | | | | | | | | | | | | | | | | | | | | | | | | | | | | | | | | | | | | | | | | | | | | | | | | | | | | | | | | | | | | | | | | | | | 605 | | | | | | | | | | | | | | | | | | | | | | | | | 75.5 | | | | | | | | | | | | | | | | | | | | | 131 | | | | | | | | | | | | | | | | | | | | | 66.2 | | | | | | | | | | | | | | | | | | | 0.007 | | | | | | |
| **Crops in the yard** (yes *versus* no) | | | | | | | | | | | | | | | | | | | | | | | | | | | | | | | | | | | | | | | | | | | | | | | | | | | | | | | | | | | | | | | | | | | | | | | | | 117 | | | | | | | | | | | | | | | | | | | | | | | | | 14.6 | | | | | | | | | | | | | | | | | | | | | 29 | | | | | | | | | | | | | | | | | | | | | 14.7 | | | | | | | | | | | | | | | | | | | 0.989 | | | | | | |
| **Type of soil in the yard** | | | | | | | | | | | | | | | | | | | | | | | | | | | | | | | | | | | | | | | | | | | | | | | | | | | | | | | | | | | | | | | | | | | | | | | | |  | | | | | | | | | | | | | | | | | | | | | | | | |  | | | | | | | | | | | | | | | | | | | | |  | | | | | | | | | | | | | | | | | | | | |  | | | | | | | | | | | | | | | | | | | 0.001 | | | | | | |
|  | | Earthen covered with gravel | | | | | | | | | | | | | | | | | | | | | | | | | | | | | | | | | | | | | | | | | | | | | | | | | | | | | | | | | | | | | | | | | | | | | | | 450 | | | | | | | | | | | | | | | | | | | | | | | | | 56.2 | | | | | | | | | | | | | | | | | | | | | 132 | | | | | | | | | | | | | | | | | | | | | 66.7 | | | | | | | | | | | | | | | | | | |  | | | | | | |
|  | | Black cotton soil | | | | | | | | | | | | | | | | | | | | | | | | | | | | | | | | | | | | | | | | | | | | | | | | | | | | | | | | | | | | | | | | | | | | | | | 187 | | | | | | | | | | | | | | | | | | | | | | | | | 23.4 | | | | | | | | | | | | | | | | | | | | | 52 | | | | | | | | | | | | | | | | | | | | | 26.3 | | | | | | | | | | | | | | | | | | |  | | | | | | |
|  | | Sandy soil (azaza) | | | | | | | | | | | | | | | | | | | | | | | | | | | | | | | | | | | | | | | | | | | | | | | | | | | | | | | | | | | | | | | | | | | | | | | 140 | | | | | | | | | | | | | | | | | | | | | | | | | 17.5 | | | | | | | | | | | | | | | | | | | | | 13 | | | | | | | | | | | | | | | | | | | | | 6.6 | | | | | | | | | | | | | | | | | | |  | | | | | | |
|  | | Rock, stone and gravel | | | | | | | | | | | | | | | | | | | | | | | | | | | | | | | | | | | | | | | | | | | | | | | | | | | | | | | | | | | | | | | | | | | | | | | 10 | | | | | | | | | | | | | | | | | | | | | | | | | 1.3 | | | | | | | | | | | | | | | | | | | | | 0 | | | | | | | | | | | | | | | | | | | | | 0.0 | | | | | | | | | | | | | | | | | | |  | | | | | | |
|  | | Mixture based from animal dung | | | | | | | | | | | | | | | | | | | | | | | | | | | | | | | | | | | | | | | | | | | | | | | | | | | | | | | | | | | | | | | | | | | | | | | 7 | | | | | | | | | | | | | | | | | | | | | | | | | 0.9 | | | | | | | | | | | | | | | | | | | | | 0 | | | | | | | | | | | | | | | | | | | | | 0.0 | | | | | | | | | | | | | | | | | | |  | | | | | | |
|  | | Sand | | | | | | | | | | | | | | | | | | | | | | | | | | | | | | | | | | | | | | | | | | | | | | | | | | | | | | | | | | | | | | | | | | | | | | | 6 | | | | | | | | | | | | | | | | | | | | | | | | | 0.8 | | | | | | | | | | | | | | | | | | | | | 1 | | | | | | | | | | | | | | | | | | | | | 0.5 | | | | | | | | | | | | | | | | | | |  | | | | | | |
|  | | Other (sandy soil + black cotton) | | | | | | | | | | | | | | | | | | | | | | | | | | | | | | | | | | | | | | | | | | | | | | | | | | | | | | | | | | | | | | | | | | | | | | | 1 | | | | | | | | | | | | | | | | | | | | | | | | | 0.1 | | | | | | | | | | | | | | | | | | | | | 0 | | | | | | | | | | | | | | | | | | | | | 0.0 | | | | | | | | | | | | | | | | | | |  | | | | | | |
| **Type of soil in 10** | | | | | | | | | | | | | | | | | | | | | | | | | | Black cotton soil | | | | | | | | | | | | | | | | | | | | | | | | | | | | | | | | | | | | | | | | | | | | | | | | | 598 | | | | | | | | | | | | | | | | | | | | | | | | | 74.7 | | | | | | | | | | | | | | | | | | | | | | | 169 | | | | | | | | | | | | | | | | | | | | 85.4 | | | | | | | | | | | | | | | | | | | | <0.001 | | |
| **meters around** | | | | | | | | | | | | | | | | | | | | | | | | | | Earthen covered with gravel | | | | | | | | | | | | | | | | | | | | | | | | | | | | | | | | | | | | | | | | | | | | | | | | | 7 | | | | | | | | | | | | | | | | | | | | | | | | | 0.9 | | | | | | | | | | | | | | | | | | | | | | | 10 | | | | | | | | | | | | | | | | | | | | 5.1 | | | | | | | | | | | | | | | | | | | |  | | |
| **Yard** | | | | | | | | | | | | | | | | | | | | | | | | | | Sand | | | | | | | | | | | | | | | | | | | | | | | | | | | | | | | | | | | | | | | | | | | | | | | | | 5 | | | | | | | | | | | | | | | | | | | | | | | | | 0.6 | | | | | | | | | | | | | | | | | | | | | | | 1 | | | | | | | | | | | | | | | | | | | | 0.5 | | | | | | | | | | | | | | | | | | | |  | | |
|  | | | | | | | | | | | | | | | | | | | | | | | | | | Sandy soil (azaza) | | | | | | | | | | | | | | | | | | | | | | | | | | | | | | | | | | | | | | | | | | | | | | | | | 179 | | | | | | | | | | | | | | | | | | | | | | | | | 22.4 | | | | | | | | | | | | | | | | | | | | | | | 18 | | | | | | | | | | | | | | | | | | | | 9.1 | | | | | | | | | | | | | | | | | | | |  | | |
|  | | | | | | | | | | | | | | | | | | | | | | | | | | Rock, stone and gravel | | | | | | | | | | | | | | | | | | | | | | | | | | | | | | | | | | | | | | | | | | | | | | | | | 12 | | | | | | | | | | | | | | | | | | | | | | | | | 1.5 | | | | | | | | | | | | | | | | | | | | | | | 0 | | | | | | | | | | | | | | | | | | | | 0.0 | | | | | | | | | | | | | | | | | | | |  | | |
| **Termite hills in the house yard** (yes *versus* no) | | | | | | | | | | | | | | | | | | | | | | | | | | | | | | | | | | | | | | | | | | | | | | | | | | | | | | | | | | | | | | | | | | | | | | | | | | | 94 | | | | | | | | | | | | | | | | | | | | | | | | | 11.7 | | | | | | | | | | | | | | | | | | | | | | | 11 | | | | | | | | | | | | | | | | | | | | 5.6 | | | | | | | | | | | | | | | | | | | | 0.011 | | |
| **Termite hills in the immediate surroundings** | | | | | | | | | | | | | | | | | | | | | | | | | | | | | | | | | | | | | | | | | | | | | | | | | | | | | | | | | | | | | | | | | | | | | | | | | | | 232 | | | | | | | | | | | | | | | | | | | | | | | | | 29.0 | | | | | | | | | | | | | | | | | | | | | | | 47 | | | | | | | | | | | | | | | | | | | | 23.7 | | | | | | | | | | | | | | | | | | | | 0.142 | | |
| **Trees cut in the yard over the past year[[2]](#footnote-3)** | | | | | | | | | | | | | | | | | | | | | | | | | | | | | | | | | | | | | | | | | | | | | | | | | | | | | | | | | | | | | | | | | | | | | | | | | | | 199 | | | | | | | | | | | | | | | | | | | | | | | | | 24.8 | | | | | | | | | | | | | | | | | | | | | | | 67 | | | | | | | | | | | | | | | | | | | | 33.8 | | | | | | | | | | | | | | | | | | | | 0.010 | | |
| **Trees in the yard** | | | | | | | | | | | | | | | | | | | | | | | | | | | | | | | | | | | | | | | | | | | | | | | | | | | | | | | | | | | | | | | | | | | | | | | | | | |  | | | | | | | | | | | | | | | | | | | | | | | | |  | | | | | | | | | | | | | | | | | | | | | | |  | | | | | | | | | | | | | | | | | | | |  | | | | | | | | | | | | | | | | | | | |  | | |
| Neem *(Azadirachta indica)* | | | | | | | | | | | | | | | | | | | | | | | | | | | | | | | | | | | | | | | | | | | | | | | | | | | *(≥1 )* | | | | | | | | | | | | | | | | | | | | | | | 464 | | | | | | | | | | | | | | | | | | | | | | | | | 57.9 | | | | | | | | | | | | | | | | | | | | | | | 103 | | | | | | | | | | | | | | | | | | | | 52.0 | | | | | | | | | | | | | | | | | | | 0.133 | | | | |
| Sidr *(Ziziphus spina-christi)* | | | | | | | | | | | | | | | | | | | | | | | | | | | | | | | | | | | | | | | | | | | | | | | | | | | *(≥1 )* | | | | | | | | | | | | | | | | | | | | | | | 398 | | | | | | | | | | | | | | | | | | | | | | | | | 49.7 | | | | | | | | | | | | | | | | | | | | | | | 113 | | | | | | | | | | | | | | | | | | | | 57.1 | | | | | | | | | | | | | | | | | | | 0.063 | | | | |
| Lalob/higleeg *(Balanites aegyptiaca)* | | | | | | | | | | | | | | | | | | | | | | | | | | | | | | | | | | | | | | | | | | | | | | | | | | | *(≥1 )* | | | | | | | | | | | | | | | | | | | | | | | 387 | | | | | | | | | | | | | | | | | | | | | | | | | 48.3 | | | | | | | | | | | | | | | | | | | | | | | 100 | | | | | | | | | | | | | | | | | | | | 50.5 | | | | | | | | | | | | | | | | | | | 0.581 | | | | |
| Sonot/Garat *(Acacia nilotica)* | | | | | | | | | | | | | | | | | | | | | | | | | | | | | | | | | | | | | | | | | | | | | | | | | | | *(≥1 )* | | | | | | | | | | | | | | | | | | | | | | | 137 | | | | | | | | | | | | | | | | | | | | | | | | | 17.1 | | | | | | | | | | | | | | | | | | | | | | | 47 | | | | | | | | | | | | | | | | | | | | 23.7 | | | | | | | | | | | | | | | | | | | 0.031 | | | | |
| Hashab *(Acacia senegal)* | | | | | | | | | | | | | | | | | | | | | | | | | | | | | | | | | | | | | | | | | | | | | | | | | | | *(≥1 )* | | | | | | | | | | | | | | | | | | | | | | | 90 | | | | | | | | | | | | | | | | | | | | | | | | | 11.2 | | | | | | | | | | | | | | | | | | | | | | | 34 | | | | | | | | | | | | | | | | | | | | 17.2 | | | | | | | | | | | | | | | | | | | 0.023 | | | | |
| Taleh *(Acacia seyal)* | | | | | | | | | | | | | | | | | | | | | | | | | | | | | | | | | | | | | | | | | | | | | | | | | | | *(≥1 )* | | | | | | | | | | | | | | | | | | | | | | | 79 | | | | | | | | | | | | | | | | | | | | | | | | | 9.9 | | | | | | | | | | | | | | | | | | | | | | | 26 | | | | | | | | | | | | | | | | | | | | 13.1 | | | | | | | | | | | | | | | | | | | 0.179 | | | | |
| Kiter *(Acacia Mellifera)* | | | | | | | | | | | | | | | | | | | | | | | | | | | | | | | | | | | | | | | | | | | | | | | | | | | *(≥1 )* | | | | | | | | | | | | | | | | | | | | | | | 46 | | | | | | | | | | | | | | | | | | | | | | | | | 5.7 | | | | | | | | | | | | | | | | | | | | | | | 15 | | | | | | | | | | | | | | | | | | | | 7.6 | | | | | | | | | | | | | | | | | | | 0.335 | | | | |
| Other trees | | | | | | | | | | | | | | | | | | | | | | | | | | | | | | | | | | | | | | | | | | | | | | | | | | | *(≥1 )* | | | | | | | | | | | | | | | | | | | | | | | 210 | | | | | | | | | | | | | | | | | | | | | | | | | 26.2 | | | | | | | | | | | | | | | | | | | | | | | 44 | | | | | | | | | | | | | | | | | | | | 22.2 | | | | | | | | | | | | | | | | | | | 0.248 | | | | |
| **Trees in the immediate surroundings/around the yard** | | | | | | | | | | | | | | | | | | | | | | | | | | | | | | | | | | | | | | | | | | | | | | | | | | | | | | | | | | | | | | | | | | | | | | | | | | | | | | | | | | | | | | | | | | | | | | | | | | | | | | | | | | | | | | | | | | | | | | | | | | | |  | | | | | | | | | | | | | | | | | | | |  | | | | | | | | | | | | | | | | | | | |  | |
| Neem *(Azadirachta indica)* | | | | | | | | | | | | | | | | | | | | | | | | | | | | | | | | | | | | | | | | | | | | | | | | | | | *(≥1 )* | | | | | | | | | | | | | | | | | | | | | | | | | 695 | | | | | | | | | | | | | | | | | | | | | | | | | | 87.0 | | | | | | | | | | | | | | | | | | | | | | 181 | | | | | | | | | | | | | | | | | | | | 91.4 | | | | | | | | | | | | | | | | | | | | 0.087 | |
| Sidr *(Ziziphus spina-christi)* | | | | | | | | | | | | | | | | | | | | | | | | | | | | | | | | | | | | | | | | | | | | | | | | | | | *(≥1 )* | | | | | | | | | | | | | | | | | | | | | | | | | 668 | | | | | | | | | | | | | | | | | | | | | | | | | | 83.5 | | | | | | | | | | | | | | | | | | | | | | 178 | | | | | | | | | | | | | | | | | | | | 89.9 | | | | | | | | | | | | | | | | | | | | 0.025 | |
| Lalob/higleeg *(Balanites aegyptiaca)* | | | | | | | | | | | | | | | | | | | | | | | | | | | | | | | | | | | | | | | | | | | | | | | | | | | *(≥1 )* | | | | | | | | | | | | | | | | | | | | | | | | | 694 | | | | | | | | | | | | | | | | | | | | | | | | | | 86.5 | | | | | | | | | | | | | | | | | | | | | | 186 | | | | | | | | | | | | | | | | | | | | 93.9 | | | | | | | | | | | | | | | | | | | | 0.003 | |
| Sonot/Garat *(Acacia nilotica)* | | | | | | | | | | | | | | | | | | | | | | | | | | | | | | | | | | | | | | | | | | | | | | | | | | | *(≥1 )* | | | | | | | | | | | | | | | | | | | | | | | | | 348 | | | | | | | | | | | | | | | | | | | | | | | | | | 43.5 | | | | | | | | | | | | | | | | | | | | | | 125 | | | | | | | | | | | | | | | | | | | | 63.1 | | | | | | | | | | | | | | | | | | | | <0.001 | |
| Hashab *(Acacia senegal)* | | | | | | | | | | | | | | | | | | | | | | | | | | | | | | | | | | | | | | | | | | | | | | | | | | | *(≥1 )* | | | | | | | | | | | | | | | | | | | | | | | | | 321 | | | | | | | | | | | | | | | | | | | | | | | | | | 40.1 | | | | | | | | | | | | | | | | | | | | | | 97 | | | | | | | | | | | | | | | | | | | | 49.0 | | | | | | | | | | | | | | | | | | | | 0.024 | |
| Taleh *(Acacia seyal)* | | | | | | | | | | | | | | | | | | | | | | | | | | | | | | | | | | | | | | | | | | | | | | | | | | | *(≥1 )* | | | | | | | | | | | | | | | | | | | | | | | | | 264 | | | | | | | | | | | | | | | | | | | | | | | | | | 33.0 | | | | | | | | | | | | | | | | | | | | | | 88 | | | | | | | | | | | | | | | | | | | | 44.4 | | | | | | | | | | | | | | | | | | | | 0.003 | |
| Kiter *(Acacia Mellifera)* | | | | | | | | | | | | | | | | | | | | | | | | | | | | | | | | | | | | | | | | | | | | | | | | | | | *(≥1 )* | | | | | | | | | | | | | | | | | | | | | | | | | 183 | | | | | | | | | | | | | | | | | | | | | | | | | | 22.9 | | | | | | | | | | | | | | | | | | | | | | 71 | | | | | | | | | | | | | | | | | | | | 35.9 | | | | | | | | | | | | | | | | | | | | <0.001 | |
| Other trees | | | | | | | | | | | | | | | | | | | | | | | | | | | | | | | | | | | | | | | | | | | | | | | | | | | *(≥1 )* | | | | | | | | | | | | | | | | | | | | | | | | | 226 | | | | | | | | | | | | | | | | | | | | | | | | | | 28.3 | | | | | | | | | | | | | | | | | | | | | | 53 | | | | | | | | | | | | | | | | | | | | 26.8 | | | | | | | | | | | | | | | | | | | | 0.677 | |
| **Forest/wood at eye range** | | | | | | | | | | | | | | | | | | | | | | | | | | | | | | | | | | | | | | | | No | | | | | | | | | | | | | | | | | | | | | | | | | | | | | | | | | | | | 544 | | | | | | | | | | | | | | | | | | | | | | | | | | 68.1 | | | | | | | | | | | | | | | | | | | | | | 118 | | | | | | | | | | | | | | | | | | | | 59.6 | | | | | | | | | | | | | | | | | | | | 0.066 | |
|  | | | | | | | | | | | | | | | | | | | | | | | | | | | | | | | | | | | | | | | | Yes, small forest | | | | | | | | | | | | | | | | | | | | | | | | | | | | | | | | | | | | 237 | | | | | | | | | | | | | | | | | | | | | | | | | | 29.7 | | | | | | | | | | | | | | | | | | | | | | 74 | | | | | | | | | | | | | | | | | | | | 37.4 | | | | | | | | | | | | | | | | | | | |  | |
|  | | | | | | | | | | | | | | | | | | | | | | | | | | | | | | | | | | | | | | | | Yes, dense forest | | | | | | | | | | | | | | | | | | | | | | | | | | | | | | | | | | | | 18 | | | | | | | | | | | | | | | | | | | | | | | | | | 2.3 | | | | | | | | | | | | | | | | | | | | | | 6 | | | | | | | | | | | | | | | | | | | | 3.0 | | | | | | | | | | | | | | | | | | | |  | |
| **Distance to the closest house yard** | | | | | | | | | | | | | | | | | | | | | | | | | | | | | | | | | | | | | | | | | | | | | | | | | | | | | | | | | | | | | | | | | | | | | | | | | | | |  | | | | | | | | | | | | | | | | | | | | | | | | | |  | | | | | | | | | | | | | | | | | | | | | |  | | | | | | | | | | | | | | | | | | | |  | | | | | | | | | | | | | | | | | | | | <0.001 | |
|  | | | | | | | | | | | | | | Share a common limit | | | | | | | | | | | | | | | | | | | | | | | | | | | | | | | | | | | | | | | | | | | | | | | | | | | | | | | | | | | | | | 591 | | | | | | | | | | | | | | | | | | | | | | | | | | 73.9 | | | | | | | | | | | | | | | | | | | | | | 169 | | | | | | | | | | | | | | | | | | | | 85.4 | | | | | | | | | | | | | | | | | | | |  | |
|  | | | | | | | | | | | | | | Space in between (<10 m) | | | | | | | | | | | | | | | | | | | | | | | | | | | | | | | | | | | | | | | | | | | | | | | | | | | | | | | | | | | | | | 119 | | | | | | | | | | | | | | | | | | | | | | | | | | 14.9 | | | | | | | | | | | | | | | | | | | | | | 14 | | | | | | | | | | | | | | | | | | | | 7.1 | | | | | | | | | | | | | | | | | | | |  | |
|  | | | | | | | | | | | | | | From 10 to 100m | | | | | | | | | | | | | | | | | | | | | | | | | | | | | | | | | | | | | | | | | | | | | | | | | | | | | | | | | | | | | | 85 | | | | | | | | | | | | | | | | | | | | | | | | | | 10.6 | | | | | | | | | | | | | | | | | | | | | | 10 | | | | | | | | | | | | | | | | | | | | 5.1 | | | | | | | | | | | | | | | | | | | |  | |
|  | | | | | | | | | | | | | | More than 100 m | | | | | | | | | | | | | | | | | | | | | | | | | | | | | | | | | | | | | | | | | | | | | | | | | | | | | | | | | | | | | | 5 | | | | | | | | | | | | | | | | | | | | | | | | | | 0.6 | | | | | | | | | | | | | | | | | | | | | | 5 | | | | | | | | | | | | | | | | | | | | 2.5 | | | | | | | | | | | | | | | | | | | |  | |
| Thematic section (household level): Animals and water bodies in the yard **in the rainy season** | | | | | | | | | | | | | | | | | | | | | | | | | | | | | | | | | | | | | | | | | | | | | | | | | | | | | | | | | | | | | | | | | | | | | | | | | | | | | | | | | | | | | | | | | | | | | | | | | | | | | | | | | | | | | | | | | | | | | | | | | | | | | | | | | | | | | | | | | | | | | | | | | | | | | | | | | | | | | | | | | | | | | |
| Animals in room at night *(mainly poultry, dog, goat)* | | | | | | | | | | | | | | | | | | | | | | | | | | | | | | | | | | | | | | | | | | | | | | | | | | | | | | | | | | | | | | | | | | | | | | | | | | | | 143 | | | | | | | | | | | | | | | | | | | | | | | | | | 17.9 | | | | | | | | | | | | | | | | | | | | | | 38 | | | | | | | | | | | | | | | | | | | | 19.2 | | | | | | | | | | | | | | | | | | | | 0.661 | |
| Rats/rodents in the room at night | | | | | | | | | | | | | | | | | | | | | | | | | | | | | | | | | | | | | | | | | | | | | | | | | | | | | | | | | | | | | | | | | | | | | | | | | | | | 310 | | | | | | | | | | | | | | | | | | | | | | | | | | 38.7 | | | | | | | | | | | | | | | | | | | | | | 71 | | | | | | | | | | | | | | | | | | | | 35.9 | | | | | | | | | | | | | | | | | | | | 0.461 | |
| Animal burrows in room at night | | | | | | | | | | | | | | | | | | | | | | | | | | | | | | | | | | | | | | | | | | | | | | | | No | | | | | | | | | | | | | | | | | | | | | | | | | | | | | 489 | | | | | | | | | | | | | | | | | | | | | | | | | | 61.1 | | | | | | | | | | | | | | | | | | | | | | | 127 | | | | | | | | | | | | | | | | | | | 64.1 | | | | | | | | | | | | | | | | | | | | 0.599 |
|  | | | | | | | | | | | | | | | | | | | | | | | | | | | | | | | | | | | | | | | | | | | | | | | | Yes, not many | | | | | | | | | | | | | | | | | | | | | | | | | | | | | 109 | | | | | | | | | | | | | | | | | | | | | | | | | | 13.6 | | | | | | | | | | | | | | | | | | | | | | | 22 | | | | | | | | | | | | | | | | | | | 11.1 | | | | | | | | | | | | | | | | | | | |  |
|  | | | | | | | | | | | | | | | | | | | | | | | | | | | | | | | | | | | | | | | | | | | | | | | | Yes, many | | | | | | | | | | | | | | | | | | | | | | | | | | | | | 203 | | | | | | | | | | | | | | | | | | | | | | | | | | 25.3 | | | | | | | | | | | | | | | | | | | | | | | 49 | | | | | | | | | | | | | | | | | | | 24.8 | | | | | | | | | | | | | | | | | | | |  |
| Animals in the yard at night | | | | | | | | | | | | | | | | | | | | | | | | | | | | | | | | | | | | | | | | | (yes *versus* no) | | | | | | | | | | | | | | | | | | | | | | | | | | | | | | | | | | | | 688 | | | | | | | | | | | | | | | | | | | | | | | | | | 86.0 | | | | | | | | | | | | | | | | | | | | | | | 158 | | | | | | | | | | | | | | | | | | | 79.8 | | | | | | | | | | | | | | | | | | | | 0.029 |
|  | | | | | | | | | | | | | | | | *Donkey* | | | | | | | | | | | | | | | | | | | | | | | | | | | | | | | | | | | | | | | | | | | | | | | | | | | | | | | | | | | | | *553* | | | | | | | | | | | | | | | | | | | | | | | | | | *69.0* | | | | | | | | | | | | | | | | | | | | | | | *135* | | | | | | | | | | | | | | | | | | | *68.2* | | | | | | | | | | | | | | | | | | | | *0.816* |
|  | | | | | | | | | | | | | | | | *Goat* | | | | | | | | | | | | | | | | | | | | | | | | | | | | | | | | | | | | | | | | | | | | | | | | | | | | | | | | | | | | | *326* | | | | | | | | | | | | | | | | | | | | | | | | | | *40.7* | | | | | | | | | | | | | | | | | | | | | | | *69* | | | | | | | | | | | | | | | | | | | *34.9* | | | | | | | | | | | | | | | | | | | | *0.132* |
|  | | | | | | | | | | | | | | | | *Sheep* | | | | | | | | | | | | | | | | | | | | | | | | | | | | | | | | | | | | | | | | | | | | | | | | | | | | | | | | | | | | | *296* | | | | | | | | | | | | | | | | | | | | | | | | | | *37.0* | | | | | | | | | | | | | | | | | | | | | | | *59* | | | | | | | | | | | | | | | | | | | *29.8* | | | | | | | | | | | | | | | | | | | | *0.060* |
|  | | | | | | | | | | | | | | | | *Cattle* | | | | | | | | | | | | | | | | | | | | | | | | | | | | | | | | | | | | | | | | | | | | | | | | | | | | | | | | | | | | | *159* | | | | | | | | | | | | | | | | | | | | | | | | | | *19.9* | | | | | | | | | | | | | | | | | | | | | | | *32* | | | | | | | | | | | | | | | | | | | *16.2* | | | | | | | | | | | | | | | | | | | | *0.237* |
|  | | | | | | | | | | | | | | | | *Dog* | | | | | | | | | | | | | | | | | | | | | | | | | | | | | | | | | | | | | | | | | | | | | | | | | | | | | | | | | | | | | *55* | | | | | | | | | | | | | | | | | | | | | | | | | | *6.9* | | | | | | | | | | | | | | | | | | | | | | | *21* | | | | | | | | | | | | | | | | | | | *10.6* | | | | | | | | | | | | | | | | | | | | *0.088* |
|  | | | | | | | | | | | | | | | | *Camel* | | | | | | | | | | | | | | | | | | | | | | | | | | | | | | | | | | | | | | | | | | | | | | | | | | | | | | | | | | | | | *4* | | | | | | | | | | | | | | | | | | | | | | | | | | *0.5* | | | | | | | | | | | | | | | | | | | | | | | *0* | | | | | | | | | | | | | | | | | | | *0.0* | | | | | | | | | | | | | | | | | | | | *1.000* |
| Animals in adjacent yard at night(1 don't know) | | | | | | | | | | | | | | | | | | | | | | | | | | | | | | | | | | | | | | | | | | | | | | | | | | | | | | | | | | | | | | | | |  | | | | | | | | | | | | 749 | | | | | | | | | | | | | | | | | | | | | | | | | | 93.5 | | | | | | | | | | | | | | | | | | | | | | | 179 | | | | | | | | | | | | | | | | | | | 90.4 | | | | | | | | | | | | | | | | | | | | 0.112 |
|  | | | | | | | | | | | | | | | | Donkey | | | | | | | | | | | | | | | | | | | | | | | | | | | | | | | | | | | | | | | | | | | | | | | | | | | | | | | | | | | | | *724* | | | | | | | | | | | | | | | | | | | | | | | | | | *90.4* | | | | | | | | | | | | | | | | | | | | | | | *169* | | | | | | | | | | | | | | | | | | | *85.4* | | | | | | | | | | | | | | | | | | | | *0.039* |
|  | | | | | | | | | | | | | | | | Goat | | | | | | | | | | | | | | | | | | | | | | | | | | | | | | | | | | | | | | | | | | | | | | | | | | | | | | | | | | | | | *596* | | | | | | | | | | | | | | | | | | | | | | | | | | *74.4* | | | | | | | | | | | | | | | | | | | | | | | *137* | | | | | | | | | | | | | | | | | | | *69.2* | | | | | | | | | | | | | | | | | | | | *0.137* |
|  | | | | | | | | | | | | | | | | Sheep | | | | | | | | | | | | | | | | | | | | | | | | | | | | | | | | | | | | | | | | | | | | | | | | | | | | | | | | | | | | | *520* | | | | | | | | | | | | | | | | | | | | | | | | | | *64.9* | | | | | | | | | | | | | | | | | | | | | | | *126* | | | | | | | | | | | | | | | | | | | *63.6* | | | | | | | | | | | | | | | | | | | | *0.735* |
|  | | | | | | | | | | | | | | | | Cattle | | | | | | | | | | | | | | | | | | | | | | | | | | | | | | | | | | | | | | | | | | | | | | | | | | | | | | | | | | | | | *317* | | | | | | | | | | | | | | | | | | | | | | | | | | *39.6* | | | | | | | | | | | | | | | | | | | | | | | *78* | | | | | | | | | | | | | | | | | | | *39.4* | | | | | | | | | | | | | | | | | | | | *0.963* |
|  | | | | | | | | | | | | | | | | Dog | | | | | | | | | | | | | | | | | | | | | | | | | | | | | | | | | | | | | | | | | | | | | | | | | | | | | | | | | | | | | *151* | | | | | | | | | | | | | | | | | | | | | | | | | | *18.9* | | | | | | | | | | | | | | | | | | | | | | | *46* | | | | | | | | | | | | | | | | | | | *23.2* | | | | | | | | | | | | | | | | | | | | *0.165* |
|  | | | | | | | | | | | | | | | | Camel | | | | | | | | | | | | | | | | | | | | | | | | | | | | | | | | | | | | | | | | | | | | | | | | | | | | | | | | | | | | | *13* | | | | | | | | | | | | | | | | | | | | | | | | | | *1.6* | | | | | | | | | | | | | | | | | | | | | | | *1* | | | | | | | | | | | | | | | | | | | *0.5* | | | | | | | | | | | | | | | | | | | | *0.231* |
|  | | | | | | | | | | | | | | | | Horse | | | | | | | | | | | | | | | | | | | | | | | | | | | | | | | | | | | | | | | | | | | | | | | | | | | | | | | | | | | | | *1* | | | | | | | | | | | | | | | | | | | | | | | | | | *0.1* | | | | | | | | | | | | | | | | | | | | | | | *0* | | | | | | | | | | | | | | | | | | | *0.0* | | | | | | | | | | | | | | | | | | | | *1* |
| Uncovered animal burrows in the yard | | | | | | | | | | | | | | | | | | | | | | | | | | | | | | | | | | | | | | | | | | | | | | | | | | | | | | | | | | | | | | | | | | | | | | | | | | | | |  | | | | | | | | | | | | | | | | | | | | | | | | | |  | | | | | | | | | | | | | | | | | | | | | | |  | | | | | | | | | | | | | | | | | | |  | | | | | | | | | | | | | | | | | | | | 0.165 |
| (2 don't know) | | | | | | | | | | | | | | | | No | | | | | | | | | | | | | | | | | | | | | | | | | | | | | | | | | | | | | | | | | | | | | | | | | | | | | | | | | | | | | 325 | | | | | | | | | | | | | | | | | | | | | | | | | | 40.7 | | | | | | | | | | | | | | | | | | | | | | | 75 | | | | | | | | | | | | | | | | | | | 37.9 | | | | | | | | | | | | | | | | | | | |  |
|  | | | | | | | | | | | | | | | | Yes, but not many | | | | | | | | | | | | | | | | | | | | | | | | | | | | | | | | | | | | | | | | | | | | | | | | | | | | | | | | | | | | | 71 | | | | | | | | | | | | | | | | | | | | | | | | | | 8.9 | | | | | | | | | | | | | | | | | | | | | | | 11 | | | | | | | | | | | | | | | | | | | 5.6 | | | | | | | | | | | | | | | | | | | |  |
|  | | | | | | | | | | | | | | | | Yes, many | | | | | | | | | | | | | | | | | | | | | | | | | | | | | | | | | | | | | | | | | | | | | | | | | | | | | | | | | | | | | 403 | | | | | | | | | | | | | | | | | | | | | | | | | | 50.4 | | | | | | | | | | | | | | | | | | | | | | | 112 | | | | | | | | | | | | | | | | | | | 56.6 | | | | | | | | | | | | | | | | | | | |  |
| **Use of repellent for the body of animals (if animals in the yard) in the rainy season** | | | | | | | | | | | | | | | | | | | | | | | | | | | | | | | | | | | | | | | | | | | | | | | | | | | | | | | | | | | | | | | | | | | | | | | | | | | | | | | | | | | | | | | | | | | | | | | | | | | | | | | | | | | | | | | | | | | | | | | | | | | | | | | | | | | | | | | | | | | | | | | | | | | | | | | | | | | | | | | | | | | | | |
| (n=795) | | | | | | | | | | | | | | | | | | | | | | Daily | | | | | | | | | | | | | | | | | | | | | | | | | | | | | | | | | | | | | | | | | | | | | | | | | | | | | | | | | 30 | | | | | | | | | | | | | | | | | | | | | | | | | | 4.7 | | | | | | | | | | | | | | | | | | | | | | 10 | | | | | | | | | | | | | | | | | | 6.7 | | | | | | | | | | | | | | | | | 0.189 | | | |
|  | | | | | | | | | | | | | | | | | | | | | | Frequently | | | | | | | | | | | | | | | | | | | | | | | | | | | | | | | | | | | | | | | | | | | | | | | | | | | | | | | | | 58 | | | | | | | | | | | | | | | | | | | | | | | | | | 9.0 | | | | | | | | | | | | | | | | | | | | | | 20 | | | | | | | | | | | | | | | | | | 13.3 | | | | | | | | | | | | | | | | |  | | | |
|  | | | | | | | | | | | | | | | | | | | | | | Sometimes | | | | | | | | | | | | | | | | | | | | | | | | | | | | | | | | | | | | | | | | | | | | | | | | | | | | | | | | | 268 | | | | | | | | | | | | | | | | | | | | | | | | | | 41.6 | | | | | | | | | | | | | | | | | | | | | | 65 | | | | | | | | | | | | | | | | | | 43.3 | | | | | | | | | | | | | | | | |  | | | |
|  | | | | | | | | | | | | | | | | | | | | | | Rarely | | | | | | | | | | | | | | | | | | | | | | | | | | | | | | | | | | | | | | | | | | | | | | | | | | | | | | | | | 21 | | | | | | | | | | | | | | | | | | | | | | | | | | 3.3 | | | | | | | | | | | | | | | | | | | | | | 2 | | | | | | | | | | | | | | | | | | 1.3 | | | | | | | | | | | | | | | | |  | | | |
|  | | | | | | | | | | | | | | | | | | | | | | Never | | | | | | | | | | | | | | | | | | | | | | | | | | | | | | | | | | | | | | | | | | | | | | | | | | | | | | | | | 268 | | | | | | | | | | | | | | | | | | | | | | | | | | 41.6 | | | | | | | | | | | | | | | | | | | | | | 53 | | | | | | | | | | | | | | | | | | 35.3 | | | | | | | | | | | | | | | | |  | | | |
| Type of animal repellent | | | | | | | | | | | | | | | | | | | | | | | | | | | | | | | | | | | | Chemical | | | | | | | | | | | | | | | | | | | | | | | | | | | | | | | | | | | | | | | | | | | 171 | | | | | | | | | | | | | | | | | | | | | | | | | | 44.9 | | | | | | | | | | | | | | | | | | | | | | 41 | | | | | | | | | | | | | | | | | | 42.3 | | | | | | | | | | | | | | | | | 0.644 | | | |
| (n=478) | | | | | | | | | | | | | | | | | | | | | | | | | | | | | | | | | | | | Local ‘Tar’ (Quotran) | | | | | | | | | | | | | | | | | | | | | | | | | | | | | | | | | | | | | | | | | | | 196 | | | | | | | | | | | | | | | | | | | | | | | | | | 51.4 | | | | | | | | | | | | | | | | | | | | | | 47 | | | | | | | | | | | | | | | | | | 48.5 | | | | | | | | | | | | | | | | | 0.599 | | | |
|  | | | | | | | | | | | | | | | | | | | | | | | | | | | | | | | | | | | | Ground nut oil | | | | | | | | | | | | | | | | | | | | | | | | | | | | | | | | | | | | | | | | | | | 18 | | | | | | | | | | | | | | | | | | | | | | | | | | 4.7 | | | | | | | | | | | | | | | | | | | | | | 12 | | | | | | | | | | | | | | | | | | 12.4 | | | | | | | | | | | | | | | | | 0.001 | | | |
|  | | | | | | | | | | | | | | | | | | | | | | | | | | | | | | | | | | | | Burned oil | | | | | | | | | | | | | | | | | | | | | | | | | | | | | | | | | | | | | | | | | | | 13 | | | | | | | | | | | | | | | | | | | | | | | | | | 3.4 | | | | | | | | | | | | | | | | | | | | | | 3 | | | | | | | | | | | | | | | | | | 3.1 | | | | | | | | | | | | | | | | | 1.000 | | | |
|  | | | | | | | | | | | | | | | | | | | | | | | | | | | | | | | | | | | | Sesame oil | | | | | | | | | | | | | | | | | | | | | | | | | | | | | | | | | | | | | | | | | | | 11 | | | | | | | | | | | | | | | | | | | | | | | | | | 2.9 | | | | | | | | | | | | | | | | | | | | | | 2 | | | | | | | | | | | | | | | | | | 2.1 | | | | | | | | | | | | | | | | | 1.000 | | | |
|  | | | | | | | | | | | | | | | | | | | | | | | | | | | | | | | | | | | | Gasoline | | | | | | | | | | | | | | | | | | | | | | | | | | | | | | | | | | | | | | | | | | | 2 | | | | | | | | | | | | | | | | | | | | | | | | | | 0.5 | | | | | | | | | | | | | | | | | | | | | | 0 | | | | | | | | | | | | | | | | | | 0.0 | | | | | | | | | | | | | | | | | 1.000 | | | |
| Other repellent for animals | | | | | | | | | | | | | | | | | | | | | | | | | | | | | | | | | | | | | | | | | | | | | | | Daily | | | | | | | | | | | | | | | | | | | | | | | | | | | | | | | | 137 | | | | | | | | | | | | | | | | | | | | | | | | | | 21.2 | | | | | | | | | | | | | | | | | | | | | | 27 | | | | | | | | | | | | | | | | | | 18.0 | | | | | | | | | | | | | | | | | 0.269 | | | |
| (n=796) | | | | | | | | | | | | | | | | | | | | | | | | | | | | | | | | | | | | | | | | | | | | | | | Frequently | | | | | | | | | | | | | | | | | | | | | | | | | | | | | | | | 23 | | | | | | | | | | | | | | | | | | | | | | | | | | 3.6 | | | | | | | | | | | | | | | | | | | | | | 11 | | | | | | | | | | | | | | | | | | 7.3 | | | | | | | | | | | | | | | | |  | | | |
|  | | | | | | | | | | | | | | | | | | | | | | | | | | | | | | | | | | | | | | | | | | | | | | | Sometimes | | | | | | | | | | | | | | | | | | | | | | | | | | | | | | | | 149 | | | | | | | | | | | | | | | | | | | | | | | | | | 23.1 | | | | | | | | | | | | | | | | | | | | | | 38 | | | | | | | | | | | | | | | | | | 25.3 | | | | | | | | | | | | | | | | |  | | | |
|  | | | | | | | | | | | | | | | | | | | | | | | | | | | | | | | | | | | | | | | | | | | | | | | Rarely | | | | | | | | | | | | | | | | | | | | | | | | | | | | | | | | 11 | | | | | | | | | | | | | | | | | | | | | | | | | | 1.7 | | | | | | | | | | | | | | | | | | | | | | 2 | | | | | | | | | | | | | | | | | | 1.3 | | | | | | | | | | | | | | | | |  | | | |
|  | | | | | | | | | | | | | | | | | | | | | | | | | | | | | | | | | | | | | | | | | | | | | | | Never | | | | | | | | | | | | | | | | | | | | | | | | | | | | | | | | 326 | | | | | | | | | | | | | | | | | | | | | | | | | | 50.5 | | | | | | | | | | | | | | | | | | | | | | 72 | | | | | | | | | | | | | | | | | | 48.0 | | | | | | | | | | | | | | | | |  | | | |
| Type of other repellent | | | | | | | | | | | | | | | | | | | | | | | | | | | | | | | | | | | | | | | | | | | | | | |  | | | | | | | | | | | | | | | | | | | | | | | | | | | | | | | |  | | | | | | | | | | | | | | | | | | | | | | | | | |  | | | | | | | | | | | | | | | | | | | | | |  | | | | | | | | | | | | | | | | | |  | | | | | | | | | | | | | | | | |  | | | |
| (n=398) | | | | | | | | | | | | | | | Smoke from grass | | | | | | | | | | | | | | | | | | | | | | | | | | | | | | | | | | | | | | | | | | | | | | | | | | | | | | | | | | | | | | | | 246 | | | | | | | | | | | | | | | | | | | | | | | | | | 76.9 | | | | | | | | | | | | | | | | | | | | | | 60 | | | | | | | | | | | | | | | | | | 76.9 | | | | | | | | | | | | | | | | | 0.922 | | | |
|  | | | | | | | | | | | | | | | Smoke from wood | | | | | | | | | | | | | | | | | | | | | | | | | | | | | | | | | | | | | | | | | | | | | | | | | | | | | | | | | | | | | | | | 8 | | | | | | | | | | | | | | | | | | | | | | | | | | 2.5 | | | | | | | | | | | | | | | | | | | | | | 1 | | | | | | | | | | | | | | | | | | 1.3 | | | | | | | | | | | | | | | | |  | | | |
|  | | | | | | | | | | | | | | | Smoke from grass and wood | | | | | | | | | | | | | | | | | | | | | | | | | | | | | | | | | | | | | | | | | | | | | | | | | | | | | | | | | | | | | | | | 63 | | | | | | | | | | | | | | | | | | | | | | | | | | 19.7 | | | | | | | | | | | | | | | | | | | | | | 17 | | | | | | | | | | | | | | | | | | 21.8 | | | | | | | | | | | | | | | | |  | | | |
|  | | | | | | | | | | | | | | | Other | | | | | | | | | | | | | | | | | | | | | | | | | | | | | | | | | | | | | | | | | | | | | | | | | | | | | | | | | | | | | | | | 3 | | | | | | | | | | | | | | | | | | | | | | | | | | 0.9 | | | | | | | | | | | | | | | | | | | | | | 0 | | | | | | | | | | | | | | | | | | 0.0 | | | | | | | | | | | | | | | | |  | | | |
|  | | | | | | | | | | | | | | | | | | | | | | | | | | | | | | | | | | | | | | | | | | | | | | | | | | | | | | | | | | | | | | | | | | | | | | | | | | | | | | | | | | | | | | | | | | | | | | | | | | | | | | | | | | | | | | | | | | | | | | | | | | | | | | | | | | | | | | | | | | | | | | | | | | | | | | | | | | | | | | | | | | | | | |
| **Water bodies** | | | | | | | | | | | | | | | | | |  | | | | | | | | | | | | | | | | | | | | | | | | | | | | | | | | | | | | | | | | | | | | | | | | | | | | | | | | | | | |  | | | | | | | | | | | | | | | | | | | | |  | | | | | | | | | | | | | | | | | | | | | |  | | | | | | | | | | | | | | | | | | | |  | | | | | | | | | | | | | | | | | | |  | | | | | |
| In the yard | | | | | | | | | | | | | | | | | | No | | | | | | | | | | | | | | | | | | | | | | | | | | | | | | | | | | | | | | | | | | | | | | | | | | | | | | | | | | | | 616 | | | | | | | | | | | | | | | | | | | | | 76.9 | | | | | | | | | | | | | | | | | | | | | | 142 | | | | | | | | | | | | | | | | | | | | 71.7 | | | | | | | | | | | | | | | | | | | 0.148 | | | | | |
|  | | | | | | | | | | | | | | | | | | Yes, local sewage | | | | | | | | | | | | | | | | | | | | | | | | | | | | | | | | | | | | | | | | | | | | | | | | | | | | | | | | | | | | 115 | | | | | | | | | | | | | | | | | | | | | 14.4 | | | | | | | | | | | | | | | | | | | | | | 30 | | | | | | | | | | | | | | | | | | | | 15.2 | | | | | | | | | | | | | | | | | | |  | | | | | |
|  | | | | | | | | | | | | | | | | | | Yes, stagnant rainwater | | | | | | | | | | | | | | | | | | | | | | | | | | | | | | | | | | | | | | | | | | | | | | | | | | | | | | | | | | | | 70 | | | | | | | | | | | | | | | | | | | | | 8.7 | | | | | | | | | | | | | | | | | | | | | | 26 | | | | | | | | | | | | | | | | | | | | 13.1 | | | | | | | | | | | | | | | | | | |  | | | | | |
| In the immediate surroundings of the yard | | | | | | | | | | | | | | | | | | | | | | | | | | | | | | | | | | | | | | | | | | | | | | | | | | | | | | | | | | | | | | | |  | | | | | | | | | | | | | |  | | | | | | | | | | | | | | | | | | | | |  | | | | | | | | | | | | | | | | | | | | | |  | | | | | | | | | | | | | | | | | | | |  | | | | | | | | | | | | | | | | | | | 0.001 | | | | | |
| (1 don't know) | | | | | | | | | | | | | | | | | | No | | | | | | | | | | | | | | | | | | | | | | | | | | | | | | | | | | | | | | | | | | | | | | | | | | | | | | | | | | | | 357 | | | | | | | | | | | | | | | | | | | | | 44.6 | | | | | | | | | | | | | | | | | | | | | | 71 | | | | | | | | | | | | | | | | | | | | 35.9 | | | | | | | | | | | | | | | | | | |  | | | | | |
|  | | | | | | | | | | | | | | | | | | Yes, pond | | | | | | | | | | | | | | | | | | | | | | | | | | | | | | | | | | | | | | | | | | | | | | | | | | | | | | | | | | | | 432 | | | | | | | | | | | | | | | | | | | | | 54.0 | | | | | | | | | | | | | | | | | | | | | | 121 | | | | | | | | | | | | | | | | | | | | 61.1 | | | | | | | | | | | | | | | | | | |  | | | | | |
|  | | | | | | | | | | | | | | | | | | Yes, river, stream | | | | | | | | | | | | | | | | | | | | | | | | | | | | | | | | | | | | | | | | | | | | | | | | | | | | | | | | | | | | 10 | | | | | | | | | | | | | | | | | | | | | 1.3 | | | | | | | | | | | | | | | | | | | | | | 1 | | | | | | | | | | | | | | | | | | | | 0.5 | | | | | | | | | | | | | | | | | | |  | | | | | |
|  | | | | | | | | | | | | | | | | | | Yes, stagnant rainwater | | | | | | | | | | | | | | | | | | | | | | | | | | | | | | | | | | | | | | | | | | | | | | | | | | | | | | | | | | | | 1 | | | | | | | | | | | | | | | | | | | | | 0.1 | | | | | | | | | | | | | | | | | | | | | | 5 | | | | | | | | | | | | | | | | | | | | 2.5 | | | | | | | | | | | | | | | | | | |  | | | | | |
| Thematic section (household level): Animals and water bodies in the yard **in the dry season** | | | | | | | | | | | | | | | | | | | | | | | | | | | | | | | | | | | | | | | | | | | | | | | | | | | | | | | | | | | | | | | | | | | | | | | | | | | | | | | | | | | | | | | | | | | | | | | | | | | | | | | | | | | | | | | | | | | | | | | | | | | | | | | | | | | | | | | | | | | | | | | | | | | | | | | | | | | | | | | | | | | | | |
| Animals in room at night *(mainly poultry, dog, goat)* | | | | | | | | | | | | | | | | | | | | | | | | | | | | | | | | | | | | | | | | | | | | | | | | | | | | | | | | | | | | | | | | | | | | | | | | | | | | | | 161 | | | | | | | | | | | | | | | | | | | | | 20.1 | | | | | | | | | | | | | | | | | | | | | | 38 | | | | | | | | | | | | | | | | | | | | 19.2 | | | | | | | | | | | | | | | | | | | 0.775 | | | | | |
| Rats/rodents in the room at night | | | | | | | | | | | | | | | | | | | | | | | | | | | | | | | | | | | | | | | | | | | | | | | | | | | | | | | | | | | | | | | | | | | | | | | | | | | | | | 301 | | | | | | | | | | | | | | | | | | | | | 37.6 | | | | | | | | | | | | | | | | | | | | | | 67 | | | | | | | | | | | | | | | | | | | | 33.8 | | | | | | | | | | | | | | | | | | | 0.329 | | | | | |
| Animal burrows in room at night | | | | | | | | | | | | | | | | | | | | | | | | | | | | | | | | | | | | | | | | | | | | | | | | | No | | | | | | | | | | | | | | | | | | | | | | | | | | | | | 477 | | | | | | | | | | | | | | | | | | | | | 59.6 | | | | | | | | | | | | | | | | | | | | | | 121 | | | | | | | | | | | | | | | | | | | | 61.1 | | | | | | | | | | | | | | | | | | | 0.275 | | | | | |
|  | | | | | | | | | | | | | | | | | | | | | | | | | | | | | | | | | | | | | | | | | | | | | | | | | Yes, not many | | | | | | | | | | | | | | | | | | | | | | | | | | | | | 101 | | | | | | | | | | | | | | | | | | | | | 12.6 | | | | | | | | | | | | | | | | | | | | | | 17 | | | | | | | | | | | | | | | | | | | | 8.6 | | | | | | | | | | | | | | | | | | |  | | | | | |
|  | | | | | | | | | | | | | | | | | | | | | | | | | | | | | | | | | | | | | | | | | | | | | | | | | Yes, many | | | | | | | | | | | | | | | | | | | | | | | | | | | | | 223 | | | | | | | | | | | | | | | | | | | | | 27.8 | | | | | | | | | | | | | | | | | | | | | | 60 | | | | | | | | | | | | | | | | | | | | 30.3 | | | | | | | | | | | | | | | | | | |  | | | | | |
| Animals in the yard at night | | | | | | | | | | | | | | | | | | | | | | | | | | | | | | | | | | | | | | | | | | | | | | | | |  | | | | | | | | | | | | | | | | | | | | | | | | | | | | | 687 | | | | | | | | | | | | | | | | | | | | | 85.8 | | | | | | | | | | | | | | | | | | | | | | 157 | | | | | | | | | | | | | | | | | | | | 79.3 | | | | | | | | | | | | | | | | | | | 0.024 | | | | | |
|  | | | | | | | | | | | | | | | | | | *Donkey* | | | | | | | | | | | | | | | | | | | | | | | | | | | | | | | | | | | | | | | | | | | | | | | | | | | | | | | | | | | | *557* | | | | | | | | | | | | | | | | | | | | | *60.5* | | | | | | | | | | | | | | | | | | | | | | *135* | | | | | | | | | | | | | | | | | | | | *68.2* | | | | | | | | | | | | | | | | | | | *0.711* | | | | | |
|  | | | | | | | | | | | | | | | | | | *Goat* | | | | | | | | | | | | | | | | | | | | | | | | | | | | | | | | | | | | | | | | | | | | | | | | | | | | | | | | | | | | *345* | | | | | | | | | | | | | | | | | | | | | *43.1* | | | | | | | | | | | | | | | | | | | | | | *78* | | | | | | | | | | | | | | | | | | | | *39.4* | | | | | | | | | | | | | | | | | | | *0.348* | | | | | |
|  | | | | | | | | | | | | | | | | | | *Sheep* | | | | | | | | | | | | | | | | | | | | | | | | | | | | | | | | | | | | | | | | | | | | | | | | | | | | | | | | | | | | *335* | | | | | | | | | | | | | | | | | | | | | *41.8* | | | | | | | | | | | | | | | | | | | | | | *68* | | | | | | | | | | | | | | | | | | | | *34.3* | | | | | | | | | | | | | | | | | | | *0.055* | | | | | |
|  | | | | | | | | | | | | | | | | | | *Cattle* | | | | | | | | | | | | | | | | | | | | | | | | | | | | | | | | | | | | | | | | | | | | | | | | | | | | | | | | | | | | *182* | | | | | | | | | | | | | | | | | | | | | *22.7* | | | | | | | | | | | | | | | | | | | | | | *39* | | | | | | | | | | | | | | | | | | | | *19.7* | | | | | | | | | | | | | | | | | | | *0.359* | | | | | |
|  | | | | | | | | | | | | | | | | | | *Dog* | | | | | | | | | | | | | | | | | | | | | | | | | | | | | | | | | | | | | | | | | | | | | | | | | | | | | | | | | | | | *60* | | | | | | | | | | | | | | | | | | | | | *7.5* | | | | | | | | | | | | | | | | | | | | | | *21* | | | | | | | | | | | | | | | | | | | | *10.6* | | | | | | | | | | | | | | | | | | | *0.150* | | | | | |
|  | | | | | | | | | | | | | | | | | | *Camel* | | | | | | | | | | | | | | | | | | | | | | | | | | | | | | | | | | | | | | | | | | | | | | | | | | | | | | | | | | | | *4* | | | | | | | | | | | | | | | | | | | | | *0.5* | | | | | | | | | | | | | | | | | | | | | | *0* | | | | | | | | | | | | | | | | | | | | *0.0* | | | | | | | | | | | | | | | | | | | *1.000* | | | | | |
| Animals in adjacent yard at night(1 don't know) | | | | | | | | | | | | | | | | | | | | | | | | | | | | | | | | | | | | | | | | | | | | | | | | | | | | | | | | | | | | | | | | | | | | | | |  | | | | | | | 749 | | | | | | | | | | | | | | | | | | | | | 93.5 | | | | | | | | | | | | | | | | | | | | | | 179 | | | | | | | | | | | | | | | | | | | | 90.4 | | | | | | | | | | | | | | | | | | | 0.112 | | | | | |
|  | | | | | | | | | | | | | | | | | | Donkey | | | | | | | | | | | | | | | | | | | | | | | | | | | | | | | | | | | | | | | | | | | | | | | | | | | | | | | | | | | | *723* | | | | | | | | | | | | | | | | | | | | | *90.3* | | | | | | | | | | | | | | | | | | | | | | *168* | | | | | | | | | | | | | | | | | | | | *84.9* | | | | | | | | | | | | | | | | | | | *0.028* | | | | | |
|  | | | | | | | | | | | | | | | | | | Goat | | | | | | | | | | | | | | | | | | | | | | | | | | | | | | | | | | | | | | | | | | | | | | | | | | | | | | | | | | | | *614* | | | | | | | | | | | | | | | | | | | | | *76.7* | | | | | | | | | | | | | | | | | | | | | | *143* | | | | | | | | | | | | | | | | | | | | *72.2* | | | | | | | | | | | | | | | | | | | *0.192* | | | | | |
|  | | | | | | | | | | | | | | | | | | Sheep | | | | | | | | | | | | | | | | | | | | | | | | | | | | | | | | | | | | | | | | | | | | | | | | | | | | | | | | | | | | *552* | | | | | | | | | | | | | | | | | | | | | *68.9* | | | | | | | | | | | | | | | | | | | | | | *132* | | | | | | | | | | | | | | | | | | | | *66.7* | | | | | | | | | | | | | | | | | | | *0.542* | | | | | |
|  | | | | | | | | | | | | | | | | | | Cattle | | | | | | | | | | | | | | | | | | | | | | | | | | | | | | | | | | | | | | | | | | | | | | | | | | | | | | | | | | | | *341* | | | | | | | | | | | | | | | | | | | | | *42.6* | | | | | | | | | | | | | | | | | | | | | | *87* | | | | | | | | | | | | | | | | | | | | *43.9* | | | | | | | | | | | | | | | | | | | *0.728* | | | | | |
|  | | | | | | | | | | | | | | | | | | Dog | | | | | | | | | | | | | | | | | | | | | | | | | | | | | | | | | | | | | | | | | | | | | | | | | | | | | | | | | | | | *154* | | | | | | | | | | | | | | | | | | | | | *19.2* | | | | | | | | | | | | | | | | | | | | | | *44* | | | | | | | | | | | | | | | | | | | | *22.2* | | | | | | | | | | | | | | | | | | | *0.344* | | | | | |
|  | | | | | | | | | | | | | | | | | | Camel | | | | | | | | | | | | | | | | | | | | | | | | | | | | | | | | | | | | | | | | | | | | | | | | | | | | | | | | | | | | *13* | | | | | | | | | | | | | | | | | | | | | *1.6* | | | | | | | | | | | | | | | | | | | | | | *2* | | | | | | | | | | | | | | | | | | | | *1.0* | | | | | | | | | | | | | | | | | | | *0.525* | | | | | |
|  | | | | | | | | | | | | | | | | | | Horse | | | | | | | | | | | | | | | | | | | | | | | | | | | | | | | | | | | | | | | | | | | | | | | | | | | | | | | | | | | | *1* | | | | | | | | | | | | | | | | | | | | | *0.1* | | | | | | | | | | | | | | | | | | | | | | *0* | | | | | | | | | | | | | | | | | | | | *0.0* | | | | | | | | | | | | | | | | | | | *1* | | | | | |
| Uncovered animal burrows in the yard | | | | | | | | | | | | | | | | | | | | | | | | | | | | | | | | | | | | | | | | | | | | | | | | | | | | | | | | | | | | | | | | | | | | | | | | | | | | | |  | | | | | | | | | | | | | | | | | | | | |  | | | | | | | | | | | | | | | | | | | | | |  | | | | | | | | | | | | | | | | | | | |  | | | | | | | | | | | | | | | | | | | 0.537 | | | | | |
| (2 don't know) | | | | | | | | | | | | | | | | | | No | | | | | | | | | | | | | | | | | | | | | | | | | | | | | | | | | | | | | | | | | | | | | | | | | | | | | | | | | | | | 107 | | | | | | | | | | | | | | | | | | | | | 13.4 | | | | | | | | | | | | | | | | | | | | | | 21 | | | | | | | | | | | | | | | | | | | | 10.6 | | | | | | | | | | | | | | | | | | |  | | | | | |
|  | | | | | | | | | | | | | | | | | | Yes, but not many | | | | | | | | | | | | | | | | | | | | | | | | | | | | | | | | | | | | | | | | | | | | | | | | | | | | | | | | | | | | 65 | | | | | | | | | | | | | | | | | | | | | 8.1 | | | | | | | | | | | | | | | | | | | | | | 15 | | | | | | | | | | | | | | | | | | | | 7.6 | | | | | | | | | | | | | | | | | | |  | | | | | |
|  | | | | | | | | | | | | | | | | | | Yes, many | | | | | | | | | | | | | | | | | | | | | | | | | | | | | | | | | | | | | | | | | | | | | | | | | | | | | | | | | | | | 627 | | | | | | | | | | | | | | | | | | | | | 78.5 | | | | | | | | | | | | | | | | | | | | | | 162 | | | | | | | | | | | | | | | | | | | | 81.8 | | | | | | | | | | | | | | | | | | |  | | | | | |
| **Use of repellent for the body of animals (if animals in the yard) in the dry season** | | | | | | | | | | | | | | | | | | | | | | | | | | | | | | | | | | | | | | | | | | | | | | | | | | | | | | | | | | | | | | | | | | | | | | | | | | | | | | | | | | | | | | | | | | | | | | | | | | | | | | | | | | | | | | | | | | | | | | | | | | | | | | | | | | | | | | | | | | | | | | | | | | | | | | | | | | | | | | | | | | | | | |
| (n=792) | | | | | | | | | | | | | | | | | | | | | | | Daily | | | | | | | | | | | | | | | | | | | | | | | | | | | | | | | | | | | | | | | | | | | | | 2 | | | | | | | | | | | | | | | | | | | | | | | | | | | 0.3 | | | | | | | | | | | | | | | | | | | 2 | | | | | | | | | | | | | | | | | | | | | | | 1.3 | | | | | | | | | | | | | | | | | | | 0.412 | | | | | | | | | |
|  | | | | | | | | | | | | | | | | | | | | | | | Frequently | | | | | | | | | | | | | | | | | | | | | | | | | | | | | | | | | | | | | | | | | | | | | 20 | | | | | | | | | | | | | | | | | | | | | | | | | | | 3.1 | | | | | | | | | | | | | | | | | | | 6 | | | | | | | | | | | | | | | | | | | | | | | 4.0 | | | | | | | | | | | | | | | | | | |  | | | | | | | | | |
|  | | | | | | | | | | | | | | | | | | | | | | | Sometimes | | | | | | | | | | | | | | | | | | | | | | | | | | | | | | | | | | | | | | | | | | | | | 164 | | | | | | | | | | | | | | | | | | | | | | | | | | | 25.5 | | | | | | | | | | | | | | | | | | | 43 | | | | | | | | | | | | | | | | | | | | | | | 28.9 | | | | | | | | | | | | | | | | | | |  | | | | | | | | | |
|  | | | | | | | | | | | | | | | | | | | | | | | Rarely | | | | | | | | | | | | | | | | | | | | | | | | | | | | | | | | | | | | | | | | | | | | | 32 | | | | | | | | | | | | | | | | | | | | | | | | | | | 5.0 | | | | | | | | | | | | | | | | | | | 6 | | | | | | | | | | | | | | | | | | | | | | | 4.0 | | | | | | | | | | | | | | | | | | |  | | | | | | | | | |
|  | | | | | | | | | | | | | | | | | | | | | | | Never | | | | | | | | | | | | | | | | | | | | | | | | | | | | | | | | | | | | | | | | | | | | | 425 | | | | | | | | | | | | | | | | | | | | | | | | | | | 66.1 | | | | | | | | | | | | | | | | | | | 92 | | | | | | | | | | | | | | | | | | | | | | | 61.7 | | | | | | | | | | | | | | | | | | |  | | | | | | | | | |
| Type of animal repellent | | | | | | | | | | | | | | | | | | | | | | | | | | | | | | | | | | | | Chemical | | | | | | | | | | | | | | | | | | | | | | | | | | | | | | | | 130 | | | | | | | | | | | | | | | | | | | | | | | | | | | 58.8 | | | | | | | | | | | | | | | | | | | 27 | | | | | | | | | | | | | | | | | | | | | | | 47.4 | | | | | | | | | | | | | | | | | | | 0.120 | | | | | | | | | |
| (n=278) | | | | | | | | | | | | | | | | | | | | | | | | | | | | | | | | | | | | Local ‘Tar’ (Quotran) | | | | | | | | | | | | | | | | | | | | | | | | | | | | | | | | 91 | | | | | | | | | | | | | | | | | | | | | | | | | | | 41.2 | | | | | | | | | | | | | | | | | | | 24 | | | | | | | | | | | | | | | | | | | | | | | 42.1 | | | | | | | | | | | | | | | | | | | 0.899 | | | | | | | | | |
|  | | | | | | | | | | | | | | | | | | | | | | | | | | | | | | | | | | | | Ground nut oil | | | | | | | | | | | | | | | | | | | | | | | | | | | | | | | | 7 | | | | | | | | | | | | | | | | | | | | | | | | | | | 3.2 | | | | | | | | | | | | | | | | | | | 8 | | | | | | | | | | | | | | | | | | | | | | | 14.0 | | | | | | | | | | | | | | | | | | | 0.004 | | | | | | | | | |
|  | | | | | | | | | | | | | | | | | | | | | | | | | | | | | | | | | | | | Burned oil | | | | | | | | | | | | | | | | | | | | | | | | | | | | | | | | 5 | | | | | | | | | | | | | | | | | | | | | | | | | | | 2.3 | | | | | | | | | | | | | | | | | | | 2 | | | | | | | | | | | | | | | | | | | | | | | 3.5 | | | | | | | | | | | | | | | | | | | 0.635 | | | | | | | | | |
|  | | | | | | | | | | | | | | | | | | | | | | | | | | | | | | | | | | | | Sesame oil | | | | | | | | | | | | | | | | | | | | | | | | | | | | | | | | 1 | | | | | | | | | | | | | | | | | | | | | | | | | | | 0.8 | | | | | | | | | | | | | | | | | | | 0 | | | | | | | | | | | | | | | | | | | | | | | 0.2 | | | | | | | | | | | | | | | | | | | 1.000 | | | | | | | | | |
|  | | | | | | | | | | | | | | | | | | | | | | | | | | | | | | | | | | | | Gasoline | | | | | | | | | | | | | | | | | | | | | | | | | | | | | | | | 2 | | | | | | | | | | | | | | | | | | | | | | | | | | | 0.9 | | | | | | | | | | | | | | | | | | | 0 | | | | | | | | | | | | | | | | | | | | | | | 0.0 | | | | | | | | | | | | | | | | | | | 1.000 | | | | | | | | | |
| Other repellent for animals | | | | | | | | | | | | | | | | | | | | | | | | | | | | | | | | | | | | | | | | | | | | | | | Daily | | | | | | | | | | | | | | | | | | | | | 1 | | | | | | | | | | | | | | | | | | | | | | | | | | | 0.2 | | | | | | | | | | | | | | | | | | | 2 | | | | | | | | | | | | | | | | | | | | | | | 1.3 | | | | | | | | | | | | | | | | | | | 0.560 | | | | | | | | | |
| (n=789) | | | | | | | | | | | | | | | | | | | | | | | | | | | | | | | | | | | | | | | | | | | | | | | Frequently | | | | | | | | | | | | | | | | | | | | | 1 | | | | | | | | | | | | | | | | | | | | | | | | | | | 0.2 | | | | | | | | | | | | | | | | | | | 6 | | | | | | | | | | | | | | | | | | | | | | | 4.0 | | | | | | | | | | | | | | | | | | |  | | | | | | | | | |
|  | | | | | | | | | | | | | | | | | | | | | | | | | | | | | | | | | | | | | | | | | | | | | | | Sometimes | | | | | | | | | | | | | | | | | | | | | 14 | | | | | | | | | | | | | | | | | | | | | | | | | | | 2.2 | | | | | | | | | | | | | | | | | | | 43 | | | | | | | | | | | | | | | | | | | | | | | 28.9 | | | | | | | | | | | | | | | | | | |  | | | | | | | | | |
|  | | | | | | | | | | | | | | | | | | | | | | | | | | | | | | | | | | | | | | | | | | | | | | | Rarely | | | | | | | | | | | | | | | | | | | | | 5 | | | | | | | | | | | | | | | | | | | | | | | | | | | 0.8 | | | | | | | | | | | | | | | | | | | 6 | | | | | | | | | | | | | | | | | | | | | | | 4.0 | | | | | | | | | | | | | | | | | | |  | | | | | | | | | |
|  | | | | | | | | | | | | | | | | | | | | | | | | | | | | | | | | | | | | | | | | | | | | | | | Never | | | | | | | | | | | | | | | | | | | | | 619 | | | | | | | | | | | | | | | | | | | | | | | | | | | 96.7 | | | | | | | | | | | | | | | | | | | 92 | | | | | | | | | | | | | | | | | | | | | | | 61.7 | | | | | | | | | | | | | | | | | | |  | | | | | | | | | |
| Type of other repellent (n=24) | | | | | | | | | | | | | | | | | | | | Smoke from grass | | | | | | | | | | | | | | | | | | | | | | | | | | | | | | | | | | | | | | | | | | | | | | | | 18 | | | | | | | | | | | | | | | | | | | | | | | | | | | 85.7 | | | | | | | | | | | | | | | | | | | 3 | | | | | | | | | | | | | | | | | | | | | | | 100.0 | | | | | | | | | | | | | | | | | | | 1.000 | | | | | | | | | |
|  |  |  |  |  |  |  |  |  |  |  |  |  |  |  |  |  |  |  |  | Smoke from grass/wood | | | | | | | | | | | | | | | | | | | | | | | | | | | | | | | | | | | | | | | | | | | | | | | | 3 | | | | | | | | | | | | | | | | | | | | | | | | | | | 14.3 | | | | | | | | | | | | | | | | | | | 0 | | | | | | | | | | | | | | | | | | | | | | | 0.0 | | | | | | | | | | | | | | | | | | |  | | | | | | | | | |
| **Water bodies** | | | | | | | | | | | | | | | | | | | |  | | | | | | | | | | | | | | | | | | | | | | | | | | | | | | | | | | | | | | | | | | | | | | | |  | | | | | | | | | | | | | | | | | | | | | | | | | | |  | | | | | | | | | | | | | | | | | | |  | | | | | | | | | | | | | | | | | | | | | | |  | | | | | | | | | | | | | | | | | | |  | | | | | | | | | |
| In the yard | | | | | | | | | | | | | | | | | | | | No | | | | | | | | | | | | | | | | | | | | | | | | | | | | | | | | | | | | | | | | | | | | | | | | 799 | | | | | | | | | | | | | | | | | | | | | | | | | | | 99.8 | | | | | | | | | | | | | | | | | | | 198 | | | | | | | | | | | | | | | | | | | | | | | 100.0 | | | | | | | | | | | | | | | | | | | 1.000 | | | | | | | | | |
|  | | | | | | | | | | | | | | | | | | | | Yes, local sewage | | | | | | | | | | | | | | | | | | | | | | | | | | | | | | | | | | | | | | | | | | | | | | | | 1 | | | | | | | | | | | | | | | | | | | | | | | | | | | 0.1 | | | | | | | | | | | | | | | | | | | 0 | | | | | | | | | | | | | | | | | | | | | | | 0.0 | | | | | | | | | | | | | | | | | | |  | | | | | | | | | |
|  | | | | | | | | | | | | | | | | | | | | Yes, stagnant rainwater | | | | | | | | | | | | | | | | | | | | | | | | | | | | | | | | | | | | | | | | | | | | | | | | 1 | | | | | | | | | | | | | | | | | | | | | | | | | | | 0.1 | | | | | | | | | | | | | | | | | | | 0 | | | | | | | | | | | | | | | | | | | | | | | 0.0 | | | | | | | | | | | | | | | | | | |  | | | | | | | | | |
| In the immediate surroundings of the yard | | | | | | | | | | | | | | | | | | | | | | | | | | | | | | | | | | | | | | | | | | | | | | | | | | | | | | | | | | | | | | | | | | | |  | | | | | | | | | | | | | | | | | | | | | | | | | | |  | | | | | | | | | | | | | | | | | | |  | | | | | | | | | | | | | | | | | | | | | | |  | | | | | | | | | | | | | | | | | | | 0.731 | | | | | | | | | |
| (1 don't know) | | | | | | | | | | | | | | | | | | | | No | | | | | | | | | | | | | | | | | | | | | | | | | | | | | | | | | | | | | | | | | | | | | | | | 787 | | | | | | | | | | | | | | | | | | | | | | | | | | | 98.3 | | | | | | | | | | | | | | | | | | | 197 | | | | | | | | | | | | | | | | | | | | | | | 99.5 | | | | | | | | | | | | | | | | | | |  | | | | | | | | | |
|  | | | | | | | | | | | | | | | | | | | | Yes, pond | | | | | | | | | | | | | | | | | | | | | | | | | | | | | | | | | | | | | | | | | | | | | | | | 11 | | | | | | | | | | | | | | | | | | | | | | | | | | | 1.4 | | | | | | | | | | | | | | | | | | | 1 | | | | | | | | | | | | | | | | | | | | | | | 0.5 | | | | | | | | | | | | | | | | | | |  | | | | | | | | | |
|  | | | | | | | | | | | | | | | | | | | | Yes, river, stream | | | | | | | | | | | | | | | | | | | | | | | | | | | | | | | | | | | | | | | | | | | | | | | | 1 | | | | | | | | | | | | | | | | | | | | | | | | | | | 0.1 | | | | | | | | | | | | | | | | | | | 0 | | | | | | | | | | | | | | | | | | | | | | | 0.0 | | | | | | | | | | | | | | | | | | |  | | | | | | | | | |
|  | | | | | | | | | | | | | | | | | | | | Yes, stagnant rainwater | | | | | | | | | | | | | | | | | | | | | | | | | | | | | | | | | | | | | | | | | | | | | | | | 2 | | | | | | | | | | | | | | | | | | | | | | | | | | | 0.3 | | | | | | | | | | | | | | | | | | | 0 | | | | | | | | | | | | | | | | | | | | | | | 0.0 | | | | | | | | | | | | | | | | | | |  | | | | | | | | | |

1. The question was about spraying done before the beginning of symptoms for cases. However, reverse cause bias cannot be excluded. [↑](#footnote-ref-2)
2. The question was about trees cut before the beginning of symptoms for cases. However, reverse cause bias cannot be excluded. [↑](#footnote-ref-3)
