# Supplementary material for: Determinants of Visceral Leishmaniasis: A Case-Control Study in Gedaref State, Sudan
Source: PLoS Negl Trop Dis. 2015 Nov 6;9(11):e0004187. doi: 10.1371/journal.pntd.0004187 (PMC4636291; doi:10.1371/journal.pntd.0004187)
Supplement: S2 Table — (DOC) [file pntd.0004187.s003.doc]

**Table 3. Multivariate models of individual and household determinants for visceral leishmaniasis (with village as a random effect). Gedaref, Sudan, 2012-2013.**

|  |  | **Unweighted logistic regression mixed model** | | | | | **Generalised latent and mixed model weighted for number of eligible controls in the household** | | | |
| --- | --- | --- | --- | --- | --- | --- | --- | --- | --- | --- |
| (N=998) |  | OR* | | 95%CI* | | p-value* | OR § | | 95%CI § | |
| **Gender,** *in <10 years* | Male | 1 |  | |  | <0.001 | 1 |  | |  |
|  | Female | 0.38 | 0.23 | | 0.65 |  | 0.42 | 0.25 | | 0.69 |
| **Gender,** *≥ 10 years* | Male | 1 |  | |  | <0.001 | 1 |  | |  |
|  | Female | 0.51 | 0.34 | | 0.78 |  | 0.56 | 0.37 | | 0.85 |
| **Age,** *per 10 years, among males* |  | 0.53 | 0.23 | | 0.65 | <0.001 | 0.55 | 0.40 | | 0.75 |
| **Age,** *per 10 years, among females* | | 0.71 | 0.59 | | 0.85 | <0.001 | 0.74 | 0.63 | | 0.88 |
| **Main evening outdoor activities**** | Stay indoor only | 1 |  | |  | <0.001 | 1 |  | |  |
|  | Playing | 1.77 | 1.08 | | 2.90 |  | 1.63 | 1.11 | | 2.40 |
|  | TV / radio | 3.84 | 1.92 | | 7.68 |  | 4.66 | 2.22 | | 10.27 |
|  | Discussing-relaxing | 1.44 | 0.85 | | 2.45 |  | 1.65 | 1.00 | | 2.72 |
|  | Cooking/house activities | 0.55 | 0.17 | | 1.83 |  | 0.56 | 0.19 | | 1.62 |
|  | Other activities | 0.39 | 0.08 | | 1.97 |  | 0.56 | 1.16 | | 1.95 |
| **Sleep location**** | In the house yard | 1 |  | |  | 0.002 | 1 |  | |  |
|  | Outside the house yard | 3.91 | 1.70 | | 9.00 |  | 4.49 | 2.14 | | 9.42 |
| **Never sleeping in the field/farm**** | | 2.62 | 1.32 | | 5.17 | 0.004 | 2.78 | 1.13 | | 6.84 |
| **Household size** | *(increase of one person)* | 1.07 | 1.01 | | 1.13 | 0.024 | 1.05 | 1.01 | | 1.09 |
| **Source of drinking water**  *(dry season)* | Village water tank | 1 |  | |  | 0.014 | 1 |  | |  |
|  | River/surface water | 0.36 | 0.18 | | 0.71 |  | 0.28 | 0.19 | | 0.41 |
|  | Pump, well, water sellers | 0.63 | 0.40 | | 0.99 |  | 0.64 | 0.42 | | 0.97 |
| **Forest at eye range** | | 1.71 | 1.14 | | 2.55 | 0.009 | 1.90 | 1.35 | | 2.69 |
| ***Acacia nilotica* in the surroundings of the yard** | | 1.72 | 1.17 | | 2.53 | 0.006 | 1.86 | 1.20 | | 2.89 |
| **Distance to the closest house** | Share a common limit | 1 |  | |  | <0.001 | 1 |  | |  |
|  | Space in between (<10m) | 0.35 | 0.18 | | 0.67 |  | 0.31 | 0.12 | | 0.82 |
|  | More than 10 m | 0.51 | 0.26 | | 1.00 |  | 0.47 | 0.24 | | 0.91 |
| **Animals in the yard at night**** | | 0.56 | 0.34 | | 0.92 | 0.024 | 0.67 | 0.42 | | 0.75 |
| **Dog in the yard at night**** | | 2.43 | 1.27 | | 4.69 | 0.010 | 3.01 | 1.60 | | 5.65 |
| **Smoke from *Acacia seyal* as indoor repellent** | | 1.57 | 1.06 | | 2.32 | 0.024 | 1.60 | 0.85 | | 3.00 |
| **Ground nut oil as animal body repellent** | | 4.15 | 1.65 | | 10.5 | 0.003 | 3.63 | 1.45 | | 9.06 |

OR: Odds Ratio; 95%CI: 95% Confidence Interval; *Adjusted for village as a random effect (14% of variance, 95%CI 5-35%);

** During the rainy season; § Adjusted for village as a random effect and weighted for the number of eligible controls in the household; The level of education of the participant was not included in the model due to collinearity with age.
